# Refined home-brew media for cost-effective, weekend-free hiPSC culture and genetic engineering

**DOI:** 10.12688/openreseurope.18245.1

**Published:** 2024-09-02

**Authors:** Lukasz Truszkowski, Sveva Bottini, Sara Bianchi, Helen Bell, Silvia Becca, Giulia Savorè, Kirsten E Snijders, Federica Sozza, Cristina Rubinetto, Luana Ferrara, Elisa Balmas, Catherine Elton, Alessandro Bertero

**Affiliations:** 1 Department of Molecular Biotechnology and Health Sciences, Molecular Biotechnology Center “Guido Tarone”, University of Turin, Torino, 10126, Italy; 2 Qkine, Cambridge, UK

**Keywords:** hiPSC; pluripotency; culture media; thermostable FGF2; TGF beta

## Abstract

**Background:**

Cost-effective, practical, and reproducible culture of human induced pluripotent stem cells (hiPSCs) is required for both basic and translational research. This is especially crucial for large-scale expansion of hiPSCs for cell therapy, which should be made accessible to many patients regardless of their socioeconomic background. Basal 8 (B8) has emerged as a cost-effective solution for weekend-free and chemically-defined hiPSC culture. However, homebrewing of some recombinant growth factors for B8 can be a bottleneck towards both access and reproducibility of this technology. Moreover, we found the published B8 formulation to be suboptimal in normoxic hiPSC culture, which is widely used. Lastly, the suitability of B8 for applications such as genome editing or organoid differentiation remains to be assessed.

**Methods:**

We formulated B8 with commercially available, animal-free growth factors, refined its composition to support normoxic culture of the widely-used WTC11 hiPSC line, and compared it to commercial Essential 8 (E8) and a home-made, weekend-free E8 formulation (hE8). We measured pluripotency marker expression and cell cycle with flow cytometry, and investigated the transcriptional profiles by bulk RNA sequencing. We also assessed the efficiency of gene editing, single-cell sorting, and cardiac differentiation in both monolayer and organoids.

**Results:**

hE8 performed similarly to commercial E8 in all the assays. Despite morphological changes, cells in B8+, our optimised variant of B8, expressed the pluripotency marker NANOG at the highest level. At the same time, cells grown in B8+ were primed towards a mesendodermal fate. B8+ outperformed other media with regard to genome editing *via* homology directed recombination, and was on par with other media in other assays.

**Conclusions:**

Overall, optimised weekend-free media formulations promise to democratise the generation of engineered cells for a wide range of applications.

## Introduction

Human pluripotent stem cells (hPSCs, either embryo-derived, hESCs, or induced via reprogramming, hiPSCs) have become a prominent tool in biomedical research. Their potential to differentiate towards virtually all somatic cell types offers the unprecedented ability to study rare, transient, and/or difficult to access cell types, such as those of the developing heart. hiPSCs can be readily derived from patients, genome-edited to obtain isogenic controls, and employed for disease modelling and drug screening studies, paving the way towards personalised medicine ^1^.

To achieve safe and reproducible hiPSC culture, formulations for feeder-free, chemically defined media such as mTESR ^2^ or Essential 8 ^3^ have been established. However, commercial sources of these media cost hundreds of euros per litre and require daily media changes. Commercial weekend-free formulations have been developed, but their cost is even higher. In all, culturing hiPSCs, especially on a large scale, can consume a lot of manpower and financial resources. This puts a strong burden on stem cell labs and represents an important roadblock to the adoption of hPSC technology in non-expert groups.

Basal 8 (B8) is a recently reported, weekend-free medium formulation with optimised amounts of growth factors and other media components to reduce medium cost ^4, 5^. The most effective cost-cutting strategy is the in-house production of thermostable FGF2 (FGF2-G3). However, endotoxin contamination and batch-to-batch variation in bacterial FGF2-G3 production can be a challenge for smaller labs. In addition, some applications of B8, such as genome editing or organoid differentiation, remain to be assessed.

In this work, we optimised the concentrations of growth factors purchased commercially for weekend-free media in a standard normoxia culture. Then, we tested two formulations of weekend-free media and compared the performance of hiPSCs growing in these media to cells grown in commercial E8. Our results show that weekend-free media can match or even outperform daily-changed commercial media in all tested applications, while markedly reducing media costs.

## Methods

### hiPSC culture

In all experiments (unless specified otherwise), hiPSCs from the WTC-11 line (RRID: CVCL_Y803), kindly provided by Bruce Conkin (J. David Gladstone Institutes), were cultured feeder-free on dishes coated with Geltrex™ (Gibco, #A1413302) at matrix density of 5.2 µg cm ^-2 ^and maintained in normoxic conditions in the CellXpert C170i copper incubator (Eppendorf) at 37 °C in a humidified atmosphere with 5% of CO _2_. Cells were passaged at 70–80% confluency in the following way: after a wash with PBS without calcium and magnesium (homemade), the cells were treated with 0.5 mM EDTA (Sigma-Aldrich, #4055–100mL) in PBS without calcium and magnesium for 2 min at 37 °C. After aspiration of EDTA, cells were dissociated into small clumps in DMEM-F12 (Gibco, #31330038) and seeded with culture media supplemented with 2 µM thiazovivin (Cayman Chemicals, #004CA14245-25). The cell culture medium was replaced with a fresh culture medium without thiazovivin after 16 h. The cells used in the experiments were at passages 50–70. Cells were checked every two weeks for the presence of mycoplasma.

### Growth factor production

Constructs harbouring the growth factors genes (#Qk025, #Qk027, #Qk052, #Qk053: Uniprot number P09038; #Qk001: Uniprot number P08476; #Qk010: Uniprot number P01137, #Qk054; Uniprot number P10600; #Qk045: Uniprot number Q02297) were expressed using a microbial expression system in a β-lactam-free and animal-free environment. All above mentioned growth factors were purified to homogeneity without the use of tags. The bioactivity and purity of the growth factors were assessed and validated to meet industry quality standards.

### Assay for growth factor stability in conditioned media

HEK293 cells (ATCC) were cultured at 37 °C, 5% CO _2_ in Dulbecco’s Modified Eagle Medium (DMEM; Gibco #31966047) and 10 % foetal bovine serum (FBS; Thermo Fisher Scientific #10500064) and plated onto 96-well plates (Corning, #353072) at a density of 1 × 10 ^4^ cells per well. Cells were left to adhere for 24 h before transfection using FuGENE HD (Promega, #E2311) with 50 ng per well DNA total of pGL4.33[luc2P/SRE/Hygro] (Promega, #E1340) for FGF2-G3, or pGL3-CAGA _12_-luc for TGF-β1. Co-transfection with pRL-TK (FGF2-G3) or pRL-SV40 (TGF-β1) (both from Promega) allowed for constitutive expression of *Renilla* luciferase for normalisation. In the case of transfections including the SRE reporter, cells were starved of serum by replacement of media with Opti-MEM I (Gibco, #51985034) for 18 h prior to assay. FGF2-G3 and t-FGF2-G3 (Qkine, #Qk052, #Qk053) and TGF-β1 (Qkine, #Qk010) were reconstituted in water or 10 mM HCl respectively, and then diluted to a concentration of 0.25 mg/mL in HEK293-conditioned media (filtered media from a 3-day culture of HEK293 cells). This was incubated at 37 °C and samples were taken at the indicated time points, and frozen at -80 °C until required. Proteins were serially diluted in OptiMEM (FGF2-G3) or DMEM with 0.5% FBS (TGF-β1) and added to transfected cells in triplicate, before incubation of the plates at 37 °C. After 3 h (FGF2-G3) or 6 h (TGF-β1), the media was removed, and the cells were lysed with Passive Lysis Buffer (Promega). Firefly and Renilla luciferase readings were performed using the FLUOstar® Omega plate reader (BMG Labtech). Firefly/Renilla ratios were calculated for each well and plotted against the protein concentration. Curve fitting to a 4-parameter logistic equation was performed in Prism 10 (GraphPad - https://www.graphpad.com).

### Culture media

Homemade weekend-free media was prepared with DMEM/F12, L-glutamine and HEPES (Gibco, #31330038), and was supplemented with a 100x growth factors supplement prepared in-house. The final concentrations of the supplement components in the media were as follows (also see Extended Data 4 ^6^ for the preparation protocol): for homemade E8 (hE8): 64 µg mL ^-1^ 2-Phospho-L-ascorbic acid trisodium salt (Sigma, #49752-10G), 14 ng mL ^-1^ sodium selenite (Sigma, #S5261-25G), 1743 µg mL ^-1 ^sodium bicarbonate (Sigma-Aldrich, #S8875-500G), 10 µg mL ^-1^ transferrin (Optiferrin – Invitria, #777TRF029-1G), 19.4 µg mL ^-1^ insulin (Gibco, #A11382II), 5 ng mL ^-1^ t-FGF2-G3 (145aa, Qkine, #Qk052), 2 ng mL ^-1^ TGF-β1 PLUS (Qkine, #Qk010); for B8+: 200 µg mL ^-1^ 2-Phospho-L-ascorbic acid trisodium salt, 20 ng mL ^-1^ sodium selenite (Sigma, #S5261-25G), 1743 µg mL ^-1 ^sodium bicarbonate, 5 µg mL ^-1^ transferrin, 5 µg mL ^-1^ insulin, 5 ng mL ^-1^ t-FGF2-G3, 1 ng mL ^-1^ TGF-β3 (Qkine, #Qk054), 0.1 ng mL ^-1^ NRG-1 (Qkine, #Qk045). Note that since DMEM/F12 already contains sodium bicarbonate at 1200 µg mL ^-1 ^concentration, the supplement contains only enough sodium bicarbonate to increase the concentration to the final one described above. In initial optimisation experiments, B8 formulations had alternative sources of FGF2 or TGF-β signalling pathway, such as FGF2, FGF2-G3, t-FGF2, or Activin A1 (Qkine, #Qk027, #Qk053, #Qk025 or #Qk001, respectively). The weekend-free media were compared to commercial E8 – cE8 (Gibco, #A1517001) that was changed daily.

### Adaptation of the cells into the weekend-free media

For each adaptation round, a vial from the same-passage cells expanded in cE8 was thawed and transferred to 2 mL of DMEM/F12, centrifuged at 100 g for 5 min, then resuspended in 2 mL of cE8 with thiazovivin and seeded into 3 wells of a 6-well plate (Corning, #353046), seeding 50, 25, or 12.5% of the suspension, respectively. The cells were then grown in cE8. The next passage was done in cE8, replacing the medium the day after passage with cE8:hE8 or cE8:B8+ at 1:1 proportion. The following day the medium was replaced with the respective weekend free media (hE8 or B8+). After that, these cells were grown and passaged in hE8 or B8+. In parallel, cells from the same vial were grown in cE8 and passaged at the same time as cells in weekend-free media. Cells were grown for at least five passages before performing any assays.

### Flow cytometry analysis of pluripotency markers

Cells that were ready for passage were detached as described before (treated with 0.5 mM EDTA and dissociated in DMEM/F12) and centrifuged at 100 g for 5 min. The cell pellet was resuspended in 600 µL of PBS and transferred on a 96-well plate with a round bottom (Euroclone, #ET3196). The cells were centrifuged at 200 g for 5 min, resuspended in 100 µL of Fixable Viability Dye eFluor 450 (eBioscience, #65-0863-14) diluted 1:1000 in PBS and incubated at room temperature (RT) for 10 min. After three cycles of washes consisting of resuspending cells in 200 µL of FACS buffer (5% FBS – Life Technologies, #10270106 in PBS) and centrifuging at 200g for 5 min, the cells were fixed in 100 µL of 4% PFA (Thermo Scientific, #J61899.AP) at room temperature (RT) for 2 min. Then, the cells were washed three times as above with FACS buffer and permeabilized in 100 µL FACS buffer with 0.75% saponin (Sigma, #8047-15-2) and 0.1% Triton X-100 (Merck, #T8787-100mL) at RT for 40 min. After centrifuging at 200 g for 5 min, the pellet was resuspended with 50 µL of antibodies diluted in permeabilization buffer. For each sample, OCT4/NANOG double staining and respective isotype control staining were performed. In addition, single stained controls were performed on the pooled samples. The following antibodies were used: PE Mouse anti-Oct3/4 (BD Pharmingen, #560186) at 1:10 dilution, AF 647 Mouse anti-Human Nanog (BD Pharmingen, #561300) at 1:50 dilution, AF647 Mouse IgG1, κ Isotype Control (BD Pharmingen, #557714) at 1:500 dilution, PE Mouse IgG1, κ Isotype Control (BD Pharmingen, #556650) at 1:1000 dilution. The stainings were performed at RT for 40 min. After a wash in 150 µL FACS buffer with 0.75% saponin and 0.1% Triton X-100 and a wash in 200 µL of FACS buffer with 0.75% saponin, the cells were resuspended in 400 µL of FACS buffer and analysed on FACSVerse Cell Analyzer flow cytometer (BD Biosciences).

### Flow cytometry analysis of cell cycle progression

To label the cells, Click-iT EdU AF 647 Flow Cytometry Assay Kit (ThermoFisher Scientific, #C10424) was used. Cells were fed with respective medium (cE8, hE8 or B8+) containing 10 µM of EdU and incubated in 37 °C for 2 h. Then, cells were washed with PBS and incubated with TryplE™ Express (Thermo Scientific, #12605010): 0.5 mM EDTA in 3:1 proportion at 37 °C for 2 min. Cells were then dissociated in respective cell medium, centrifuged at 100 g for 5 min, resuspended in 200 µL of 1% BSA (Bovogen, #BSAS-NZ) in PBS and transferred to a 96 well plate with a round bottom (Euroclone, #ET3196). Next, cells were centrifuged at 4 °C, 250 g for 5 min, resuspended in 100 µL of eBioscience Fixable Viability Dye eFluor 780 (Invitrogen, #65-0865-14) diluted 1:1000 in 1% BSA/PBS and incubated on ice for 15 min and centrifuged again at 4 °C, 250 g for 5 min. After washing in 200 µL 1% BSA/PBS and centrifugation at 4 °C, 250 g for 5 min, cells were fixed in 40 µL of Click-iT fixative (component D) at RT for 15 min, washed in 200 µL of 1% BSA/PBS and centrifuged at RT, 250 g for 5 min. Then, the pellet was resuspended in 100 µL of 1x saponin-based permeabilization and wash reagent (Component E), incubated at RT for 15 min. 150 µl of Click-iT reaction cocktail was added, mixed well and incubated at RT for 30 min. Then, cells were centrifuged at RT, 250 g for 5 min and washed with 200 µl of component E. The cells were suspended in 200 µL of FxCycle Violet Stain (Molecular Probes, #F10347) diluted 1:1000 in component E and incubated at RT for 30 min in the dark. Without washing, the cells were analysed on FACSVerse Cell Analyzer flow cytometer (BD Biosciences).

### Bulk RNA sequencing

Cells at 70–80% confluency were washed with PBS without calcium and magnesium, then treated with 0.5 mM EDTA in PBS at 37 °C for 2 min. EDTA was aspirated and cells were detached and suspended in DMEM/F12. The cells were then centrifuged at 100 g for 5 min, the supernatant was then aspirated, and the cell pellet was suspended in 350 µL of Lysis Buffer from quick-RNA Mini Prep kit (Zymo Research, #R1055). Total mRNA was extracted with the same RNA extraction kit, following the kit protocol (Instruction Manual Ver. 4.1.6, page 7). Briefly, the lysates were transferred into Spin-Away Filters, centrifuged at RT, 14000 g for 30 s. The flowthrough was combined with 350 µL of pure ethanol (Sigma-Aldrich, #51976-500ML-F), mixed and transferred to Zymo-Spin IIICG columns and centrifuged at RT, 14000 g for 30 s. The columns was washed in 400 µL of RNA Wash Buffer and centrifuged as above, discarding the flow-through. Then, 80 µL of DNase mix consisting of 5 µL of DNase I and 75 µL of DNA Digestion Buffer were added to each column and incubated at RT for 15 min. After adding 400 µL of RNA Prep Buffer and centrifuging as above, the flow-through was discarded. 700 µL of RNA Wash Buffer was added, centrifuging as above and discarding the flow-through. Then, 400 µL of RNA Wash Buffer was added and the columns were centrifuged at RT, 14000 g for 1 min, discarding the flowthrough. The RNA was then eluted by adding 70 µL of nuclease-free water, incubating at RT for 1 min, then centrifuging at RT, 14000 g, 30 s. The concentration and quality of RNA was evaluated with the Tapestation RNA kit (Agilent, #5067-5576 and #5067-5577). For each sample, 500 ng of the RNA was used to prepare the mRNA library, according to the manufacturer instructions (document nr #1000000124518 v04 - https://support-docs.illumina.com/LP/IlluminaStrandedmRNA/Content/LP/Illumina_RNA/Protocol_SM_ST.htm) of the Stranded mRNA Prep Ligation kit (Illumina, #20040534). Kit-specific Illumina UDI indexes (Illumina, #20040553) were used to allow sample multiplexing on the flow cell. Libraries were quantified with Qubit dsDNA high sensitivity assay kit (Invitrogen #Q332301) prior to pooling. 35M reads were allocated to each library equally, they were sequenced on a Nextseq 1000 (Illumina) and they were loaded on a P2 flow cell of 100 cycles (Illumina, #20100987). They were then sequenced single end by cycle allocation of 10 for both indexes and 118 for read 1.

Demultiplexing was performed with bcl2fastq (Illumina, version 1.8.4 - https://emea.support.illumina.com/downloads/bcl2fastq_conversion_software_184.html) to generate fastq files. Fastq files were QC with FastQC and were trimmed using TrimGalore (version v0.6.10 - https://github.com/FelixKrueger/TrimGalore) in single-end mode. The adapter used is “CTGTCTCTTATACACATCT”, and due to the specificities of the library it was necessary to remove 4 additional bases at the 3’ end and 1 base at the 5’ end. Genome indexing was performed using STAR (version 2.7.11b - https://github.com/alexdobin/STAR) in genomeGenerate mode. The gtf file (Homo_sapiens.GRCh38.111.gtf) and the fasta file (Homo_sapiens.GRCh38.dna_sm.primary_assembly.fa) were downloaded from ensembl. Then data were aligned on the indexed human genome using STAR in GeneCounts mode. Raw count data were processed in R (R version 4.3.3) by using Rstudio server (2023.12.1 Build 402 “Ocean storm” release - https://dailies.rstudio.com/version/2023.12.1+402/) within a docker (Ubuntu 24.04) to ensure reproducibility. Firstly, a unique count matrix was created and filtered using dplyr R-package by merging all the nine counts matrices (ReadsPerGenes.out.tab files generated by STAR alignment) and the gene info file (geneInfo.tab file generated by STAR indexing). The final matrix was then filtered by selecting for protein-coding genes and for genes with a minimum of 3 counts in at least one of the nine samples. A metadata table was created with information for each sample on the culture medium and the number of the replicate.

QC analysis was performed on raw data calculating the percentage of mitochondrial counts on the total number of counts in each of the nine samples, then further analyses were performed according to the RNAseqQC pipeline ( https://cran.r-project.org/web/packages/RNAseqQC/vignettes/introduction.html). This pipeline uses the RNAseqQC package and it needs the RNAseq data packed into a Deseq2 object (the DESeqDataSet), which was obtained with the make_dds function by combining the gene counts matrix and the sample information metadata. On the resulting Deseq2 object different analyses were performed: (1) the plot_total_counts function allowed to evaluate the library size; (2) the plot_library_complexity function evaluated the library complexity; (3) the plot_gene_detection function assessed the number of detected genes for each sample; (4) the plot_biotypes function stratified on a graph the total gene counts by their different gene biotypes. According to the pipeline a variance stabilising normalisation was performed using DESeq2 vst function. The obtained output was used for further QC analyses: (1) the plot_chromosome function was used to evaluate differential expression on chromosomal regions for chromosomes 1 to 22, X, Y and mitochondrial chromosome; (2) the plot_sample_MAs to evaluate the variability among the replicates of the same medium. Then data were corrected using the removeBatchEffect function (limma) on the three replicates. Both normalised (vst) counts and normalised (vst) and corrected counts were used for the following analyses: (1) the plot_sample_clustering function generated the Pearson correlation matrix considering the top 1000 most variable genes; (2) the plot_pca_scatters function generated all the possible combinations of PCA plots considering the first nine components; (3) the plot_pca function generated the PCA coordinates and loadings for each component.

Then differential gene expression analysis was performed using limma, glimma and edgeR ^7^. Firstly a design model matrix was created with the model.matrix function (stats) using the information on the culture medium for each sample provided by the meta-data table. The design model was then used to create the contrast matrix with the makeContrasts function specifying the three comparisons taken into consideration, namely B8+ vs hE8, hE8 vs cE8, and cE8 vs B8+. Raw counts were normalised in counts per million (cpm) using the voom function, and then data were corrected with the removeBatchEffect function. Linear model fitting was performed on normalised corrected counts with the lmFit function, the model was then fitted on the contrast matrix with the contrast.fit function, and finally t-statistics was performed with the eBayes function. The same analysis was repeated three times considering different design models: B8+ vs others (hE8 + cE8), cE8 vs others (B8+ + hE8), hE8 vs others (cE8 + B8+), with one contrast coefficient each. Data were normalised starting from the initial raw matrix using TPM normalisation with the NormalizeTPM function (ADImpute). Gene length file was obtained from the gtf file and it is available with the raw data on BioStudies. Log was set to FALSE and scale was set to 1. Normalised data were then corrected with the removeBatchEffect function (limma) on the three replicates R1, R2, and R3.

WGCNA analysis ^8^ was performed on normalised (TPM) and corrected counts. Firstly a set of thresholding power was set as a vector from 1 up to 20, and it was used within the pickSoftThreshold function to calculate the scale free topology fit index for each of the 20 powers. Indices were plotted to pick the power near the curve of the plot. Module detection was then performed with the blockwiseModules function: each of the resulting modules was assigned to a different colour and plotted as a dendrogram with the plotDendroAndColors function. The moduleEigengenes function was then used to extract the first principal component of the expression matrix for each module. The results were plotted in a heatmap with the pheatmap package ( https://rdrr.io/cran/pheatmap/) representing the expression of each sample in each module. Considering the first differential expression analysis, the first (cE8 vs B8+) and third (hE8 vs cE8) coefficients were extracted from the eBayes object with the topTreat function (limma). For each of the considered comparisons, significant genes were selected. A gene was considered significant when the adjusted p-value was less than 0.05. The selected genes were then plotted on a heatmap with the pheatmap package considering for each gene the mean of Z-score of the three replicates for each medium. For each gene it was also reported the colour of the corresponding module.

The three most represented modules among the significant genes were selected and for each of the three modules it was performed a gene ontology analysis on significant genes using the goana function (limma) to detect the overrepresented gene ontology terms among each of the three gene lists. The top 50 biological process (BP) terms were then extracted using the topGO function (topGO). Gene set enrichment analyses were then performed on the second group of differential gene expression analyses which compares each medium to the group of the other two. Analyses were performed using clusterProfiler ^9^. From each group it was extracted a list of genes with the corresponding fold change. Each list was then ranked for decrescent fold change and used as input for the gseGO function. From each of the obtained gseaResults objects it was represented the gene ranking for neuron maturation (GO:0042551), endoderm development (GO:0007492) and mesoderm development (GO: 0007498) using the gseaplot function (enrichplot) in runningScore mode.

The same analysis was then performed on kegg pathways. In this case the gene lists were taken from the first differential expression analysis, considering cE8 vs B8+ (coefficient 1) and hE8 vs cE8 (coefficient 3). In this case only significant genes (adjusted p-value < 0.05) were taken into consideration for each comparison. Firstly, the two lists of genes were converted from ENSEMBL to ENTREZ using the bitr function. Each list was then ranked for decrescent fold change and used as input for the gseKEGG function. The two resulting gseaResult objects were then used into the pathview function. The pathway.id chosen is “hsa04550” (Signalling pathways regulating pluripotency of stem cells). All the R objects created within the analysis can be found in Extended Data 5 ^6^.

### Transgene integration assay

Cells that were ready for passage were detached with 0.5 mM EDTA as described above and counted using the Bright-Line hemacytometer (Sigma Aldrich, #Z359629-1EA). For each medium, 2 × 10 ^5^ cells per well were seeded on three wells of a 6-well plate (Corning, #353046) that were coated with Geltrex at the matrix density of 15.6 µg cm ^-2 ^in their respective medium (cE8, hE8 or B8+) with 2 µM Thiazovivin. The next day, the cells were fed with 1.8 mL per well of their respective medium. This was supplemented with 200 µL of lipofection mix, prepared by combining OptiMEM (Gibco, #31985070) containing 2 µL of Lipofectamine STEM (Invitrogen, #STEM00001), with OptiMEM containing 1 µg of AAV-CAGGS-EGFP (Addgene, #22212, kind gift of Rudolf Jaenisch), 0.5 µg of pZFN-AAVS1_ELD and 0.5 µg of pZFN-AAVS1_KKR (#159297 and #159298, kind gifts of Kosuke Yusa) and incubating the mix at RT for 15 min. Cells were incubated with the lipofection mix for 4 h in the cell incubator, then the medium was replaced with 2 mL of cells’ respective medium. The medium was changed daily. 48 h post lipofection, the cells were fed their respective medium containing 0.5 ng mL ^-1^ Puromycin (Gibco, #A1113803), which was exchanged every day for seven days. On the first two days of puromycin selection, the medium was also supplemented with 2 µM thiazovivin. At the end of selection, all the wells were scanned with Incucyte SX5 Live-Cell Analysis System (Sartorius) with phase contrast and 488 nm channels using the whole well module. For counting colonies, images from 488 nm channels were used. For each condition, an average number of colonies in 3 wells was derived.

### Single cell sorting

Cells were harvested using TryplE:0.5 mM EDTA solution (3:1) and incubated at 37 °C for 3 min. To ensure single cell dissociation, the cell suspension was passed through a 40 µm cell strainer (Pluriselect, #43-10100-40). After centrifugation at 100 g for 5 min, cells were resuspended in sorting buffer: PBS + 1% Penicillin/Streptomycin (Euroclone, #ECB3001D) + 2% FBS + 10 mM HEPES (Fisher Bioreagents, #10756254). Fixable Viability Dye eFluor 780 was added at 1:1000 concentration to the sorting buffer to label dead cells. Live cells were single-sorted on Matrigel-coated (Corning, #356277) 96 well plates (Corning, #353072), coated according to the dilution factor provided by the manufacturer. Plates were prepared with the 3 different media (cE8, hE8 or B8+) supplemented with CEPT cocktail. CEPT cocktail has been prepared with 50 nM Chroman1 (MedChem Express, #HY-15392), 5 µM Emricasan (Selleckchem, #S7775), Polyamine supplement 1:1000 (Sigma-Aldrich, #P8483), 0.7 µM Trans-ISRIB (Tocris, #5284). SH800S Sony cell Sorter with 100 µm Chip and single cells (3 drops) setting have been used for the single cell sorting and cells have been seeded at a maximum speed of 100 events per sec. 72 h after sorting, media were replaced to remove CEPT components and then changed every other day for 1 week. The resulting colonies were stained with Crystal Violet (Sigma, #C6158). For the staining, cells were washed with 200 µL of PBS and fixed with 100 µL of PFA 4% at RT 10 min, washed 2 times with 200 µL of PBS, stained with 100 µL of 0.5% Crystal Violet at RT for 10 min and washed 6 times with 200 µL of deionised water to remove the excess dye. Then, the colonies were counted.

### Directed differentiation in a monolayer towards cardiomyocytes

Cardiomyocytes were differentiated using a previously published protocol ^10^. Briefly, hiPSCs were seeded on the wells of 12-well plate (Corning, #353043), 1.5–3 × 10 ^5 ^cells per well in their respective medium (cE8, hE8 or B8+) with 2 µM thiazovivin. The next day, cells were primed for differentiation with 1 mL of their respective medium containing 1 µM CHIR99021 (Cayman Chemicals, #004CA13122). The day after (day 0 of differentiation), cells were induced towards mesoderm with 2 mL of RBA medium, containing RPMI 1640 (Gibco, #11875093), 0.5 mg mL ^-1^ BSA (Sigma-Aldrich, #A8412-100ML) and 213 µg mL ^-1^ L-ascorbic acid 2-phosphate trisodium salt (Fujifilm, #321-44823), supplemented with 5 μM CHIR99021. Two days later, the differentiation towards cardiac mesoderm was continued by adding 2 mL of RBA supplemented with 2 μM WNT-C59 (Cayman Chemicals, #16644). On day 4, medium was replaced with 2 mL RBA. From day 6, cells were fed with 2 mL of RPMI 1640 supplemented with B27 (Thermo Fisher Scientific, #17504044) every other day.

### Flow cytometry analysis of cardiomyocytes

On the 14th day of differentiation, cardiomyocytes were washed with 1 mL of 0.5 mM EDTA in PBS, then treated with 0.5 mL of 0.25% trypsin (Euroclone #ECB3051D) in 0.5 mM EDTA in 37 °C for up to 10 min. The cells were triturated and 0.5 mL of stop solution (RPMI + 10% FBS) was added. After resuspension, cells were centrifuged at 190 g for 5 min, resuspended in 600 µL of PBS and transferred on a 96-well plate with a round bottom (Euroclone, #ET3196). The cells were centrifuged at 200 g for 5 min, resuspended in 100 µL of Fixable Viability Dye eFluor 450 diluted 1:1000 in PBS and incubated at RT) for 10 min. After three cycles of washes consisting of resuspending cells in 200 µL of FACS buffer and centrifuging at 200g for 5 min, the cells were fixed in 100 µL of 4% PFA at RT for 10 min. Then, the cells were washed three times as above with FACS buffer and permeabilized in 100 µL FACS buffer with 0.75% saponin at RT for 40 min. After centrifuging at 200 g for 5 min, the pellet was resuspended with 50 µL of antibodies diluted in permeabilization buffer. For each sample, TNNT2 staining and respective isotype control staining were performed. In addition, single stained controls were performed on the pooled samples. Antibodies used for the stainings: AF 647 Mouse Anti-Cardiac Troponin T (BD Pharmingen, #565744) at 1:100 dilution, AF 647 Mouse IgG1, κ Isotype Control (BD Pharmingen, #566011) at 1:100 dilution. The stainings were performed at RT for 40 min. After a wash in 150 µL FACS buffer with 0.75% saponin and 0.1% Triton X-100 and a wash in 200 µL of FACS buffer with 0.75% saponin, the cells were resuspended in 400 µL of FACS buffer. Cardiomyocytes were analysed on FACSVerse Cell Analyzer or FACSCelesta Cell Analyzer flow cytometers (both from BD Biosciences).

### Cardiac differentiation in left ventricle organoids

Differentiation towards left ventricle cardiac organoids was performed as published ^11^. Briefly, hiPSCs were dissociated as single cells and seeded 5 × 10 ^4 ^cells per well on a 24-well plate (Greiner, #GR662160) in a respective medium (cE8, hE8 or B8+) with 5 µM ROCK inhibitor (Y-27632 dihydrochloride - Cayman Chemicals, #004CA16644). 24 h after the seeding, the medium was replaced with Mesoderm induction media. After 36 h, the cells were detached with TryplE Express incubation at 37 °C for 2 min, then resuspended in Cardiac Mesoderm Induction medium with 5 µM ROCK inhibitor. The cells were seeded in a low attachment 96-well plate (Thermo Scientific, #15227905) at 1.5 × 10 ^4^ cells per well and centrifuged at 140 g for 4 min. The next day, the cells are fed with Cardiac Mesoderm Induction medium. For the following two days, the medium was changed to Cardiac Mesoderm Induction medium. For the following two days, the medium was changed to Cardiomyocytes Induction medium.

The media were based on CDM that consisted of 0.4% mg mL ^-1^ BSA (Stem Cell Technology, #100-0177) in 50% IMDM (Gibco, #21980065) plus 50% Ham’s F12 Nutrient Mix with GlutaMAX (Gibco, #31765068), supplemented with 1% concentrated Lipids (Gibco, #11905031), 0.004% monothioglycerol (Sigma, #M6145-100ML) and 15 μg mL ^-1^ of transferrin (Optiferrin – Invitria, #777TRF029-1G). The Mesoderm induction media consisted of CDM supplemented with 6 ng mL ^-1^ FGF2-G3 (Qkine, #Qk035), 5 µM LY294002 (Selleckchem #1105), 5 ng mL ^-1^ Activin A (Qkine, #Qk001), 8 ng mL ^-1^ BMP4 (Qkine, #Qk038), and 5 µM CHIR99021 (Cayman Chemicals, #004CA13122). Cardiac Mesoderm Induction medium consisted of CDM supplemented with 8 ng mL ^-1^ BMP4, 1.6 ng mL ^-1 ^FGF2-G3, 10 µg mL ^-1^ Insulin (Thermo Scientific #A11382II), 2 µM Wnt-C59 (Cayman Chemicals, #16644) and 50 nM of retinoic acid (Sigma Aldrich, #R2625). Cardiomyocytes Induction medium consisted of CDM supplemented with 8 ng mL ^-1^ BMP4, 1.6 ng mL ^-1 ^FGF2-G3 and 10 µg mL ^-1^ Insulin.

### Flow cytometry analysis of organoids

After 7.5 days of organoid differentiation, the organoids were pooled together (for each condition, 8 for TNNT2 staining, 8 for isotype staining) and washed two times with 0. 5 mM EDTA in PBS, then incubated with 0.5% trypsin in 0.5 mM EDTA in 37 °C for 15 min, triturated every 5 min. After adding a stop solution, the cells were centrifuged at 4 °C, 200 g for 5 min. The pellet was resuspended in eFluor 450 viability dye, diluted 1:1000 in 1% BSA in PBS and incubated on ice for 18 min. After centrifugation at 4 °C, 240 g for 5 min, the cells were fixed in 4% PFA at RT for 15 min, washed in 1% BSA/PBS and centrifuged at 4 °C, 240 g for 5 min. Then, the cells were suspended in a permeabilization buffer (0.75% saponin in FACS buffer), centrifuged at RT, 240 g for 5 min, resuspended in antibodies diluted in permeabilization buffer and incubated at RT for 40 min. Antibodies used for the stainings: AF 647 Mouse Anti-Cardiac Troponin T (BD Pharmingen, #565744) at 1:100 dilution, AF 647 Mouse IgG1, κ Isotype Control (BD Pharmingen, #566011) at 1:100 dilution. After a wash with permeabilization buffer, the cells were resuspended in 1% BSA/PBS and analysed on the FACSVerse Cell Analyzer flow cytometer.

### Data analysis

Flow cytometry data was analysed on FlowJo 10 (BD Biosciences - https://www.flowjo.com/solutions/flowjo - alternatives in the Software availability section), using single stained pooled samples to set up a compensation matrix. Doublets were excluded by comparing height to area both in forward and side scatter (FSC and SSC). The gates for live cells were determined by comparing unstained cells with viability dye only cells. The gates for target protein - positive cells were set up based on their respective isotype controls.

Graphs and statistics were generated using Prism 10 (GraphPad - https://www.graphpad.com - alternatives in the Software availability section). Unless specified otherwise, the bars represent the median value. When two independent adaptations to the media were used, the experiment was repeated two times and for each adaptation, the average value from both replicates was used for the analyses. For statistical analyses of RNA-seq data ( Figure 3), limma and clusterProfiler were calculating and adjusting the p-values at the level of all analysed genes.

## Results

### Refinement of TGF-β and FGF2 signalling sources and dosage for normoxic hiPSC culture

**Figure 1.**
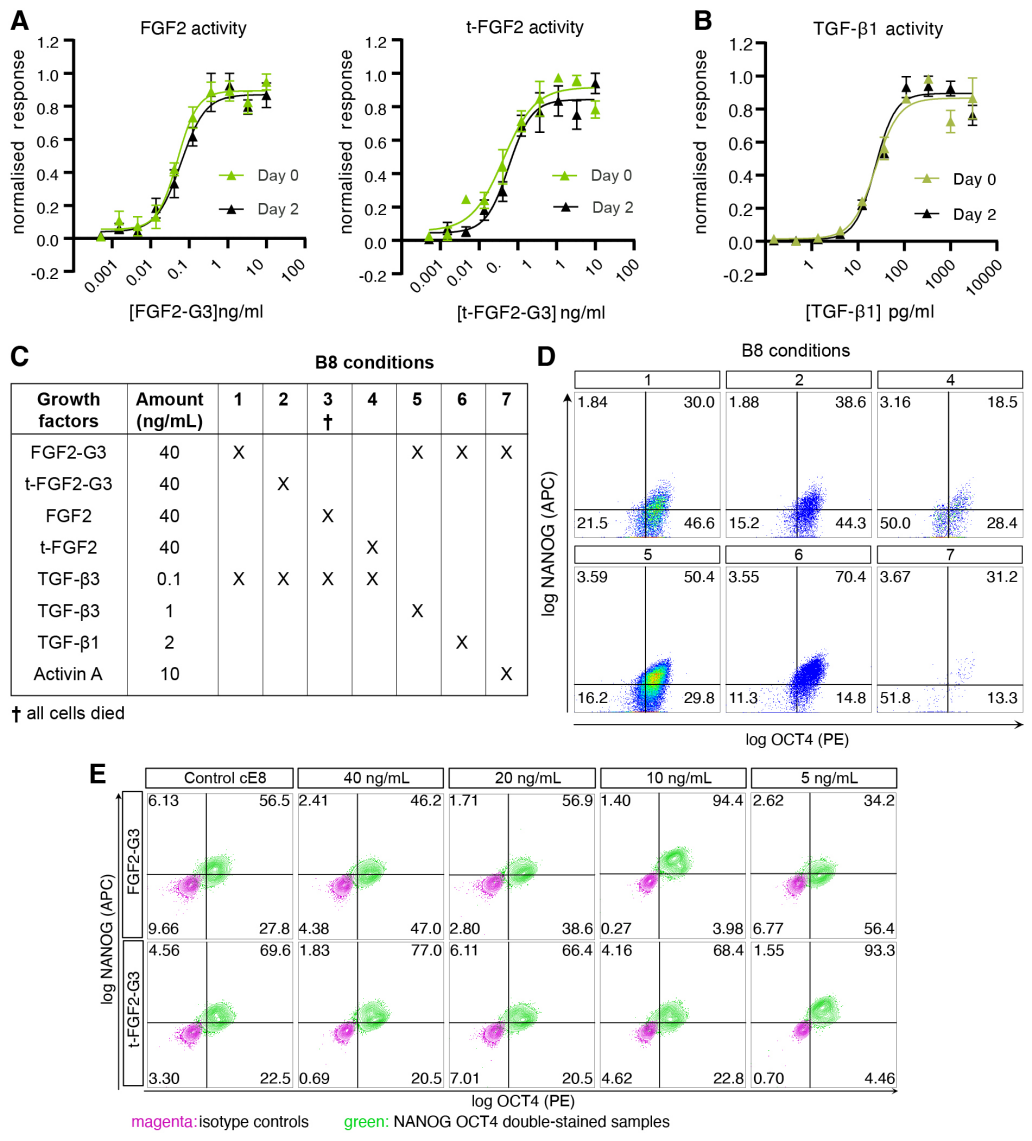
Evaluation of TGF-β and FGF2 sources and concentrations in B8 media. ( **A**– **B**) Variants of FGF2-G3 ( **A**) or TGF-β ( **B**) activity assay using a serum response element luciferase reporter assay in transfected HEK293T cells. Medium pre-incubated in 37 °C for 2 days was compared to the fresh medium. Firefly luciferase activity was normalised to control Renilla luciferase activity. n = 3 experiments. ( **C**) Schematic of B8-based media formulations with different types of FGF2 (standard *versus* thermostable [G3]; full length *versus* truncated [t]) and TGF-β-superfamily growth factor. ( **D**– **E**) Representative flow cytometry data of OCT4 and NANOG expression in hiPSCs grown in media described in panel **C** ( **D**; no data could be acquired for condition 3), or in media with 1 ng/mL TGF-β3 and the indicated concentrations of the truncated or full-length FGF2-G3 ( **E**).

The maintenance of primed pluripotency in hPSCs relies on the combination of signalling through the Activin/Nodal/TGF-β-SMAD2/3 and FGF2-MAPK pathways ^12, 13^. We produced these growth factors using an animal origin-free microbial ( *E.coli*) expression system and without the use of protein tags, as these are not suitable for large-scale manufacture methods, and validated their thermostability ( Figure 1A,B). Besides the full-length FGF2-G3 (154 amino acids, used in the published B8 formulation), we tested a truncated version (t-FGF2, 145 amino acids) that proved marginally more potent at lower doses ( Figure 1A). Both factors retained their activity after 2 days of preincubation in conditioned media at 37 °C, suggesting that they are suitable for weekend-free media formulations. Distinctly from unmodified FGF2, TGF-β molecules are not thought to be unstable at 37 °C; we confirmed this to be the case for TGF-β1, as an exemplary molecule from this family ( Figure 1B).

We employed these growth factors to compose various media formulations inspired by B8 ^4^, which we tested by growing a hiPSC line obtained from a healthy male donor, WTC11 ^14^, in normoxic conditions without the daily medium change (weekend-free). As expected, the use of non-thermostable FGF2 or t-FGF2 resulted in pluripotency loss and eventual cell death ( Figure 1C). Surprisingly, however, neither FGF2-G3 nor t-FGF2-G3 supported pluripotency when used in combinations with TGF-β3 at the published concentration of 0.1 ng mL ^-1^ ( Figure 1C,D). As the morphology of hiPSCs cultured in these conditions resembled that of neural rosettes, we speculated that pluripotency loss may result from an excessively low dosage of TGF-β3. Indeed, reduced SMAD2/3 signalling is known to rapidly drive commitment to neuroectoderm through epigenetic and epitranscriptional mechanisms that maintain NANOG expression ^15– 17^. We thus optimised both the source and the concentration of growth factors upstream of SMAD2/3 ( Figure 1C,D). Increasing the concentration of TGFβ-3 by 10-fold (1 ng mL ^-1^) improved the fraction of cells that were double positive for pluripotency markers NANOG and POU5F1 (also known as OCT4). Substituting TGF-β3 with TGF-β1 at a 2-fold higher dose (2 ng mL ^-1^, the concentration reported in the E8 formulation), led to an even greater fraction of NANOG ^+^ OCT4 ^+^ hiPSCs. Activin A, supplemented at 10 ng mL ^-1^ as per the chemically defined media (CDM) formulation ^13^, proved unsuitable in B8-like media.

To improve pluripotency maintenance in B8-like media based on TGF-β3, we then focused on the type and concentration of FGF2-G3. We hypothesised that tag-free FGF2-G3 variants may be too potent when used at the reported concentration of 40 ng mL ^-1^, and tested lower amounts, reducing the concentration down to 5 ng mL ^-1^ for both FGF2-G3 and t-FGF2-G3. ( Figure 1E). Remarkably, 10 ng mL ^-1^ FGF2-G3 and 5 ng mL ^-1^ t-FGF2-G3 proved optimal for pluripotency maintenance, achieving >90% NANOG ^+^ OCT4 ^+^ ( Figure 1E). We elected to use t-FGF2-G3, which reduces the cost of FGF2 sourcing by 2-fold, and named the resulting formulation B8+, to distinguish it from the original B8 (See Figure 1).

### B8+ supports hiPSC proliferation and self-renewal

**Figure 2.**
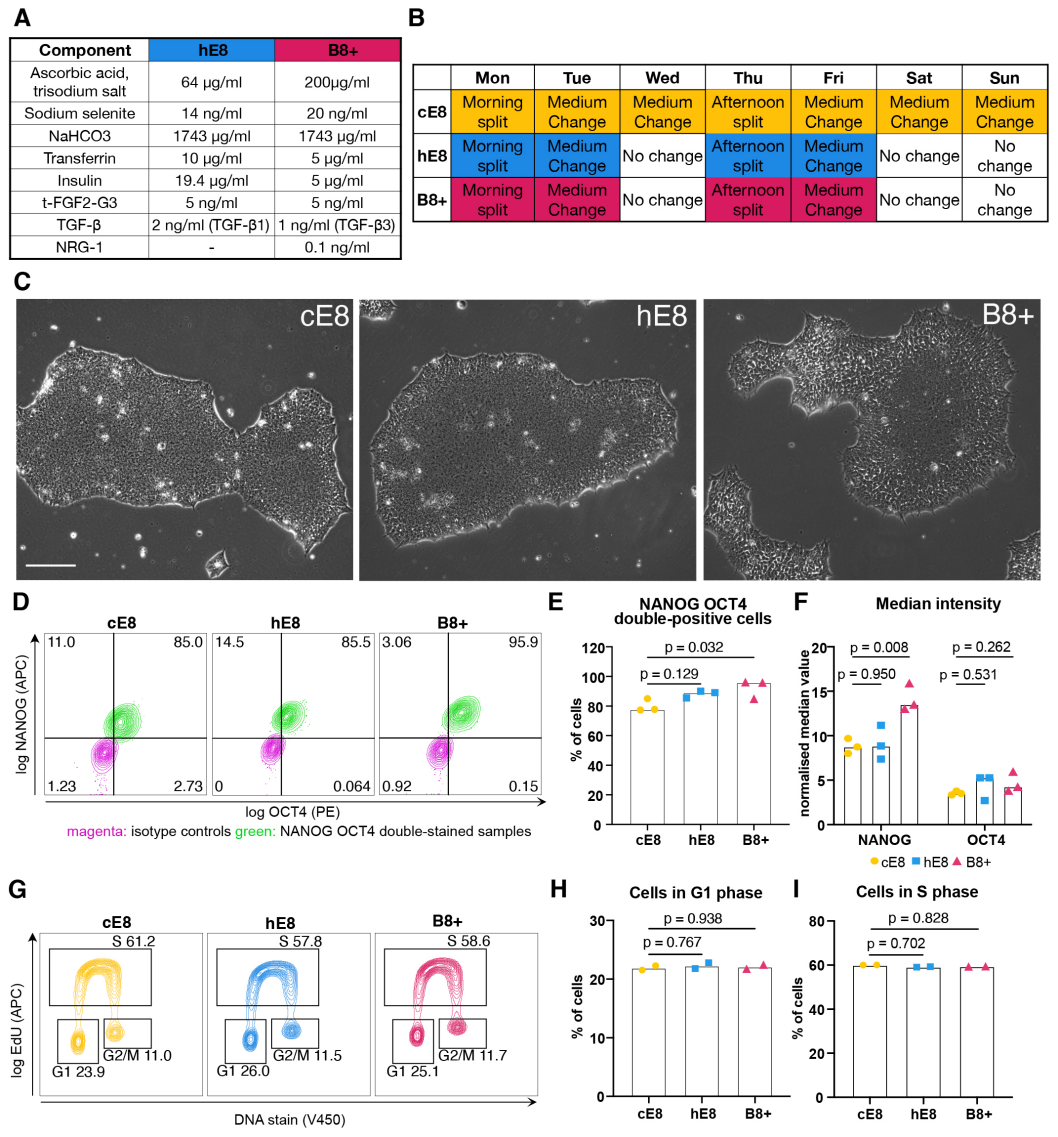
Characterization of hiPSCs adapted to weekend-free media. ( **A**) Final concentrations of components in the supplements for two weekend-free media compositions (both based on DMEM/F12). ( **B**) Weekly schedule of hiPSC culture in three different media. Days highlighted in colour involve media changes. ( **C**) Phase-contrast images of representative hiPSC colonies 72 h after passage. Scale bar: 200 µm. ( **D**) Representative flow cytometry analyses of OCT4 and NANOG expression in hiPSCs grown in different media. ( **E**– **F**) Flow cytometry quantifications of the percentage of NANOG ^+^ OCT4 ^+^ cells ( **E**) and the normalised median fluorescence intensity value of NANOG and OCT4 ( **F**). Median values were normalised through division by the median intensity value of the respective isotype control. n = 3 independent adaptations of the cells into the media. Statistical analyses by one-way ANOVA followed by Dunnet’s multiple comparisons test. ( **G**) Representative flow cytometry cell cycle analyses in EdU-treated cells stained for DNA content. ( **H**– **I**) Flow cytometry quantifications of the percentage of cells in G1 ( **H**) or S ( **I**) phase. n = 2 independent adaptations of the cells into the media. Statistical analyses by one-way ANOVA followed by Dunnet’s multiple comparisons test.

Having developed a promising media formulation, we set out to comparatively assess it in various applications routinely performed by our group. Besides B8+, we designed a homemade E8-based formulation (hE8) by substituting FGF2 at 100 ng mL ^-1^ with t-FGF2-G3 at 5 ng mL ^-1^ (to enable weekend-free culture and reduce costs) and by equalising the NaHCO _3_ concentration in hE8 and B8+ (to allow a more rigorous comparison; Figure 2A). We adapted WTC-11 hiPSCs to B8+ and hE8 culture according to a weekend-free schedule for at least five passages and compared them to passage-matched hiPSCs cultured in commercial E8 (cE8) grown according to the manufacturer's recommended protocol with daily media changes ( Figure 2B).

First of all we examined hiPSC morphology, which proved different. While cells grown in cE8 formed compact colonies 3 days after seeding in small clumps, cells grown in both weekend-free media occupied more space (Extended Data 1 ^6^). This was more evident in B8+, where the colonies were the least compact ( Figure 2C). This morphology is similar to the one described for the original B8 formulation ^4^.

To verify whether this shift in morphology reflects a change in pluripotency, we analysed the levels of NANOG and OCT4 expression by flow cytometry ( Figure 2D). We observed a high fraction of NANOG ^+^ OCT4 ^+^ double positive cells in all the media, with B8+ supporting the highest proportion of pluripotent cells ( Figure 2E). In addition, cells grown in B8+ exhibited increased levels of NANOG expression ( Figure 2F).

To assess whether cell proliferation is affected, we investigated how many cells are in G1 phase, which is very short in iPSCs ^18^. Similar proportions of cells were in G1 phase for all the media ( Figure 2G,H), as well as in S phase ( Figure 2G,I), suggesting the similar division rate in all the media. In all, we concluded that both weekend-free media formulations effectively support hPSC pluripotency (See Figure 2).

### B8+ primes cells for mesendoderm differentiation

**Figure 3.**
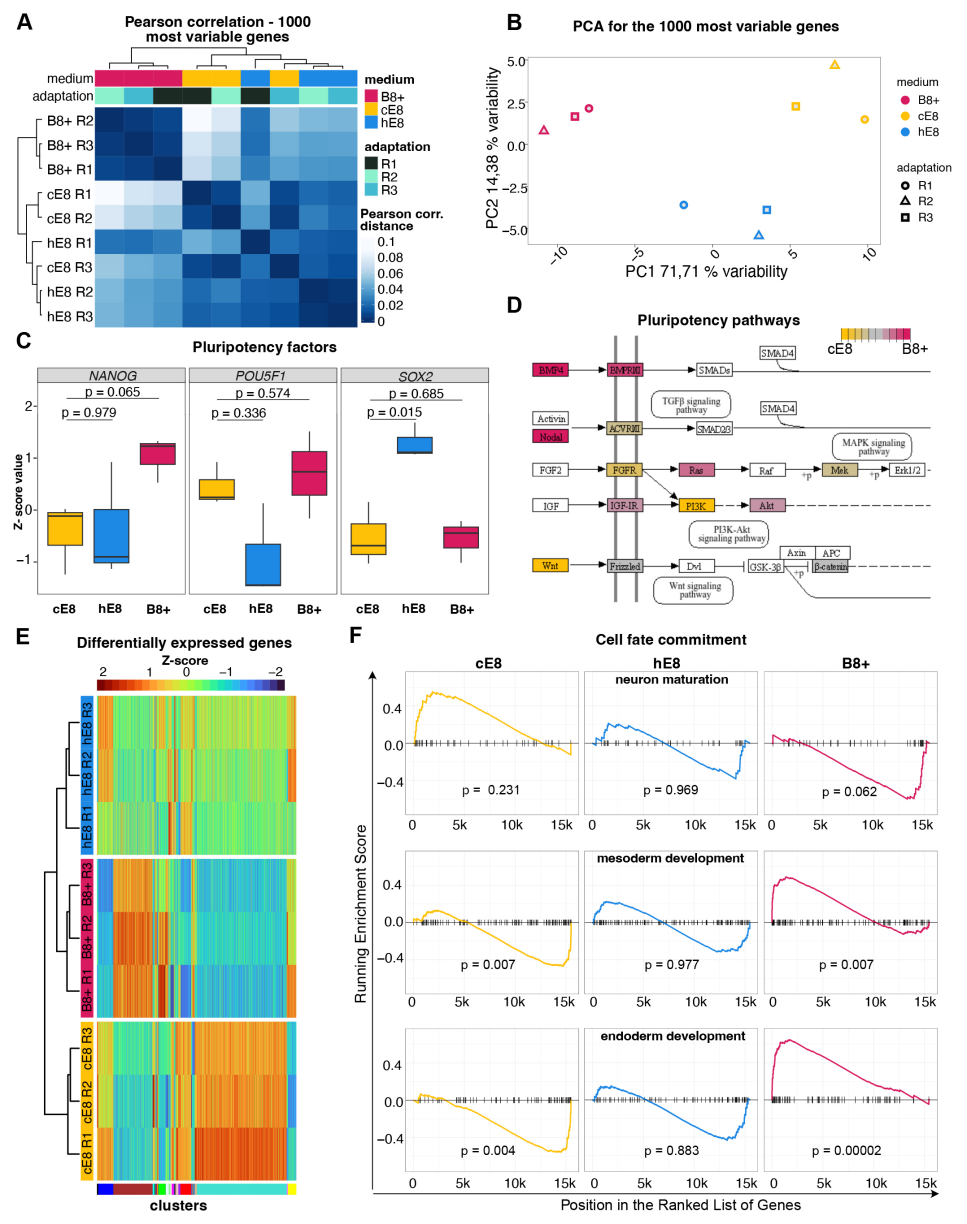
Effects of weekend-free media on gene expression. ( **A**– **B**) Pearson correlation ( **A**) and Principal Component Analysis (PCA; **B**) based on the expression of the 1000 most variable genes in bulk RNA-seq measurements of hiPSCs adapted to the indicated media. n =3 independent adaptations of the cells into the media. ( **C**) Changes in gene expression of key pluripotency genes, measured as a Z-scores of TPM normalised counts. P-values are calculated by linear model fitting and are adjusted for Benjamini-Hochberg false discovery rate. ( **D**) Schematic of genes involved in primed pluripotency signalling (according to KEGG pathways); those significantly upregulated in cE8 or B8+ are coloured in shades of yellow or red, respectively. ( **E**) Heatmap of differentially expressed genes. Genes were clustered into WGCNA modules based on the TPM normalised counts of all the replicates in a particular medium. ( **F**) Gene Set Enrichment Analysis (GSEA) using gene signatures of germ layers differentiation. For each medium, genes were ranked in descending order of the fold change of gene expression level in comparison to other media. Enrichment was tested based on hypergeometric distribution and adjusted for multiple testing. The coloured lines visualise the enrichment value.

To get additional insight into the transcriptional state of the cells growing in different media, we performed bulk RNA-sequencing. Pearson correlations and Principal component analysis (PCA) indicated that independent adaptations to B8+ and hE8 led to reproducible changes in gene expression compared to cE8 ( Figure 3A,B). As anticipated, cells grown in hE8 are more similar to those in cE8, while the gene expression profile of cells cultured in B8+ is more different from the other two conditions.

In line with flow cytometry analyses, the expression *NANOG* was highest in B8+ ( Figure 3C). Both weekend-free media showed similar levels of *POU5F1/OCT4*, and hE8 expressed higher level of *SOX2* compared to cE8 ( Figure 3C). Analysis of pluripotency KEGG pathways revealed that autocrine Nodal and BMP4 were upregulated in B8+ compared to cE8, while WNT ligands were downregulated ( Figure 3D).

Differential gene expression analyses revealed 117 genes differentially expressed in B8+ *versus* cE8 (53 up- and 64 down-regulated), while only 24 genes were differentially expressed in hE8 versus cE8 (10 up- and 14 down-regulated, all filtered for adj. p-value < 0.05 and an absolute log _2_ fold change of 2; Extended Data 2 ^6^). We selected genes specifically up- or down-regulated only in B8+, hE8, or cE8, and clustered the union of these gene lists based on their gene expression across all samples. This led to the identification of 54 modules, two of which were overrepresented and contained genes specifically upregulated in B8+ or in cE8 ( Figure 3E). Gene ontology (GO) analyses indicated an enrichment of terms involved in mesoderm and endoderm development in the gene module upregulated in B8+, and enrichment of terms involved in neuron development in the gene module upregulated in cE8 (Extended Data 3 ^6^). In line with these findings, gene set enrichment analyses (GSEA) indicated that cells grown in B8+ are enriched for genes characteristic for endoderm and mesoderm, and depleted of genes typical of neurons ( Figure 3F). The opposite was observed for cells grown in cE8, while hE8 was not associated with significant changes in this analysis ( Figure 3F). Overall, these results suggest that B8+ primes hiPSCs towards meso- or endodermal differentiation (See Figure 3).

### B8+ supports hiPSC genome editing, single cell cloning, and cardiac differentiation in 2D and 3D

**Figure 4.**
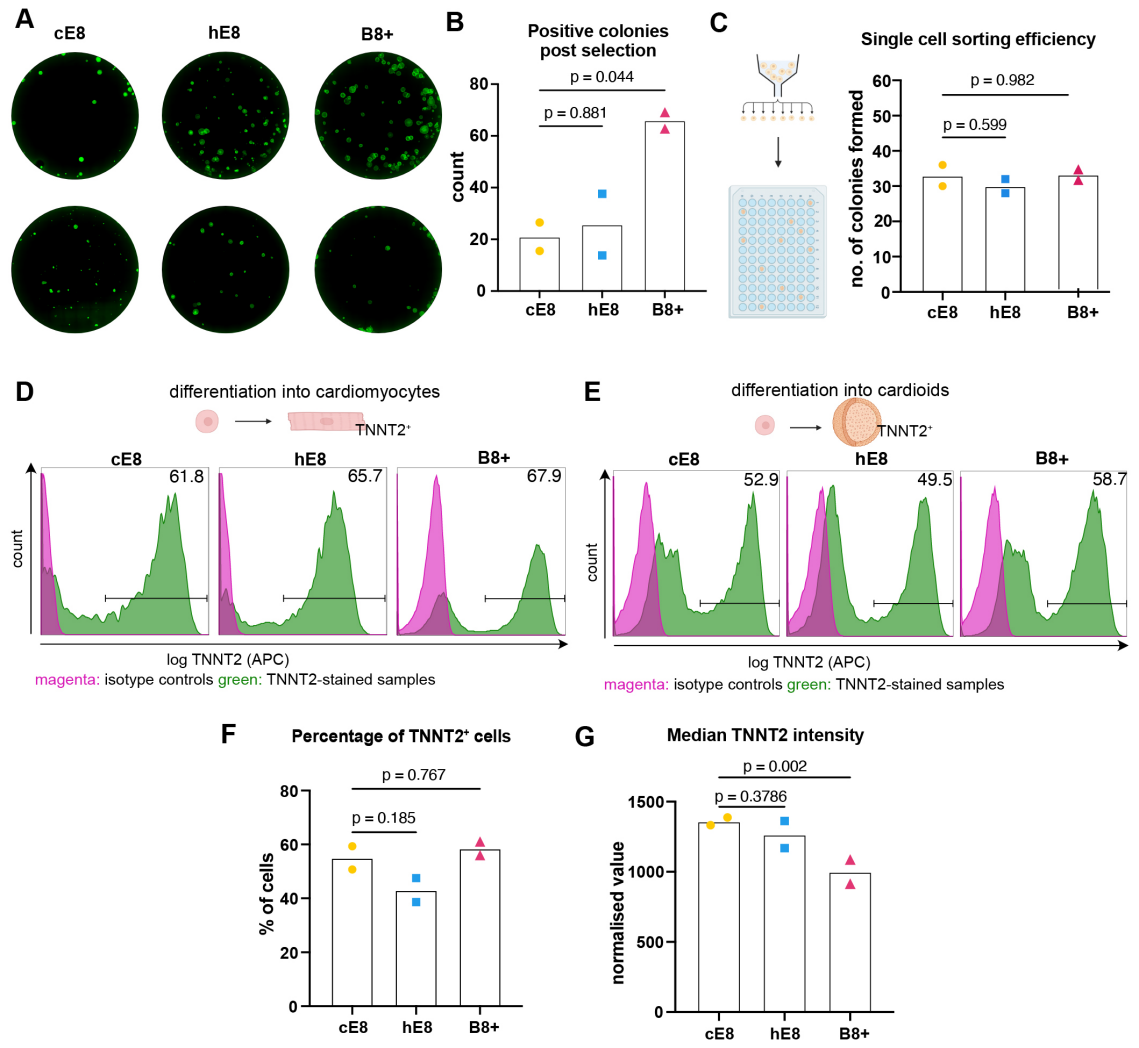
Applications of hiPSCs adapted to weekend-free media. ( **A**) Representative images of GFP-positive iPSC colonies after puromycin selection of *AAVS1* genome-edited cells. ( **B**) Quantification of the positive colonies. n = 2 media adaptations. ( **C**) Quantification of hiPSC clonality after single cell sorting into 96-well plate. n = 2 media adaptations. ( **D**– **E**) Representative flow cytometry analyses of TNNT2 expression in hiPSCs differentiated towards cardiomyocytes in monolayer ( **D**) or organoids ( **E**). ( **F**– **G**) Flow cytometry quantifications of the percentage of TNNT2-positive cells ( **F**) and the normalised median TNNT2 value ( **G**) in cardiac organoid differentiations. Median values were normalised through division by the median intensity value of the respective isotype control. n = 2 media adaptation. All statistical analyses of the data in this figure were performed by one-way ANOVA followed by Dunnet’s multiple comparisons.

Next, we verified whether the iPSCs grown in the weekend-free media are suitable for typical applications. First, we examined their ability to undergo genome editing. For this, we co-transfected hiPSCs with plasmids encoding zinc finger nuclease (ZFN) against the *AAVS1* genomic safe harbour and a targeting vector with homology regions to such locus and carrying both a constitutive EGFP and a gene trap-based puromycin resistance gene ^19^. We then selected with puromycin hiPSCs which had undergone site-specific integration via homology-directed repair and validated the result through fluorescence microscopy for EGFP. These experiments indicated that hiPSCs gave rise to a similar number of genome-edited colonies in hE8 and cE8, while a larger number was obtained in B8+ ( Figure 4A,B).

Encouraged by these results, we also tested the survival and growth of individual hiPSCs following fluorescence activated single-cell sorting), a procedure commonly used to generate single cell clones. To this end, we single-sorted cells into 96-well plates and counted the number of colonies formed after a week of culture. All conditions resulted in ~30% clonality, ( Figure 4C), indicating that hiPSCs tolerate this stressful procedure equally well in weekend-free media compared to cE8.

Lastly, we tested whether the ability of cells to differentiate is affected. To this end, we performed directed differentiation towards cardiomyocytes. While the initial seeding density had to be adjusted for each medium, we could ultimately achieve a high differentiation rate for cells cultured in all conditions ( Figure 4D), suggesting that all media can support the ability of hiPSCs to differentiate into mesodermal derivatives. We investigated further the potential of hiPSCs to differentiate in more complex, three-dimensional organoids, using cardiac organoids as an example. hiPSCs cultured in all media were able to form the organoids, and they differentiated into TNNT2+ cardiomyocytes in a similar proportion ( Figure 4E,F). However, we observed marginally lower levels of expression of cardiac troponin in cells grown in B8+ ( Figure 4G). Overall, these experiments indicate that hiPSCs can be grown in homemade weekend-free media and be used in a variety of applications, which provides an attractive alternative to commercial media (See Figure 4).

## Discussion

To unlock the full potential of hiPSC-based research, their culture, genome editing, and differentiation need to be made more accessible by reducing the workload and financial burden on laboratories. Weekend-free media, such as B8, offer such possibility without compromising cell growth. Production of functional and endotoxin-free FGF2-G3 is complex for individual academic laboratories and requires a licence for the patent. Moreover, in our hands applying the direct formulation of B8 using commercially-available reagents resulted in loss of pluripotency. To overcome this issue, we developed the B8+ formulation, which relies on a 10-fold higher concentration of TGF-β3 but allows an 8-fold lower concentration of FGF2-G3, which largely offsets the cost increase.

Our suboptimal results with B8 may be caused by differences in cell culture conditions. Most importantly, B8 was validated using hPSCs cultured in hypoxic conditions (5% O _2, _5% CO _2_), while our experiments were performed in standard normoxia incubators (5% CO _2_). We speculate that lower doses of TGF-β3 are tolerated in hypoxia, which is more optimal for maintaining pluripotency ^20^. On the other hand, most laboratories do not have access to an hypoxic incubator, and thus normoxic culture of iPSCs is widely used. In the future it would be interesting to rigorously compare these two conditions in B8 and B8+, and investigate the mechanisms affected by these variables. Of note, we cannot rule out that WTC-11 hiPSCs are particularly sensitive to TGF-β levels, but we deem it unlikely since they are widely used and have been cultured in a variety of commercial media without any issue. We eagerly anticipate tests of B8 versus B8+ in other hiPSC lines and by other laboratories in order to gauge the reproducibility of our findings.

The reduced requirements for FGF2-G3 in our B8+ formulation could be partially due to the absence of the 6xHis tag, though a previous report indicated that this would not interfere with FGF2 activity ^21^. Alternatively, employing a rigorous manufacturing process along with stringent quality control procedures ensures high purity and homogeneity in the produced growth factor, resulting in a higher fraction of bioactive FGF2-G3. Lastly, we could reduce the concentration of FGF2-G3 by an additional 2-fold by employing a truncated version comprising the core structured region (145 aa) sufficient for full biological activity.

Preparing B8+ media from commercially-sourced growth factors does not maximise reagent cost savings, but it improves culture reproducibility, within and across laboratories, and therefore saves labour costs and time. In our laboratory we also opted to use a liquid formulation of DMEM/F12 as the basis for B8+, since the batches of powder media resulted in liquid media with varying pH and osmolarity that, despite our adjustment, led to inconsistent cell growth. This seems to be an issue also experienced by others ^22^. With these modifications, the cost of the medium is ~80€/L of B8+ and ~100€/L of hE8, compared to ~650€/L for cE8 (all assuming purchases at current list prices). In addition, weekend-free media use only 57% of the volume that the daily-change medium uses during a week of culture, which lowers the effective costs even more.

hiPSCs grown in weekend free media are in many aspects similar to those grown in daily-changed cE8. In fact, hE8 was not significantly different from cE8 in any of the assays we performed, except for minor differences by sensitive RNA-seq, suggesting that it is a safe and simple alternative to commercial media that does not require substantial adaptation.

On the other hand, cells grown in B8+ show clear changes in cell and colony morphology. This may be related to their apparent priming into a mesendoderm lineage. Despite this, B8+-cultured hiPSCs remain pluripotent for at least 10 passages (we did not test this further). Altogether, the higher expression of mesendoderm markers and lower expression of neuroectodermal ones in this condition may reflect the regionalisation of the post-implantation epiblast, whereby the posterior side gives rise to the primitive streak and the anterior side to neural and ectodermal lineages. This concept is not new, as it was previously demonstrated that region specific PSCs (rsPSCs) can be captured under defined culture conditions ^23^. It is, however, interesting that B8+, which stimulates the same signalling pathways as hE8, results in such different apparent regionalisation. This may result from TGF-β-independent activities of TGF-β3 versus TGF-β1, varying potency and/or stability of these growth factor, or the slightly different levels of these and other molecules (i.e., ascorbic acid, transferrin, insulin, and NRG1).

One of the potential advantages for growing cells in B8+ is a higher efficiency of genome editing. We speculate that this is primarily linked to morphology changes, as less compact cells have better exposure to the transfection reagents. Indeed, we observed a higher efficiency of transient transfection, correlating with a higher number of genome-edited cells. B8+ can thus be a very attractive alternative to commercial media for large-scale genome editing experiments. Nevertheless, it remains to be determined whether the transcriptional differences observed in B8+ result from changes in chromatin modifications and structure that could affect downstream differentiation or other processes. Most notably, differentiation into neural lineages may require adjustments to overcome the mesendoderm priming.

## Ethics and consent

Ethical approval and consent were not required.

## Data Availability

Zenodo: Underlying and Extended Data for Refined Home-Brew Media for Cost-Effective, Weekend-Free hiPSC Culture and Genetic Engineering. DOI: https://zenodo.org/doi/10.5281/zenodo.12684584 ^6^ This project contains the following data: Extended Data 1.zip - Images of hiPSCs adapted to cE8, hE8 and B8+ taken 24, 48 and 72 hours after passage. Contains raw .tiff files for each image and a .pdf with a compiled figure Extended Data 2.zip - Fold change and adjusted p-values from the statistical analyses of bulk RNA-seq data of the hiPSCs grown in cE8, hE8 and B8+ Extended Data 3.zip - Biological Process Gene Ontology terms for genes upregulated in hiPSCs grown in B8+ and cE8 Extended Data 4.zip - protocol for preparation of the supplement for hE8 and B8+ media Extended Data 5.zip - Gene counts, supporting files, and output results of bulk RNA-seq analyses Manuscript Data.zip – Raw data underlying the Figures 1, 2 and 4. Bulk_RNA_seq_archive – archived source code used for generating results in Figure 3 Data are available under the terms of Creative Commons BY 4.0 licence. Uniprot: Data entry for human FGF2. Accession number P09038; https://www.uniprot.org/uniprotkb/P09038/entry ^24^ Uniprot: Data entry for human Activin A. Accession number P08476; https://www.uniprot.org/uniprotkb/P08476/entry ^25^ Uniprot: Data entry for human TGF-b1. Accession number P01137; https://www.uniprot.org/uniprotkb/P01137/entry ^26^ Uniprot: Data entry for human TGF-b3. Accession number P10600; https://www.uniprot.org/uniprotkb/P10600/entry ^27^ Uniprot: Data entry for human NRG1. Accession number Q02297; https://www.uniprot.org/uniprotkb/Q02297/entry ^28^ Biostudies: RNA-seq data generated in the course of this work (raw data underlying Figure 3). Accession number E-MTAB-14237; https://www.ebi.ac.uk/biostudies/arrayexpress/studies/E-MTAB-14237 ^29^

## Software availability

The docker image in the dockerhub repository that contains all the software for the analysis shown in Figure 3 (‘Bulk RNA sequencing’ section of Methods) is available from: https://hub.docker.com/r/hedgelab/rstudio-hedgelab/tags, pulling the image with ‘media_bulk’ tag. All the software included in the image is free.

The source code used for analysis of data presented in Figure 3 is available from: https://github.com/alessandro-bertero/BulkRNAseq_media

Archive source code available from: https://zenodo.org/doi/10.5281/zenodo.12684584 ^6^

License: CC BY 4.0

GraphPad Prism 10 ( https://www.graphpad.com), the software used for statistical analysis, is a proprietary software. Free and open-source alternatives are available, such as SOFA Statistics Open For All ( https://www.sofastatistics.com/home.php) or JASP ( https://jasp-stats.org).

FlowJo 10 ( https://www.flowjo.com/solutions/flowjo), the software used for the analysis of flow cytometry data, is a proprietary software. Free alternatives are available, such as Floerada online tool ( https://floreada.io) or FCSalyzer ( https://sourceforge.net/projects/fcsalyzer/).
