# Refined and benchmarked homemade media for cost-effective, weekend-free human pluripotent stem cell culture

**DOI:** 10.12688/openreseurope.18245.2

**Published:** 2025-09-19

**Authors:** Lukasz Truszkowski, Sveva Bottini, Sara Bianchi, Mirko G. Scrivano, Giulia Ferrari Ramondo, Linda Bellucci, Helen Bell, Silvia Becca, Kirsten E. Snijders, Giulia Savorè, Federica Sozza, Irene Ricca, Cristina Rubinetto, Luana Ferrara, Francesco Neri, Andrea Ditadi, Salvatore Oliviero, Elisa Balmas, Catherine Elton, Alessandro Bertero

**Affiliations:** 1 Department of Molecular Biotechnology and Health Sciences, Molecular Biotechnology Center “Guido Tarone”, University of Turin, Torino, 10126, Italy; 2 Department of Life Sciences and Systems Biology, Molecular Biotechnology Center "Guido Tarone",, University of Turin, Torino, 10126, Italy; 3 San Raffaele Telethon Institute for Gene Therapy, IRCCS San Raffaele Scientific Institute, Milan, 20132, Italy; 4 Qkine, Cambridge, UK; 5 Department of Molecular Biotechnology and Health Sciences, Molecular Biotechnology Center "Guido Tarone", University of Turin, Torino, 10126, Italy

**Keywords:** hiPSC; pluripotency; culture media; thermostable FGF2; TGF beta

## Abstract

**Background:**

Cost-effective, practical, and reproducible culture of human pluripotent stem cells (hPSCs) is required for basic and translational research. Basal 8 (B8) has emerged as a cost-effective solution for weekend-free and chemically-defined hPSC culture. However, the requirement to home-produce some recombinant growth factors for B8 can hinder access and reproducibility. Moreover, we found the published B8 formulation suboptimal in widely-used normoxic hPSC culture. Lastly, the performance of B8 in functional applications such as genome editing or organoid differentiation required systematic evaluation.

**Methods:**

We formulated B8 with commercially available, growth factors and adjusted its composition to support normoxic culture of WTC11 human induced pluripotent stem cell line. We compared this formulation (B8+) with commercial Essential 8 (cE8) and a home-made, weekend-free E8 formulation (hE8). We measured pluripotency marker expression and cell cycle by flow cytometry, and investigated the transcriptional profiles by bulk and single-cell RNA sequencing. We further assessed genomic stability, genome editing efficiency, single-cell cloning, and differentiation in both monolayer and organoids. Finally, we validated key findings using male (H1) and female (H9) human embryonic stem cells.

**Results:**

hE8 performed comparably to cE8 across most functional assays and cell lines. In contrast, cells in B8+ displayed higher NANOG expression and improved genome editing efficiency. At the same time, B8+ led to gene expression changes indicative of marked lineage priming, reflected in altered morphology and differential response to some differentiation protocols. Both weekend-free media resulted in a modest transcriptional shift towards a less metabolically active state, consistent with intermittent media starvation.

**Conclusions:**

Homemade weekend-free media can provide a cost-effective alternative to commercial formulations. hE8, integrating some features of B8 while resembling cE8, emerges as a robust and practical option with limited compromises. B8+, though advantageous in some contexts, warrants caution due to lineage priming effects that may impact differentiation outcomes.

## Introduction

Human pluripotent stem cells (hPSCs, either embryo-derived, hESCs, or induced via reprogramming, hiPSCs) have become a prominent tool in biomedical research. Their ability to differentiate towards virtually all somatic cell types offers the unprecedented ability to study rare, transient, and/or difficult to access cell types, such as those of the developing embryo. hiPSCs can be readily derived from patients, genome-edited to obtain isogenic controls, and employed for disease modelling and drug screening studies, paving the way towards personalised medicine ^1^.

To achieve safe and reproducible hiPSC culture, formulations for feeder-free, chemically defined media such as mTESR ^2^ or Essential 8 ^3^ have been established. However, commercial sources of these media cost hundreds of euros per litre and require daily media changes. Commercial weekend-free formulations have been developed, but their cost is even higher. In all, culturing hPSCs, especially on a large scale, can consume a lot of manpower and financial resources. This puts a strong burden on stem cell labs and represents an important roadblock to the adoption of hPSC technology in non-expert groups.

Basal 8 (B8) is a recently reported, weekend-free medium formulation with reduced amounts of growth factors and other media components to limit medium cost ^4, 5^. The most effective cost-cutting strategy is the in-house production of thermostable FGF2 (FGF2-G3). However, endotoxin contamination and batch-to-batch variation in bacterial FGF2-G3 production can be a challenge for smaller labs. In addition, some applications of B8, such as genome editing or organoid differentiation, remain to be assessed.

In this work, we benchmarked homemade weekend-free media based on commercially available, animal-free growth factors. We focused on two formulations: a modified version of Basal 8 (B8+) and a homemade variant of Essential 8 (hE8), and compared their performance to commercial E8 (cE8) across multiple assays and stem cell lines. This systematic evaluation highlights both the potential and the limitations of homemade weekend-free media as cost-effective alternative to commercial formulations.

## Methods

### hiPSC culture

In all experiments (unless specified otherwise), hiPSCs from the WTC-11 line (RRID: CVCL_Y803), kindly provided by Bruce Conkin (J. David Gladstone Institutes), were cultured feeder-free on dishes coated with Geltrex™ (Gibco, #A1413302) at matrix density of 5.2 µg cm ^-2^ and maintained in normoxic conditions in the CellXpert C170i copper incubator (Eppendorf) at 37 °C in a humidified atmosphere with 5% of CO _2_. Cells were passaged at 70–80% confluency in the following way: after a wash with PBS without calcium and magnesium (homemade), the cells were treated with 0.5 mM EDTA (Sigma-Aldrich, #4055–100mL) in PBS without calcium and magnesium for 2 min at 37 °C. After aspiration of EDTA, cells were dissociated into small clumps in DMEM-F12 (Gibco, #31330038) and seeded with culture media supplemented with 2 µM thiazovivin (Cayman Chemicals, #004CA14245-25). The cell culture medium was replaced with a fresh culture medium without thiazovivin after 16 h. The cells used in the experiments were at passages 50–70 (up to passage 80 for genetic stability experiments). Cells were tested every two weeks for the presence of mycoplasma.

### Growth factor production

Constructs harbouring the growth factors genes (#Qk025, #Qk027, #Qk052, #Qk053: Uniprot number P09038; #Qk001: Uniprot number P08476; #Qk010: Uniprot number P01137, #Qk054; Uniprot number P10600; #Qk045: Uniprot number Q02297) were expressed using a microbial expression system in a β-lactam-free and animal-free environment. All above mentioned growth factors were purified to homogeneity without the use of tags. The bioactivity and purity of the growth factors were assessed and validated to meet industry quality standards.

### Assay for growth factor stability in conditioned media

HEK293 cells (ATCC) were cultured at 37 °C, 5% CO _2_ in Dulbecco’s Modified Eagle Medium (DMEM; Gibco #31966047) and 10 % foetal bovine serum (FBS; Thermo Fisher Scientific #10500064) and plated onto 96-well plates (Corning, #353072) at a density of 1 × 10 ^4^ cells per well. Cells were left to adhere for 24 h before transfection using FuGENE HD (Promega, #E2311) with 50 ng per well DNA total of pGL4.33[luc2P/SRE/Hygro] (Promega, #E1340) for FGF2-G3, or pGL3-CAGA _12_-luc for TGF-β1. Co-transfection with pRL-TK (FGF2-G3) or pRL-SV40 (TGF-β1) (both from Promega) allowed for constitutive expression of *Renilla* luciferase for normalisation. In the case of transfections including the SRE reporter, cells were starved of serum by replacement of media with Opti-MEM I (Gibco, #51985034) for 18 h prior to assay. FGF2-G3 and t-FGF2-G3 (Qkine, #Qk052, #Qk053) and TGF-β1 (Qkine, #Qk010) were reconstituted in water or 10 mM HCl respectively, and then diluted to a concentration of 0.25 mg/mL in HEK293-conditioned media (filtered media from a 3-day culture of HEK293 cells). This was incubated at 37 °C and samples were taken at the indicated time points, and frozen at -80 °C until required. Proteins were serially diluted in OptiMEM (FGF2-G3) or DMEM with 0.5% FBS (TGF-β1) and added to transfected cells in triplicate, before incubation of the plates at 37 °C. After 3 h (FGF2-G3) or 6 h (TGF-β1), the media was removed, and the cells were lysed with Passive Lysis Buffer (Promega). Firefly and Renilla luciferase readings were performed using the FLUOstar® Omega plate reader (BMG Labtech). Firefly/Renilla ratios were calculated for each well and plotted against the protein concentration. Curve fitting to a 4-parameter logistic equation was performed in Prism 10 (GraphPad - https://www.graphpad.com).

### Culture media

Homemade weekend-free media was prepared with DMEM/F12, L-glutamine and HEPES (Gibco, #31330038), and was supplemented with a 100x growth factors supplement prepared in-house. The final concentrations of the supplement components in the media were as follows (also see Extended Data 1 ^6^ for the preparation protocol): for homemade E8 (hE8): 64 µg mL ^-1^ 2-Phospho-L-ascorbic acid trisodium salt (Sigma, #49752-10G), 14 ng mL ^-1^ sodium selenite (Sigma, #S5261-25G), 1743 µg mL ^-1^ sodium bicarbonate (Sigma-Aldrich, #S8875-500G), 10 µg mL ^-1^ transferrin (Optiferrin – Invitria, #777TRF029-1G), 19.4 µg mL ^-1^ insulin (Gibco, #A11382II), 5 ng mL ^-1^ t-FGF2-G3 (145aa, Qkine, #Qk052), 2 ng mL ^-1^ TGF-β1 PLUS (Qkine, #Qk010); for B8+: 200 µg mL ^-1^ 2-Phospho-L-ascorbic acid trisodium salt, 20 ng mL ^-1^ sodium selenite (Sigma, #S5261-25G), 1743 µg mL ^-1^ sodium bicarbonate, 5 µg mL ^-1^ transferrin, 5 µg mL ^-1^ insulin, 5 ng mL ^-1^ t-FGF2-G3, 1 ng mL ^-1^ TGF-β3 (Qkine, #Qk054), 0.1 ng mL ^-1^ NRG-1 (Qkine, #Qk045). Note that since DMEM/F12 already contains sodium bicarbonate at 1200 µg mL ^-1^ concentration, the supplement contains only enough sodium bicarbonate to increase the concentration to the final one described above. In initial optimisation experiments, B8 formulations had alternative sources of FGF2 or TGF-β signalling pathway, such as FGF2, FGF2-G3, t-FGF2, or Activin A1 (Qkine, #Qk027, #Qk053, #Qk025 or #Qk001, respectively). The weekend-free media were compared to commercial E8 – cE8 (Gibco, #A1517001) that was changed daily.

### Adaptation of the cells into the weekend-free media

For each adaptation round, a vial from the same-passage cells expanded in cE8 was thawed and transferred to 2 mL of DMEM/F12, centrifuged at 100 g for 5 min, then resuspended in 2 mL of cE8 with thiazovivin and seeded into 3 wells of a 6-well plate (Corning, #353046), seeding 50, 25, or 12.5% of the suspension, respectively. The cells were then grown in cE8. The next passage was done in cE8, replacing the medium the day after passage with cE8:hE8 or cE8:B8+ at 1:1 proportion. The following day the medium was replaced with the respective weekend free media (hE8 or B8+). After that, these cells were grown and passaged in hE8 or B8+. In parallel, cells from the same vial were grown in cE8 and passaged at the same time as cells in weekend-free media. Cells were grown for at least five passages before performing any assays.

### Flow cytometry analysis of pluripotency markers

Cells that were ready for passage were detached as described before (treated with 0.5 mM EDTA and dissociated in DMEM/F12) and centrifuged at 100 g for 5 min. The cell pellet was resuspended in 600 µL of PBS and transferred on a 96-well plate with a round bottom (Euroclone, #ET3196). The cells were centrifuged at 200 g for 5 min, resuspended in 100 µL of Fixable Viability Dye eFluor 450 (eBioscience, #65-0863-14) diluted 1:1000 in PBS and incubated at room temperature (RT) for 10 min. After three cycles of washes consisting of resuspending cells in 200 µL of FACS buffer (5% FBS – Life Technologies, #10270106 in PBS) and centrifuging at 200g for 5 min, the cells were fixed in 100 µL of 4% PFA (Thermo Scientific, #J61899.AP) at room temperature (RT) for 2 min. Then, the cells were washed three times as above with FACS buffer and permeabilized in 100 µL FACS buffer with 0.75% saponin (Sigma, #8047-15-2) and 0.1% Triton X-100 (Merck, #T8787-100mL) at RT for 40 min. After centrifuging at 200 g for 5 min, the pellet was resuspended with 50 µL of antibodies diluted in permeabilization buffer. For each sample, OCT4/NANOG double staining and respective isotype control staining were performed. In addition, single stained controls were performed on the pooled samples. The following antibodies were used: PE Mouse anti-Oct3/4 (BD Pharmingen, #560186) at 1:10 dilution, AF 647 Mouse anti-Human Nanog (BD Pharmingen, #561300) at 1:50 dilution, AF647 Mouse IgG1, κ Isotype Control (BD Pharmingen, #557714) at 1:500 dilution, PE Mouse IgG1, κ Isotype Control (BD Pharmingen, #556650) at 1:1000 dilution. The stainings were performed at RT for 40 min. After a wash in 150 µL FACS buffer with 0.75% saponin and 0.1% Triton X-100 and a wash in 200 µL of FACS buffer with 0.75% saponin, the cells were resuspended in 400 µL of FACS buffer and analysed on FACSVerse Cell Analyzer flow cytometer (BD Biosciences). Flow cytometry data was analysed on FlowJo 10 (BD Biosciences), using single stained pooled samples to set up a compensation matrix. Doublets were excluded by comparing height to area both in forward and side scatter (FSC and SSC). The gates for live cells were determined by comparing unstained cells with viability dye only cells. The gates for target protein - positive cells were set up based on their respective isotype controls.

### Flow cytometry analysis of cell cycle progression

To label the cells, Click-iT EdU AF 647 Flow Cytometry Assay Kit (ThermoFisher Scientific, #C10424) was used. Cells were fed with respective medium (cE8, hE8 or B8+) containing 10 µM of EdU and incubated in 37 °C for 2 h. Then, cells were washed with PBS and incubated with TryplE™ Express (Thermo Scientific, #12605010): 0.5 mM EDTA in 3:1 proportion at 37 °C for 2 min. Cells were then dissociated in respective cell medium, centrifuged at 100 g for 5 min, resuspended in 200 µL of 1% BSA (Bovogen, #BSAS-NZ) in PBS and transferred to a 96 well plate with a round bottom (Euroclone, #ET3196). Next, cells were centrifuged at 4 °C, 250 g for 5 min, resuspended in 100 µL of eBioscience Fixable Viability Dye eFluor 780 (Invitrogen, #65-0865-14) diluted 1:1000 in 1% BSA/PBS and incubated on ice for 15 min and centrifuged again at 4 °C, 250 g for 5 min. After washing in 200 µL 1% BSA/PBS and centrifugation at 4 °C, 250 g for 5 min, cells were fixed in 40 µL of Click-iT fixative (component D) at RT for 15 min, washed in 200 µL of 1% BSA/PBS and centrifuged at RT, 250 g for 5 min. Then, the pellet was resuspended in 100 µL of 1x saponin-based permeabilization and wash reagent (Component E), incubated at RT for 15 min. 150 µl of Click-iT reaction cocktail was added, mixed well and incubated at RT for 30 min. Then, cells were centrifuged at RT, 250 g for 5 min and washed with 200 µl of component E. The cells were suspended in 200 µL of FxCycle Violet Stain (Molecular Probes, #F10347) diluted 1:1000 in component E and incubated at RT for 30 min in the dark. Without washing, the cells were analysed on FACSVerse Cell Analyzer flow cytometer (BD Biosciences). Flow cytometry data was analysed on FlowJo 10 (BD Biosciences), using single stained pooled samples to set up a compensation matrix. Doublets were excluded by comparing height to area both in forward and side scatter (FSC and SSC). The gates for live cells were determined by comparing unstained cells with viability dye only cells.

### Copy number variation analysis

Cells were cultured as described above for 20 passages after media adaptation. An 80–90% confluent well of a 6 well plate was detached as described above, resuspended in PBS and centrifuged at 100 g for 5 min. The pellet was resuspended in 100 µL of ice-cold PBS. Genomic DNA was extracted using the Monarch Genomic DNA Purification Kit (New England BioLabs, #T3010L), following the protocol for cultured cells (Instruction Manual, Ver 4.1_08/24, page 11 and page 13 - https://www.neb.com/en/-/media/nebus/files/manuals/manualt3010.pdf?rev=624b140ff2834dcb9eecffa430909083&hash=0F214BCD60CC6C285C9A119136B13B12&srsltid=AfmBOop5RrnWXPue0oMYoBW4tHbOrdFLmbw4FtkpTL1ek6e_hiDqMDd5) and eluting the DNA in TE buffer. DNA was then subjected to low resolution karyotyping, using an Illumina microarray containing 700k+ markers. This analysis was performed by Life & Brain company (Bonn, Germany). The SNP array data was analysed in GenomeStudio 2.0.5 with the cnvPartition 3.2.1 plugin installed ( https://emea.support.illumina.com/array/array_software/genomestudio/downloads.html). Using the cnvPartition plugin, the data was analysed for copy number variations with default parameters, except for minimal probe count of 6 and Confidence Threshold of 40. The data was exported as Bookmark Analysis as an .xml file. XML file was parsed and filtered using custom scripts in R (available at https://github.com/alessandro-bertero/homemade_media_code_repository). In short, the CNVs were filtered for confidence threshold (above 85) and the size of the CNV (above 350kbp, or above 100 kbp if in a region known as a CNV hotspot in hPSCs ^7^).

### Bulk RNA sequencing

Cells at 70–80% confluency were washed with PBS without calcium and magnesium, then treated with 0.5 mM EDTA in PBS at 37 °C for 2 min. EDTA was aspirated and cells were detached and suspended in DMEM/F12. The cells were then centrifuged at 100 g for 5 min, the supernatant was then aspirated, and the cell pellet was suspended in 350 µL of Lysis Buffer from quick-RNA Mini Prep kit (Zymo Research, #R1055). Total mRNA was extracted with the same RNA extraction kit, following the kit protocol (Instruction Manual Ver. 4.1.6, page 7). Briefly, the lysates were transferred into Spin-Away Filters, centrifuged at RT, 14000 g for 30 s. The flowthrough was combined with 350 µL of pure ethanol (Sigma-Aldrich, #51976-500ML-F), mixed and transferred to Zymo-Spin IIICG columns and centrifuged at RT, 14000 g for 30 s. The columns were washed in 400 µL of RNA Wash Buffer and centrifuged as above, discarding the flow-through. Then, 80 µL of DNase mix consisting of 5 µL of DNase I and 75 µL of DNA Digestion Buffer were added to each column and incubated at RT for 15 min. After adding 400 µL of RNA Prep Buffer and centrifuging as above, the flow-through was discarded. 700 µL of RNA Wash Buffer was added, centrifuging as above and discarding the flow-through. Then, 400 µL of RNA Wash Buffer was added and the columns were centrifuged at RT, 14000 g for 1 min, discarding the flowthrough. The RNA was then eluted by adding 70 µL of nuclease-free water, incubating at RT for 1 min, then centrifuging at RT, 14000 g, 30 s. The concentration and quality of RNA was evaluated with the Tapestation RNA kit (Agilent, #5067-5576 and #5067-5577). For each sample, 500 ng of the RNA was used to prepare the mRNA library, according to the manufacturer instructions (document nr #1000000124518 v04 - https://support-docs.illumina.com/LP/IlluminaStrandedmRNA/Content/LP/Illumina_RNA/Protocol_SM_ST.htm) of the Stranded mRNA Prep Ligation kit (Illumina, #20040534). Kit-specific Illumina UDI indexes (Illumina, #20040553) were used to allow sample multiplexing on the flow cell. Libraries were quantified with Qubit dsDNA high sensitivity assay kit (Invitrogen #Q332301) prior to pooling. 35M reads were allocated to each library equally, they were sequenced on a Nextseq 1000 (Illumina) and they were loaded on a P2 flow cell of 100 cycles (Illumina, #20100987). They were then sequenced single end by cycle allocation of 10 for both indexes and 118 for read 1.

Demultiplexing was performed with bcl2fastq (Illumina, version 1.8.4 - https://emea.support.illumina.com/downloads/bcl2fastq_conversion_software_184.html) to generate fastq files. Fastq files were QC with FastQC and were trimmed using TrimGalore (version v0.6.10 - https://github.com/FelixKrueger/TrimGalore) in single-end mode. The adapter used is “CTGTCTCTTATACACATCT”, and due to the specificities of the library it was necessary to remove 4 additional bases at the 3’ end and 1 base at the 5’ end. Genome indexing was performed using STAR (version 2.7.11b - https://github.com/alexdobin/STAR) in genomeGenerate mode. The gtf file (Homo_sapiens.GRCh38.111.gtf) and the fasta file (Homo_sapiens.GRCh38.dna_sm.primary_assembly.fa) were downloaded from ensembl. Then data were aligned on the indexed human genome using STAR in GeneCounts mode. Raw count data were processed in R (R version 4.3.3) by using Rstudio server (2023.12.1 Build 402 “Ocean storm” release - https://dailies.rstudio.com/version/2023.12.1+402/) within a docker (Ubuntu 24.04) to ensure reproducibility. Firstly, a unique count matrix was created and filtered using dplyr ( https://dplyr.tidyverse.org) R-package by merging all the nine counts matrices (ReadsPerGenes.out.tab files generated by STAR alignment) and the gene info file (geneInfo.tab file generated by STAR indexing). The final matrix was then filtered by selecting for protein-coding genes and for genes with a minimum of 3 counts in at least one of the nine samples. A metadata table was created with information for each sample on the culture medium and the number of the replicate.

QC analysis was performed on raw data calculating the percentage of mitochondrial counts on the total number of counts in each of the nine samples, then further analyses were performed according to the RNAseqQC pipeline ( https://cran.r-project.org/web/packages/RNAseqQC/vignettes/introduction.html). This pipeline uses the RNAseqQC package and it needs the RNAseq data packed into a Deseq2 object (the DESeqDataSet), which was obtained with the make_dds function by combining the gene counts matrix and the sample information metadata. On the resulting Deseq2 object different analyses were performed: (1) the plot_total_counts function allowed to evaluate the library size; (2) the plot_library_complexity function evaluated the library complexity; (3) the plot_gene_detection function assessed the number of detected genes for each sample; (4) the plot_biotypes function stratified on a graph the total gene counts by their different gene biotypes. According to the pipeline a variance stabilising normalisation was performed using DESeq2 vst function. The obtained output was used for further QC analyses: (1) the plot_chromosome function was used to evaluate differential expression on chromosomal regions for chromosomes 1 to 22, X, Y and mitochondrial chromosome; (2) the plot_sample_MAs to evaluate the variability among the replicates of the same medium. Then data were corrected using the removeBatchEffect function (limma) on the three replicates. Both normalised (vst) counts and normalised (vst) and corrected counts were used for the following analyses: (1) the plot_sample_clustering function generated the Pearson correlation matrix considering the top 1000 most variable genes; (2) the plot_pca_scatters function generated all the possible combinations of PCA plots considering the first nine components; (3) the plot_pca function generated the PCA coordinates and loadings for each component.

Then differential gene expression analysis was performed using limma, glimma and edgeR ^8^. Firstly a design model matrix was created with the model.matrix function (stats) using the information on the culture medium for each sample provided by the meta-data table. The design model was then used to create the contrast matrix with the makeContrasts function specifying the three comparisons taken into consideration, namely B8+ vs hE8, hE8 vs cE8, and cE8 vs B8+. Raw counts were normalised in counts per million (cpm) using the voom function, and then data were corrected with the removeBatchEffect function. Linear model fitting was performed on normalised corrected counts with the lmFit function, the model was then fitted on the contrast matrix with the contrast.fit function, and finally t-statistics was performed with the eBayes function. The *topTreat* function (limma package) was used to extract the results for each comparison, and differentially expressed genes were selected by filtering for an absolute log2 Fold change ≥ 2 and an adjusted p-value < 0.05. Data were normalised starting from the initial raw matrix using TPM normalisation with the NormalizeTPM function (ADImpute). Gene length file was obtained from the gtf file and it is available with the raw data on BioStudies. Log was set to FALSE and scale was set to 1. Normalised data were then corrected with the removeBatchEffect function (limma) on the three replicates (R1, R2, and R3) and used to obtain the gene expression boxplots for the pluripotency marker genes (ggplot2) using the adjusted p-values obtained from the differential gene expression analysis

KEGG pathway enrichment ^9^ was performed using gene lists derived from the differential expression comparisons cE8 vs B8+ (coefficient 1) and hE8 vs cE8 (coefficient 3). For each comparison, only genes with an adjusted p-value < 0.05 were included in the analysis. Firstly, the two lists of genes were converted from ENSEMBL to ENTREZ using the bitr function (clusterProfiler ^10^). Each list was then ranked for decrescent fold change and used as input for the gseKEGG function. The two resulting gseaResult objects were then used into the pathview function. The pathway.id chosen is “hsa04550” (Signalling pathways regulating pluripotency of stem cells). All the R objects created within the analysis can be found in Extended Data 2 ^6^.

### Single-cell RNA sequencing

Cells were detached from 80% confluent wells using TrypLE Express treatment for 4 min at RT. Following detachment, cells were washed in PBS supplemented with 1% BSA (Miltenyi Biotech, #130-091-376) and centrifuged at 100 × g for 5 min. Supernatants were removed, and cells were resuspended for antibody labeling according to the manufacturer’s instructions (10x Genomics, User Guide CG000149 - https://cdn.10xgenomics.com/image/upload/v1660261285/support-documents/CG000149_Demonstrated_Protocol_CellSurface_Protein_Labeling_Rev_D.pdf; protocol 2 for washing steps - page 9). Cells were incubated for 30 min on ice with TotalSeq-B hashing antibodies (BioLegend, TotalSeq -B0251, TotalSeq -B0252, TotalSeq -B0253, TotalSeq -B0254, TotalSeq -B0255, TotalSeq -B0256, cat. nos. #394631, #394633, #394635, #394637, #394639, #394641 respectively) diluted 1:50 in PBS + 1% BSA. Staining was performed in 2 mL low-bind Eppendorf tubes, in a final reaction volume of 100 µL for 1–2 × 10 ^6^ cells. After staining, cells were washed three times with PBS + 1% BSA and centrifuged at 100 g for 5 min between washes. Cells were then counted, and 4 × 10 ^5^ cells per condition were pooled. The pooled suspension was centrifuged at 100 g for 5 min, resuspended in 1 mL PBS + 1% BSA, filtered through a 40 µm cell strainer, and recounted. 2.7 × 10 ^4^ multiplexed cells were loaded into the Chromium Controller (10x Genomics) for an expected recovery of 1.9 × 10 ^4^ final cells. Cells appeared live and round with >90% viability at the time of loading.

Single-cell gene expression and cell surface protein libraries were generated using the Chromium Single Cell 3ʹ v4 workflow (10x Genomics, #PN-1000691, User Guide CG000732 - https://cdn.10xgenomics.com/image/upload/v1710230668/support-documents/CG000732_ChromiumGEM-X_SingleCell3_ReagentKitsv4_CellSurfaceProtein_UserGuide_RevA.pdf). Following reverse transcription, pre-amplification was performed with 11 PCR cycles for both gene expression (GEX) and ADT (antibody-derived tag) targets. cDNA quality and yield were confirmed using the D5000 Agilent TapeStation with the D5000 High Sensitivity kit (#5067-5592) and libraries showed high-quality traces. ADT and GEX libraries were indexed with 11 and 10 PCR cycles, respectively. Final library quality was verified on a D5000 TapeStation. Libraries were pooled with an ADT:GEX ratio of 1:10, then sequenced, aiming at 2 × 10 ^4^ reads per cell on a Nextseq 2000 and an Illumina Xleap P4 100 cycles flow cell (#20100994) and loaded at 650 pM 1% PhiX with a standard 10X protocol run (read 1 = 28; i7 = 10; i5 = 10; read 2 = 90). Demultiplexing and conversion into FASTQ format was performed automatically by the integrated DRAGEN Bio-IT platform on the Nextseq 2000.

Raw data were processed with Cell Ranger 9.0.1 ^11^. Individual GEX libraries were obtained with *cellranger multi*, leveraging on ADT hashtag barcode counts for sample demultiplexing. Alignment to the human genome was performed with the STAR index human GRCh38.p13 (GENCODE v32/Ensembl98). Samples from the same experiment were merged with *cellranger aggr* with *--normalize=none*, adding experimental batch or sample information to the aggregated feature matrix. Cellranger-generated sequencing count matrices (.h5) were imported into Seurat v5 ^12^ using the *CreateSeuratObject* function and cell annotations were added to the Seurat object with *AddMetaData.* Mitochondrial and ribosomal content were quantified using the *PercentageFeatureSet* function to obtain the percentage of mitochondrial and ribosomal reads. Cells were then filtered to retain only those with more than 100 total UMI, more than 350 expressed genes, less than 10% mitochondrial reads, and more than 5% ribosomal reads. Only protein-coding genes with at least 10 total counts in the dataset were kept. Lastly, cell-cycle scores were calculated with *CellCycleScoring* using built-in cc.genes lists for S and G2/M phases. The resulting S.Score, G2M.Score, and discrete Phase. QC-filtered counts were exported to Monocle 3 ^13^ to create a cell_data_set object with the *new_cell_data_set* function with gene metadata from feature names and cell metadata from the Seurat object.

Data were preprocessed and normalized with preprocess_cds with 50 dimensions. Dimensionality reduction was performed using reduce_dimension to obtain UMAP. Qc-filtered counts were exported to Monocle 3 to create a cell_data_set object (new_cell_data_set()) with gene metadata from feature names and cell metadata from the Seurat object. Data were preprocessed with preprocess_cds(num_dim = 50), and dimensionality reduction was performed using *reduce_dimension* to obtain "tSNE" and "UMAP" coordinates. Multiple clustering stringencies were explored with *cluster_cells* and iteration, yielding the most biologically coherent separation across media and replicates (resolution = 5 x 10 ^−5^), which was retained. Cluster identification was obtained by combining the label-transferring information from the single-cell dataset E-MTAB-14065 ^14^, and by differential gene expression comparing the clusters. To this end, the Monocle object was converted to Seurat, then cross-dataset anchors were computed with *FindTransferAnchors,* and cluster labels from the reference were projected into the query with *TransferData.* Cluster-level differential expression was performed in Seurat based on the monocle clusters using the Wilcoxon test within the *FindMarkers* function *.* Comparison of cluster-specific differentially expressed gene sets between iPSC-1 and iPSC2 were filtered based on the fold change and the adjusted pvalue (p_val_adj ≤ 0.01) and log _₂_ fold-change (|log _₂_FC| ≥ 1), then were categorized as upregulated or downregulated according to the log _₂_ fold-change sign. Gene lists were converted from HGNC symbols to Entrez IDs with the *bitr* function of the package ^15^. Overrepresentation analysis was performed using *enrichGO* of the package clusterProfiler ^10^ with ont = "BP" (Biological processes), pAdjustMethod = "BH" (Benjamini-Hochberg p-value correction), and the universe set to all detectable genes.

Human embryo reference transcriptomic profiles were taken from ArrayExpress repositories E-MTAB-3929 ^16^ and E-MTAB-9388 ^17^ for the preimplantation stage (day 3–7) and gastrulation stage (day 16), respectively. For each of the 2 reference datasets, raw counts and metadata were downloaded and combined in a single cell Seurat object with the *CreateSeuratObject* function (SeuratObject package), filtering for a minimum amount of 200 genes expressed per cell. This was then converted into a Monocle 3 object with the *new_cell_data_set* function to further filter for the genes annotated as features in our dataset. Counts were then normalized as the log2 of the number of counts normalized on the library size, increased by 1, and the cell dataset was converted back to a Seurat object with the *as.Seurat* function. The object was processed with the following functions from Seurat : *FindVariableFeatures* (with selection.method set to vst and features set to 8000), *ScaleData* and *RunPCA* (with npcs set to 30). Our single cell dataset was then filtered for the genes annotated in each of the 2 reference datasets, normalized, converted to a Seurat object, and processed in the same way as the references. Anchors were detected with the *FindTransferAnchors* function with k.anchor set to 15, and the timepoint and phenotype labels were respectively transferred from the references with the *TransferData* function using our dataset as query. For the preimplantation embryo dataset the cumulative timepoint distribution was plotted with the stat_ecdf function (ggplot2) after converting the predicted.id labels to integer numbers. For the gastrulation embryo dataset the UMAP was plotted with the ggplot2 package coloring each cell based on the prediction.score.id of the “Nascent mesoderm” identity.

Per-replicate percent cluster composition was calculated with the dplyr package and used to identify the composition of cells in clusters in the different media. Statistics were calculated using an ANOVA with Dunnett-style contrasts (packages emmeans and rstatix), which was employed to test the cE8 cluster composition in comparison to the other media. The differential abundance of cells per cluster in each medium compared to cE8 was calculated with the MiloR package ^18^. The Seurat annotated object was converted to SingleCellExperiment with *as.SingleCellExperiment* function and then wrapped as a MiloR object. For each comparison, the kNN graph was built with the function *buildGraph* with k = 30 and 30 dimensions. This is used by the *makeNhoods* function to generate the overlapping neighborhoods (Nhoods). Pairwise distances between neighborhoods were computed using *calcNhoodDistance* (d = 30). Nhoods-level differential abundance was then tested with *testNhoods*, considering two replicates per sample (media) and cE8 as the reference condition. To then summarize the MiloR analysis to the cluster level, it was calculated (1) the median logFC of Nhoods in each cluster (median_logFC_sig), then (2) the fraction of Nhoods in the cluster that are significant (frac_sig), then (3) whether there is a consistent direction of logFC (all positive vs all negative), and lastly (4) the min_SpatialFDR which corresponds to the minimum value of spatialFDR in the group of Nhoods corresponding to a cluster. Significance was assessed and assigned when min_SpatialFDR<= 0.05 and median_logFC_sig>0.5 or <-0.5 and frac_sig>0.5.

### Transgene integration assay

Cells that were ready for passage were detached with 0.5 mM EDTA as described above and counted using the Bright-Line hemacytometer (Sigma Aldrich, #Z359629-1EA). For each medium, 2 × 10 ^5^ cells per well were seeded on three wells of a 6-well plate (Corning, #353046) that were coated with Geltrex at the matrix density of 15.6 µg cm ^-2^ in their respective medium (cE8, hE8 or B8+) with 2 µM Thiazovivin. The next day, the cells were fed with 1.8 mL per well of their respective medium. This was supplemented with 200 µL of lipofection mix, prepared by combining OptiMEM (Gibco, #31985070) containing 2 µL of Lipofectamine STEM (Invitrogen, #STEM00001), with OptiMEM containing 1 µg of AAV-CAGGS-EGFP (Addgene, #22212, kind gift of Rudolf Jaenisch), 0.5 µg of pZFN-AAVS1_ELD and 0.5 µg of pZFN-AAVS1_KKR (#159297 and #159298, kind gifts of Kosuke Yusa) and incubating the mix at RT for 15 min. Cells were incubated with the lipofection mix for 4 h in the cell incubator, then the medium was replaced with 2 mL of cells’ respective medium. The medium was changed daily. 48 h post lipofection, the cells were fed their respective medium containing 0.5 ng mL ^-1^ Puromycin (Gibco, #A1113803), which was exchanged every day for seven days. On the first two days of puromycin selection, the medium was also supplemented with 2 µM thiazovivin. At the end of selection, all the wells were scanned with Incucyte SX5 Live-Cell Analysis System (Sartorius) with phase contrast and 488 nm channels using the whole well module. For counting colonies, images from 488 nm channels were used. For each condition, an average number of colonies in 3 wells was derived.

### Single cell sorting

Cells were harvested using TryplE:0.5 mM EDTA solution (3:1) and incubated at 37 °C for 3 min. To ensure single cell dissociation, the cell suspension was passed through a 40 µm cell strainer (Pluriselect, #43-10100-40). After centrifugation at 100 g for 5 min, cells were resuspended in sorting buffer: PBS + 1% Penicillin/Streptomycin (Euroclone, #ECB3001D) + 2% FBS + 10 mM HEPES (Fisher Bioreagents, #10756254). Fixable Viability Dye eFluor 780 was added at 1:1000 concentration to the sorting buffer to label dead cells. Live cells were single-sorted on Matrigel-coated (Corning, #356277) 96 well plates (Corning, #353072), coated according to the dilution factor provided by the manufacturer. Plates were prepared with the 3 different media (cE8, hE8 or B8+) supplemented with CEPT cocktail. CEPT cocktail has been prepared with 50 nM Chroman1 (MedChem Express, #HY-15392), 5 µM Emricasan (Selleckchem, #S7775), Polyamine supplement 1:1000 (Sigma-Aldrich, #P8483), 0.7 µM Trans-ISRIB (Tocris, #5284). SH800S Sony cell Sorter with 100 µm Chip and single cells (3 drops) setting have been used for the single cell sorting and cells have been seeded at a maximum speed of 100 events per sec. 72 h after sorting, media were replaced to remove CEPT components and then changed every other day for 1 week. The resulting colonies were stained with Crystal Violet (Sigma, #548-62-9). For the staining, cells were washed with 200 µL of PBS and fixed with 100 µL of PFA 4% at RT 10 min, washed 2 times with 200 µL of PBS, stained with 100 µL of 0.5% Crystal Violet at RT for 10 min and washed 6 times with 200 µL of deionised water to remove the excess dye. Then, the colonies were counted.

### Trilineage differentiation

hiPSC differentiation with the STEMdiff Trilineage Differentiation Kit (StemCell Technologies, #05230) was performed following the manufacturer’s protocol (kit manual, Document #DX20659, Version 1_1_0 - https://cdn.stemcell.com/media/files/pis/DX20659-PIS_1_1_0.pdf). Briefly, cells were seeded as single cells into wells of a 24 well-plate (4 x 10 ^5^ for ectoderm and endoderm differentiation, 1 x 10 ^5^ for mesoderm) with media containing 10 µM ROCK inhibitor (Y-27632 dihydrochloride - Cayman Chemicals, #004CA16644). Every day, the media was changed to the respective ectoderm, mesoderm or endoderm differentiation media provided in the kit. Cells were harvested at day 5 (endoderm and mesoderm) or day 7 (ectoderm) of the differentiation.

### Gene expression analysis

Cells were lysed in QIAzol lysis reagent (Qiagen, #79306) and the lysate was purified using RNA Clean & Concentrator-5 (Zymo Research, #R1016). From each sample, 350 ng of RNA was used to generate cDNA with MultiScribe (Invitrogen, #4311235). The cDNA was then amplified and quantified via qPCR, using PowerUp SYBR Green Master Mix (applied biosystems, #A25743). For each reaction, 2 technical replicates were done, using 10 ng of cDNA and primers at 400 nM. The qPCR was run on QuantStudio 6 Flex (Thermo Fisher Scientific, #4485694). The files were imported to ThermoFisher online analysis tool ( https://apps.thermofisher.com/apps/spa/#/dashboard). After setting the threshold value to 0.4, Cq values were exported for each gene and were compared to the expression of *HPRT1* housekeeping gene. For each gene, the Z-score was calculated separately. Primers used for qPCR are available as Table 1:

**Table 1.** Primers for qPCR used in the study.

Gene	Primer	Sequence (5’-3’)
*HPRT1*	forward	TGACACTGGCAAAACAATGCA
	reverse	GGTCCTTTTCACCAGCAAGCT
*POU5F1*	forward	AGTGAGAGGCAACCTGGAGA
	reverse	ACACTCGGACCACATCCTTC
*NANOG*	forward	CATGAGTGTGGATCCAGCTTG
	reverse	CCTGAATAAGCAGATCCATGG
*FOXA2*	forward	GGAACACCACTACGCCTTCAAC
	reverse	AGTGCATCACCTGTTCGTAGGC
*SOX17*	forward	CGCACGGAATTTGAACAGTA
	reverse	GGATCAGGGACCTGTCACAC
*CDX2*	forward	CTCGGCAGCCAAGTGAAAAC
	reverse	CGGTTCTGAAACCAGATTTTAACC
*TBXT*	forward	TGCTTCCCTGAGACCCAGTT
	reverse	GATCACTTCTTTCCTTTGCATCAAG
*PAX6*	forward	CTTTGCTTGGGAAATCCGAG
	reverse	AGCCAGGTTGCGAAGAACTC
*SOX1*	forward and reverse	Qiagen QuantiTect Primer pair for *SOX* ^18^ *-*#QT00215299

### Directed differentiation in a monolayer towards cardiomyocytes

Cardiomyocytes were differentiated using the previously published protocol ^19^. Briefly, hiPSCs were seeded on the wells of 12-well plate (Corning, #353043), 1.5–3 × 10 ^5^ cells per well in their respective medium (cE8, hE8 or B8+) with 2 µM thiazovivin. The next day, cells were primed for differentiation with 1 mL of their respective medium containing 1 µM CHIR99021 (Cayman Chemicals, #004CA13122). The day after (day 0 of differentiation), cells were induced towards mesoderm with 2 mL of RBA medium, containing RPMI 1640 (Gibco, #11875093), 0.5 mg mL ^-1^ BSA (Sigma-Aldrich, #A8412-100ML) and 213 µg mL ^-1^ L-ascorbic acid 2-phosphate trisodium salt (Fujifilm, #321-44823), supplemented with 5 μM CHIR99021. Two days later, the differentiation towards cardiac mesoderm was continued by adding 2 mL of RBA supplemented with 2 μM WNT-C59 (Cayman Chemicals, #16644). On day 4, medium was replaced with 2 mL RBA. From day 6, cells were fed with 2 mL of RPMI 1640 supplemented with B27 (Thermo Fisher Scientific, #17504044) every other day.

### Flow cytometry analysis of cardiomyocytes

On the 14th day of differentiation, cardiomyocytes were washed with 1 mL of 0.5 mM EDTA in PBS, then treated with 0.5 mL of 0.25% trypsin (Euroclone #ECB3051D) in 0.5 mM EDTA in 37 °C for up to 10 min. The cells were triturated and 0.5 mL of stop solution (RPMI + 10% FBS) was added. After resuspension, cells were centrifuged at 190 g for 5 min, resuspended in 600 µL of PBS and transferred on a 96-well plate with a round bottom (Euroclone, #ET3196). The cells were centrifuged at 200 g for 5 min, resuspended in 100 µL of Fixable Viability Dye eFluor 450 diluted 1:1000 in PBS and incubated at RT) for 10 min. After three cycles of washes consisting of resuspending cells in 200 µL of FACS buffer and centrifuging at 200g for 5 min, the cells were fixed in 100 µL of 4% PFA at RT for 10 min. Then, the cells were washed three times as above with FACS buffer and permeabilized in 100 µL FACS buffer with 0.75% saponin at RT for 40 min. After centrifuging at 200 g for 5 min, the pellet was resuspended with 50 µL of antibodies diluted in permeabilization buffer. For each sample, TNNT2 staining and respective isotype control staining were performed. In addition, single stained controls were performed on the pooled samples. Antibodies used for the stainings: AF 647 Mouse Anti-Cardiac Troponin T (BD Pharmingen, #565744, 1:100 dilution), AF 647 Mouse IgG1, κ Isotype Control (BD Pharmingen, #566011, 1:100 dilution). The stainings were performed at RT for 40 min. After a wash in 150 µL FACS buffer with 0.75% saponin and 0.1% Triton X-100 and a wash in 200 µL of FACS buffer with 0.75% saponin, the cells were resuspended in 400 µL of FACS buffer. Cardiomyocytes were analysed on FACSVerse Cell Analyzer or FACSCelesta Cell Analyzer flow cytometers (both from BD Biosciences). Flow cytometry data was analysed on FlowJo 10 (BD Biosciences), using single stained pooled samples to set up a compensation matrix. Doublets were excluded by comparing height to area both in forward and side scatter (FSC and SSC). The gates for live cells were determined by comparing unstained cells with viability dye only cells. The gates for target protein - positive cells were set up based on their respective isotype controls.

### Cardiac differentiation in left ventricle organoids

Differentiation towards left ventricle cardiac organoids was performed as published ^20^. Briefly, hiPSCs were dissociated as single cells and seeded 5 × 10 ^4^ cells per well on a 24-well plate (Greiner, #GR662160) in a respective medium (cE8, hE8 or B8+) with 5 µM ROCK inhibitor (Y-27632 dihydrochloride - Cayman Chemicals, #004CA16644). 24 h after the seeding, the medium was replaced with Mesoderm induction media. After 36 h, the cells were detached with TryplE Express incubation at 37 °C for 2 min, then resuspended in Cardiac Mesoderm Induction medium with 5 µM ROCK inhibitor. The cells were seeded in a low attachment 96-well plate (Thermo Scientific, #15227905) at 1.5 × 10 ^4^ cells per well and centrifuged at 140 g for 4 min. The next day, the cells are fed with Cardiac Mesoderm Induction medium. For the following two days, the medium was changed to Cardiac Mesoderm Induction medium. For the following two days, the medium was changed to Cardiomyocytes Induction medium.

The media were based on CDM that consisted of 0.4% mg mL ^-1^ BSA (Stem Cell Technology, #100-0177) in 50% IMDM (Gibco, #21980065) plus 50% Ham’s F12 Nutrient Mix with GlutaMAX (Gibco, #31765068), supplemented with 1% concentrated Lipids (Gibco, #11905031), 0.004% monothioglycerol (Sigma, #M6145-100ML) and 15 μg mL ^-1^ of transferrin (Optiferrin – Invitria, #777TRF029-1G). The Mesoderm induction media consisted of CDM supplemented with 6 ng mL ^-1^ FGF2-G3 (Qkine, #Qk035), 5 µM LY294002 (Selleckchem #1105), 5 ng mL ^-1^ Activin A (Qkine, #Qk001), 8 ng mL ^-1^ BMP4 (Qkine, #Qk038), and 5 µM CHIR99021 (Cayman Chemicals, #004CA13122). Cardiac Mesoderm Induction medium consisted of CDM supplemented with 8 ng mL ^-1^ BMP4, 1.6 ng mL ^-1^ FGF2-G3, 10 µg mL ^-1^ Insulin (Thermo Scientific #A11382II), 2 µM Wnt-C59 (Cayman Chemicals, #16644) and 50 nM of retinoic acid (Sigma Aldrich, #R2625). Cardiomyocytes Induction medium consisted of CDM supplemented with 8 ng mL ^-1^ BMP4, 1.6 ng mL ^-1 ^FGF2-G3 and 10 µg mL ^-1^ Insulin.

### Flow cytometry analysis of organoids

After 7.5 days of organoid differentiation, the organoids were pooled together (for each condition, 8 for TNNT2 staining, 8 for isotype staining) and washed two times with 0. 5 mM EDTA in PBS, then incubated with 0.5% trypsin in 0.5 mM EDTA in 37 °C for 15 min, triturated every 5 min. After adding a stop solution, the cells were centrifuged at 4 °C, 200 g for 5 min. The pellet was resuspended in eFluor 450 viability dye, diluted 1:1000 in 1% BSA in PBS and incubated on ice for 18 min. After centrifugation at 4 °C, 240 g for 5 min, the cells were fixed in 4% PFA at RT for 15 min, washed in 1% BSA/PBS and centrifuged at 4 °C, 240 g for 5 min. Then, the cells were suspended in a permeabilization buffer (0.75% saponin in FACS buffer), centrifuged at RT, 240 g for 5 min, resuspended in antibodies diluted in permeabilization buffer and incubated at RT for 40 min. Antibodies used for the stainings: AF 647 Mouse Anti-Cardiac Troponin T (BD Pharmingen, #565744, 1:100 dilution), AF 647 Mouse IgG1, κ Isotype Control (BD Pharmingen, #566011, 1:100 dilution). After a wash with permeabilization buffer, the cells were resuspended in 1% BSA/PBS and analysed on the FACSVerse Cell Analyzer flow cytometer. Flow cytometry data was analysed on FlowJo 10 (BD Biosciences), using single stained pooled samples to set up a compensation matrix. Doublets were excluded by comparing height to area both in forward and side scatter (FSC and SSC). The gates for live cells were determined by comparing unstained cells with viability dye only cells. The gates for target protein - positive cells were set up based on their respective isotype controls.

### Intestinal differentiation into colon organoids

Colon organoid differentiation was performed according to the manufacturer’s protocol (Merck). hiPSCs were dissociated into single cells with Accutase (Merck, #A6964) and seeded at 1 × 10 ^6^ cells per well on a 6-well plate (Falcon, #353046) in cE8, hE8, or B8+ medium supplemented with 5 µM Y-27632. After 24 h, the medium was replaced every day with Definitive Endoderm Induction Medium (Merck, #SCM302) for 3 days, followed by daily changes with Hindgut Induction Medium (Merck, #SCM303) from day 4 to 7, and with Colon Organoid Expansion Medium (Merck, #SCM304) from day 8 to 12.

On day 13, floating organoids and part of the adherent layer were gently detached with EDTA, centrifuged, and embedded in domes of Matrigel (Corning, #734-1101) in 24-well plates (Corning, #353047) at a 1:3 ratio (1:6 for B8+). From this stage, cultures were maintained in custom organoid medium based on Advanced DMEM/F12 (Thermo Fisher Scientific, #12634010) supplemented with N2 supplement (Thermo Fisher Scientific, #17502001), GlutaMAX (Thermo Fisher Scientific, #35050038), 5 mM HEPES (Thermo Fisher Scientific, #15630056), B27 minus vitamin A (Thermo Fisher Scientific, #12587010), 1.25 µM N-acetylcysteine (Sigma-Aldrich, #A9165), 10 mM Nicotinamide (Sigma-Aldrich, N0636-100G), 50 ng µL ^-1^ EGF (PeproTech, #AF-100-15), 10 nM Gastrin (Sigma-Aldrich, #G9145), 500 nM A83-01 (Tocris, #2939), 10 µM SB202190 (Sigma-Aldrich, #S7067-5MG), 3 µM CHIR99021, 50% Wnt3a-conditioned medium, 500 ng µL ^-1^ R-spondin-1 (PeproTech, #120-38), 100 ng µL ^-1^ Noggin (PeproTech, #120-10C), 10 µM PGE2 (Sigma-Aldrich, #P6532-1MG), and 500 nM LY2157299. Organoids were passaged every 7 days at a 1:3 ratio with the addition of 5 µM Y-27632 at each split.

### Whole-mount immunostaining of colon organoids

Organoids were embedded in Matrigel and seeded in 8-chamber slides (Biosigma, #978425), allowed to polymerize at 37°C for 10 min, and overlaid with 300 µL of culture medium. For staining, the medium was removed, and organoids were washed once with PBS, then fixed with 4% PFA at RT for 30 min. After three washes in PBS (5 min each), permeabilization was performed with 0.5% Triton X-100 in PBS at RT for 30 min, followed by three additional PBS washes. Blocking was carried out in 5% normal donkey serum (Merck, #S30-100ML) with 0.25% Triton X-100 in PBS for 30 min at room temperature in a humid chamber. Organoids were then incubated at 4°C overnight with primary antibodies diluted in blocking buffer. The next day, samples were washed three times with PBS (5 min each) and incubated at 4°C overnight with secondary antibodies diluted in blocking buffer. Organoids were then washed 3 times with PBS for 5 min, counterstained with DAPI at RT for 30 min and washed with PBS once for 5 min. Antibodies used for the stainings: rabbit polyclonal anti-human MUC2 (Thermo Fisher Scientific, #PA521329, 1:250 dilution), mouse monoclonal anti-human EpCAM (VU1D9; Cell Signaling Technology, #BK2929S, 1:250 dilution), donkey anti-rabbit Alexa Fluor 488 (Thermo Fisher Scientific, #A21206, 1:500 dilution) and donkey anti-mouse Alexa Fluor 568 (Thermo Fisher Scientific, #A10042, 1:500 dilution). The slides were stored at 4 °C until imaging, which was performed using a Stellaris confocal microscope with a 10x objective (Leica).

### Adaptation of hESCs into the weekend-free media

For the adaptation of hESCs (H1 or H9 - WiCell Research Institute, WA01 or WA09, respectively) into weekend-free media, a vial of cells expanded in cE8 was thawed and transferred to 2 mL of DMEM/F12, centrifuged at 100 g for 5 min, then resuspended in 2 mL of cE8 with 10 µM Y-27632 and seeded into 3 wells of a 6-well plate (Corning, #353046). The cells were then grown in cE8. The next passage was done in cE8, replacing the medium the day after passage with cE8:hE8 or B8+ at 1:1 proportion. The following day the medium was replaced with the respective weekend-free media (hE8 or B8+). After that, these cells were grown and passaged in hE8 or B8+. In parallel, cells from the same vial were grown in cE8 and passaged at the same time as cells in weekend-free media. Cells were grown for at least five passages before performing any assay. H9 cells were maintained in a hypoxia incubator (O _2_/CO _2_ MCO-170M), while H1 cells were maintained in a normoxia incubator during the culture. When passaged, cells were dissociated into clumps of 10–15 cells and seeded in a respective medium (H9 - without thiazovivin, H1 - containing 10 µM Y-27632).

### Extra-embryonic hematopoietic differentiation

Differentiation was initiated after adapting H1 hESCs to the weekend-free media for five passages. The method followed an established protocol ^21^, which involved generation of embryoid bodies (EBs), pre-treatment with WNT signalling, and guided specification towards extra embryonic hematopoiesis. Cells were dissociated using 0.5 mM EDTA and plated onto cell culture dishes coated with 5% (w/v) poly (2-hydroxyethyl methacrylate) (poly-HEMA; Sigma-Aldrich, #P3932) at a density of 5 x 10 ^5^ per well of a 6-well plate in pluripotency medium supplemented with 5 µM Y-27632 and 3 µM CHIR99021. Cells were maintained for 2 hours in a hypoxic incubator under shaking conditions (75 rpm) prior to the initiation of differentiation.

For the first three days of mesoderm specification, cells were maintained in a serum-free differentiation (SFD) medium made of a 75:25 ratio of IMDM to Ham’s F-12 medium (Corning, #10080CVR) supplemented with penicillin (100 IU mL ^-1^), streptomycin (100 μg mL ^-1^), B-27 supplement minus vitamin A, N2 supplement and bovine serum albumin (BSA; Sigma-Aldrich, #126579) at a final concentration of 0.05% w/v. Specific cytokines and small molecules were used to induce extra-embryonic hematopoiesis. On day 0 of differentiation, 10 ng mL ^-1^ of BMP4 (R&D systems, #314-BP/CF) and 10 µM Y-27632 were added to the medium. On day 1, 5 ng mL ^-1^ FGF2 (R&D Systems, 233-FB-500/CF) was added to the culture medium, and Y-27632 was removed. On day 2, 3 µM IWP2 (Cayman Chemical, 004CA13951-25) and 3 ng mL ^-1^ Activin A (Miltenyi Biotec #130-115-010) were added. From day 3 onwards, a Stempro (Thermo Scientific, #10639011) based medium was used. The medium was further supplemented with 1% Glutamine 200 mM (EuroClone, #ECB3000D), ascorbic acid (Sigma-Aldrich, A4403), ITS-X supplement (Thermo Scientific, #51500056), 400 µM monothioglycerol, and 0.1 µL mL ^-1^ ciprofloxacin. Additionally, 10 ng mL ^-1^ of BMP4, 5 ng mL ^-1^ of FGF2 and 15 ng mL ^-1^ of VEGF (R&D systems #293-VE/CF) were added in the medium. On day 6, the basal medium was supplemented with 10 ng mL ^-1^ BMP4, 5 ng mL ^-1^ FGF2, 10 ng mL ^-1^ VEGF, 5 ng mL ^-1^ of IL11 (Miltenyi Biotec, #130-103-439), 10 ng mL ^-1^ of IL6 (Miltenyi Biotec 130-093-934), 2 U mL ^-1^ of EPO (Peprotech, #100-64), 25 ng mL ^-1^ of IGF1 (Miltenyi Biotec, 130-093-887) and SCF (Miltenyi Biotec, #130-096-696). EBs were cultured for a total of 10 days. Throughout the differentiation process, cells were maintained under hypoxic conditions (37°C, 5% CO₂) with constant agitation at 75 rpm.

### Flow cytometry analysis of hematopoietic cells

For flow cytometry analysis, EBs were dissociated into single cells by incubation with 0.25% trypsin-EDTA (Thermo Fisher, #25-200-056) for 8 minutes at 37°C, followed by a 25-minute treatment at 37°C with 2 mg mL ^-1^ collagenase II (Gibco, #17101-015) supplemented with 1mg mL ^-1^ DNase I (Sigma Aldrich, #260913-25MU). The resulting single-cell suspension was subsequently incubated with the antibody mix (diluted in FACS buffer) for 10 minutes at 4°C, washed once in FACS buffer, resuspended in FACS buffer and analysed. Dead cells were excluded based on autofluorescence detected in the PerCP channel. Flow cytometric analyses were performed using a FACS Canto cytometer (BD Biosciences), and data were analyzed using FlowJo software (BD Biosciences). The following antibodies were used for stainings: anti-CD34-PE-Cy7 (BioLegend, #343516, 1:400 dilution), anti-CD43-FITC (BD, #555475, 1:100 dilution), anti-CD45-APC-Cy7 (BioLegend, #368516, 1:100 dilution), anti-CD41a-PE (BD, #555467, 3:100 dilution), anti-CD235a-APC (BD, #551336, 2:100 dilution).

### Neural differentiation into cerebral organoids

Differentiation was initiated after adapting H9 hESCs to weekend-free media for five passages, following an established protocol ^22^, which started with EB generation and proceeded with 3D unguided specification of human cerebral organoids (hCO). Cells were detached with 0.5 mM EDTA and plated at a density of 4 x 10 ^3^ per well of a round bottom ultra-low attachment 96-well plate (Corning #7007). Embryoid bodies were fed every other day for 7-10 days in neural induction medium containing DMEM/F12+ Glutamax (Gibco #31331), 1:100 N2 supplement, minimum essential media-nonessential amino acids (MEM-NEAA) (ThermoFisher #11140050), 0.1 mM 2-mercaptoethanol (ThermoFisher #31350010) and 1 μg/ml heparin (Sigma #H314925KU).

At day 7–10 of neural induction, EBs (400–600 μm in diameter and presented radial organization of pseudostratified neuroepithelium), were embedded into droplets of Matrigel (Corning #356231) located onto organoid embedding sheet (Stemcell Technologies #08579). Matrigel droplets were allowed to gel at 37°C for 1 hour, then transferred to ultra-low attachment 24-well plates (Corning #3473) and fed in neural expansion medium, including a 1:1 proportion of DMEM/F12+Glutamax and Neurobasal (ThermoFisher #21103049), 1:200 N2 supplement, 1:200 MEM-NEAA, 1:100 B-27 supplement without vitamin A, 0.1 mM 2-mercaptoethanol, 1:4000 insulin (Sigma #I9278). The tissues were fed at stationary growth for an additional 4 days and subsequently transferred to ultra-low attachment 6-well plates (Corning #3471) onto an orbital shaker (ThermoFisher #88881101) set at 95 rpm. Cerebral organoids were fed every 3–4 days in the same neural expansion medium, except that B-27 supplement without vitamin A was replaced by B-27 supplement including vitamin A (ThermoFisher #17504044) to further support neuronal differentiation.

### Immunostaining of cerebral organoids

hCOs were fixed in 4% paraformaldehyde for 20 minutes at 4°C and washed three times in PBS for 10 minutes. Fixed organoids were allowed to sink in 30% sucrose solution overnight at 4°C. The next day, the organoids were embedded in Optimal Cutting Temperature compound (OCT; VWR #361603E) and transferred into a plastic mold. Samples were cryosectioned at 12 μm thickness, placed on charged slides (Globe Scientific, #1358W), and stored at -20°C.

For immunostaining, slides were thawed at RT for 15 min, washed in a Conklin jar containing PBS 3 times for 5 min. The leftovers of OCT were wiped with a paper towel. PAP pen (Thermo Fisher Scientific, #R3777) was used to draw a hydrophobic circle around the sections. Samples were permeabilised in 0.5% Triton X-100 in PBS for 5 min, then washed 2 times in PBS. The slides were blocked in 10% FBS in PBS for 1 hour, then incubated with the primary antibody solution (diluted in 1% BSA in PBS) at RT for 1 hour. After 3 washes in PBS, the secondary antibody solution (diluted in 1% BSA in PBS) was added and incubated in the dark at RT for 1 hour. After 5 washes in PBS, the slides were counterstained with Hoechst 33342 (1:2000 dilution in PBS, Invitrogen, #H1399) for 1 min, washed in PBS 2 times, mounted with ProLong Glass Antifade Mounting Medium (Invitrogen, #P36982). Antibodies used in the stainings: goat anti-SOX2 (R&D Systems, #AF2018, 1:100 dilution), mouse anti-beta-tubulin III (Sigma Aldrich, #T8660, 1:400 dilution), rabbit AF 488 anti-mouse IgG (Invitrogen, #A-11059, 1:1000 dilution), donkey AF 657 anti-goat IgG (Invitrogen, #A-21447, 1:1000 dilution).

The slides were imaged on Zeiss Elyra 7, using Plan-Apochromat 20x/0.8 M27 objective (Zeiss, #420650-9901-000) and HiLo imaging settings of Widefield Laser imaging mode. The samples were imaged on the same day, using the same laser and filter settings, covering the entire 12 µm with the Z-stack.

### Statistical analysis

Graphs and statistics were generated using Prism 10 (GraphPad - https://www.graphpad.com - alternatives in the Software availability section) or R. Unless specified otherwise, the bars represent the median value. For Figures 2H, 2I, 5B, H and I, linear mixed-effects model fit by restricted maximum likelihood (REML), implemented via the lmerModLmerTest framework in Rstudio (version 2024.12.0+467). The model included media as a fixed effect and nested random intercepts for adaptation round and differentiation repeat (formula: response ~ media + (1 | adaptation_round/differentiation_repeat)). Post-hoc comparisons were performed using Dunnett’s method, testing pairwise contrasts against cE8. Degrees of freedom were estimated using the Kenward-Roger method, and p-values were adjusted using the Dunnett correction to account for multiple comparisons. For statistical analyses of RNA-seq data ( Figure 3), limma and clusterProfiler were calculating and adjusting the p-values at the level of all analysed genes.

## Results

### Refinement of TGF-β and FGF2 signalling sources and dosage for normoxic hiPSC culture

**Figure 1.**
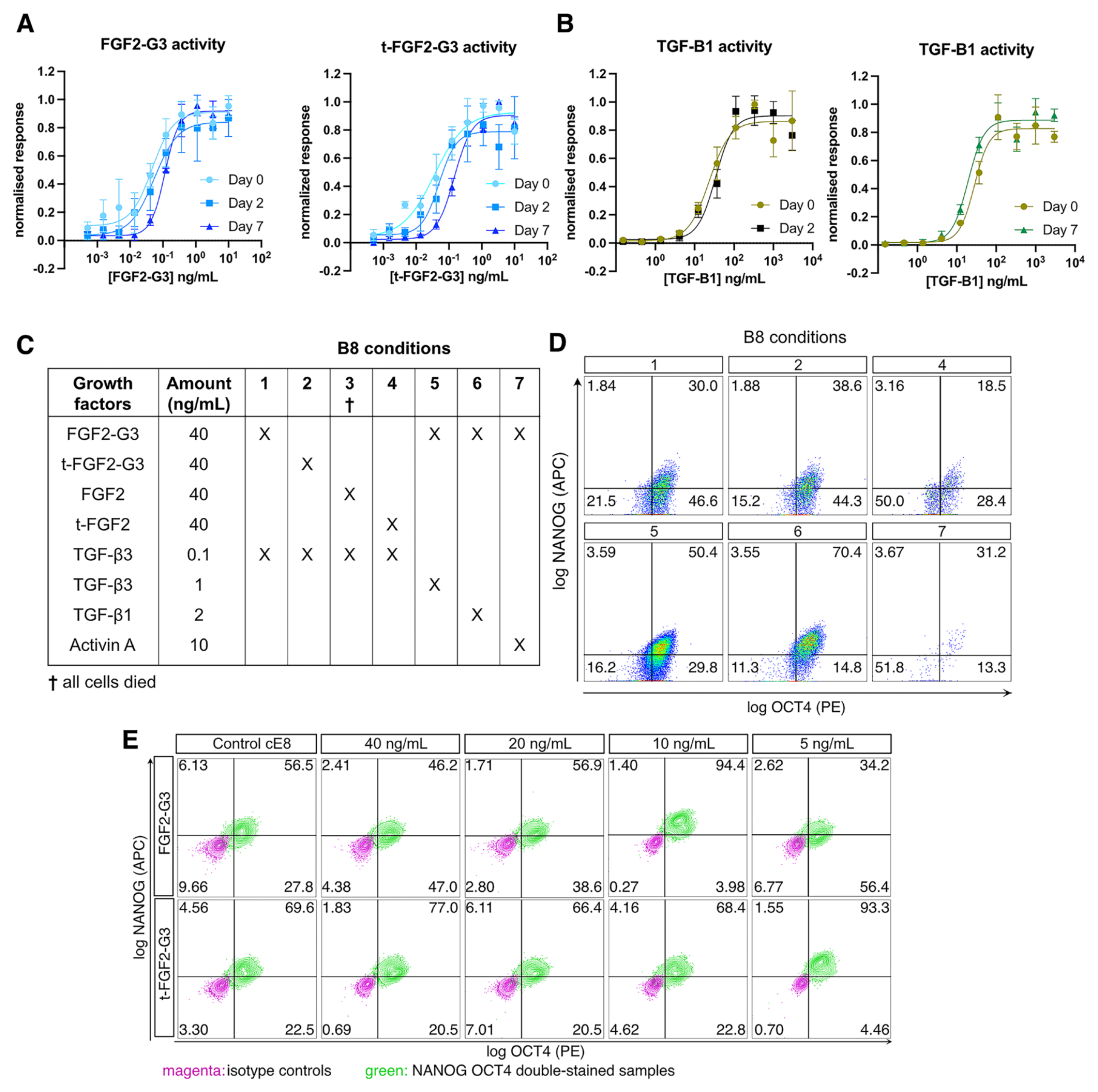
Evaluation of TGF-β and FGF2 sources and concentrations in B8 media. ( **A**– **B**) Variants of FGF2-G3 ( **A**) or TGF-β ( **B**) activity assay using a serum response element luciferase reporter assay in transfected HEK293T cells. Medium pre-incubated in 37 °C for 2 days was compared to the fresh medium. Firefly luciferase activity was normalised to control Renilla luciferase activity. n = 3 experiments. ( **C**) Schematic of B8-based media formulations with different types of FGF2 (standard *versus* thermostable [G3]; full length *versus* truncated [t]) and TGF-β-superfamily growth factor. ( **D**– **E**) Representative flow cytometry data of OCT4 and NANOG expression in hiPSCs grown in media described in panel **C** ( **D**; no data could be acquired for condition 3), or in media with 1 ng/mL TGF-β3 and the indicated concentrations of the truncated or full-length FGF2-G3 ( **E**).

The maintenance of primed pluripotency in hPSCs relies on the combination of signalling through the Activin/Nodal/TGF-β-SMAD2/3 and FGF2-MAPK pathways ^23, 24^. We produced these growth factors using an animal origin-free microbial ( *E.coli*) expression system and without the use of protein tags, as these are not suitable for large-scale manufacture methods, and validated their thermostability ( Figure 1A,B). Besides the full-length FGF2-G3 (154 amino acids, used in the published B8 formulation), we tested a truncated version (t-FGF2, 145 amino acids) that proved marginally more potent at lower doses ( Figure 1A). Both factors retained their activity after 2 days of preincubation in conditioned media with a slight reduction of activity after 7 days of preincubation, suggesting that they are suitable for weekend-free media formulations. Distinctly from unmodified FGF2, TGF-β molecules are not thought to be unstable at 37 °C; we confirmed this to be the case for TGF-β1, as an exemplary molecule from this family ( Figure 1B).

We employed these growth factors to compose various media formulations inspired by B8 ^4^, which we tested by growing a hiPSC line obtained from a healthy male donor, WTC11 ^25^, in normoxic conditions without the daily medium change (weekend-free). As expected, the use of non-thermostable FGF2 or t-FGF2 resulted in pluripotency loss and eventual cell death ( Figure 1C). Surprisingly, however, neither FGF2-G3 nor t-FGF2-G3 supported pluripotency when used in combinations with TGF-β3 at the published concentration of 0.1 ng mL ^-1^ ( Figure 1C,D). As the morphology of hiPSCs cultured in these conditions resembled that of neural rosettes, we speculated that pluripotency loss may result from an excessively low dosage of TGF-β3. Indeed, reduced SMAD2/3 signalling is known to rapidly drive commitment to neuroectoderm through epigenetic and epitranscriptional mechanisms that maintain NANOG expression ^26– 28^. We thus optimised both the source and the concentration of growth factors upstream of SMAD2/3 ( Figure 1C,D). Increasing the concentration of TGFβ-3 by 10-fold (1 ng mL ^-1^) improved the fraction of cells that were double positive for pluripotency markers NANOG and POU5F1 (also known as OCT4). Substituting TGF-β3 with TGF-β1 at a 2-fold higher dose (2 ng mL ^-1^, the concentration reported in the E8 formulation), led to an even greater fraction of NANOG ^+^ OCT4 ^+^ hiPSCs. Activin A, supplemented at 10 ng mL ^-1^ as per the chemically defined media (CDM) formulation ^24^, proved unsuitable in B8-like media.

To improve pluripotency maintenance in B8-like media based on TGF-β3, we then focused on the type and concentration of FGF2-G3. We hypothesised that tag-free FGF2-G3 variants may be too potent when used at the reported concentration of 40 ng mL ^-1^, and tested lower amounts, reducing the concentration down to 5 ng mL ^-1^ for both FGF2-G3 and t-FGF2-G3. ( Figure 1E). Remarkably, 10 ng mL ^-1^ FGF2-G3 and 5 ng mL ^-1^ t-FGF2-G3 proved optimal for pluripotency maintenance, achieving >90% NANOG ^+^ OCT4 ^+^ ( Figure 1E). We elected to use t-FGF2-G3, which reduces the cost of FGF2 sourcing by 2-fold, and named the resulting formulation B8+, to distinguish it from the original B8 (See Figure 1).

### B8+ supports hiPSC proliferation and self-renewal

**Figure 2.**
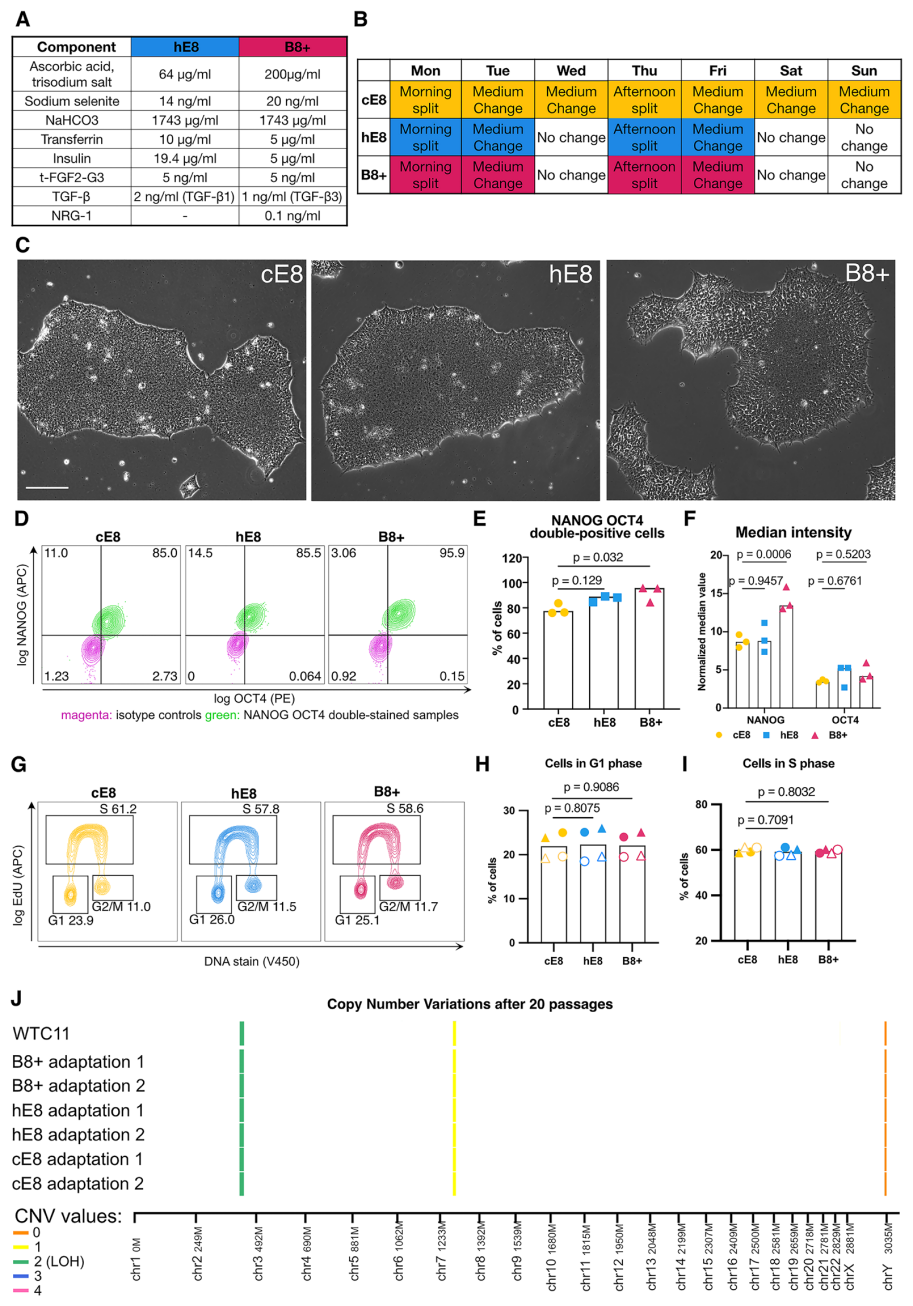
Characterization of hiPSCs adapted to weekend-free media. ( **A**) Final concentrations of components in the supplements for two weekend-free media compositions (both based on DMEM/F12). ( **B**) Weekly schedule of hiPSC culture in three different media. Days highlighted in colour involve media changes. ( **C**) Phase-contrast images of representative hiPSC colonies 72 h after passage. Scale bar: 200 µm. ( **D**) Representative flow cytometry analyses of OCT4 and NANOG expression in hiPSCs grown in different media. ( **E**– **F**) Flow cytometry quantifications of the percentage of NANOG ^+^ OCT4 ^+^ cells ( **E**) and the normalised median fluorescence intensity value of NANOG and OCT4 ( **F**). Median values were normalised through division by the median intensity value of the respective isotype control. n = 3 independent adaptations of the cells into the media. Statistical analyses by one-way ANOVA followed by Dunnet’s multiple comparisons test. ( **G**) Representative flow cytometry cell cycle analyses in EdU-treated cells stained for DNA content. ( **H**– **I**) Flow cytometry quantifications of the percentage of cells in G1 ( **H**) or S ( **I**) phase. Circle and triangle represent 2 independent media adaptations, while the filled/empty symbols represent 2 repetitions of the experiment. Statistical analyses by mixed effects model followed by Dunnet’s multiple comparisons test. ( **J**) Copy number variation (CNV) analysis of cells after 20 passages in the media. Colored bands represent the number of copies of detected CNVs (or loss of heterogeneity - LOH). n = 2 independent media adaptations.

Having developed a promising media formulation, we set out to comparatively assess it in various applications routinely performed by our group. Besides B8+, we designed a homemade E8-based formulation (hE8) by substituting FGF2 at 100 ng mL ^-1^ with t-FGF2-G3 at 5 ng mL ^-1^ (to enable weekend-free culture and reduce costs) and by equalising the NaHCO _3_ concentration in hE8 and B8+ (to allow a more rigorous comparison; Figure 2A). We adapted WTC-11 hiPSCs to B8+ and hE8 culture according to a weekend-free schedule for at least five passages and compared them to passage-matched hiPSCs cultured in commercial E8 (cE8) grown according to the manufacturer's recommended protocol with daily media changes ( Figure 2B).

First of all we examined hiPSC morphology, which proved different. While cells grown in cE8 formed compact colonies 3 days after seeding in small clumps, cells grown in both weekend-free media occupied more space (Extended Data 3 ^6^). This was more evident in B8+, where the colonies were the least compact ( Figure 2C). This morphology is similar to the one described for the original B8 formulation ^4^.

To verify whether this shift in morphology reflects a change in pluripotency, we analysed the levels of NANOG and OCT4 expression by flow cytometry ( Figure 2D). We observed a high fraction of NANOG ^+^ OCT4 ^+^ double positive cells in all the media, with B8+ supporting the highest proportion of pluripotent cells ( Figure 2E). In addition, cells grown in B8+ exhibited increased levels of NANOG expression ( Figure 2F).

To assess whether cell proliferation is affected, we investigated how many cells are in G1 phase, which is very short in iPSCs ^29^. Similar proportions of cells were in G1 phase for all the media ( Figure 2G,H), as well as in S phase ( Figure 2G,I), suggesting a similar division rate in all the media. In all, we concluded that both weekend-free media formulations effectively support hPSC pluripotency (See Figure 2).

To verify the genomic stability of the cells in weekend-free media, we cultured them for 20 passages (equivalent to 10 weeks in culture). In that time, the cells also underwent two cycles of freezing and thawing, an additional potentially stressful procedure. We analysed their genomic DNA for copy number variation (CNV). In all of the instances, cells did not accumulate new mutations as compared to the parental cell line (WTC-11, Figure 2J), demonstrating that the weekend-free media do not grossly impact the genomic stability and are suitable for long-term culture of cells.

### Weekend free media differentially affect the transcriptional state of hiPSCs

**Figure 3.**
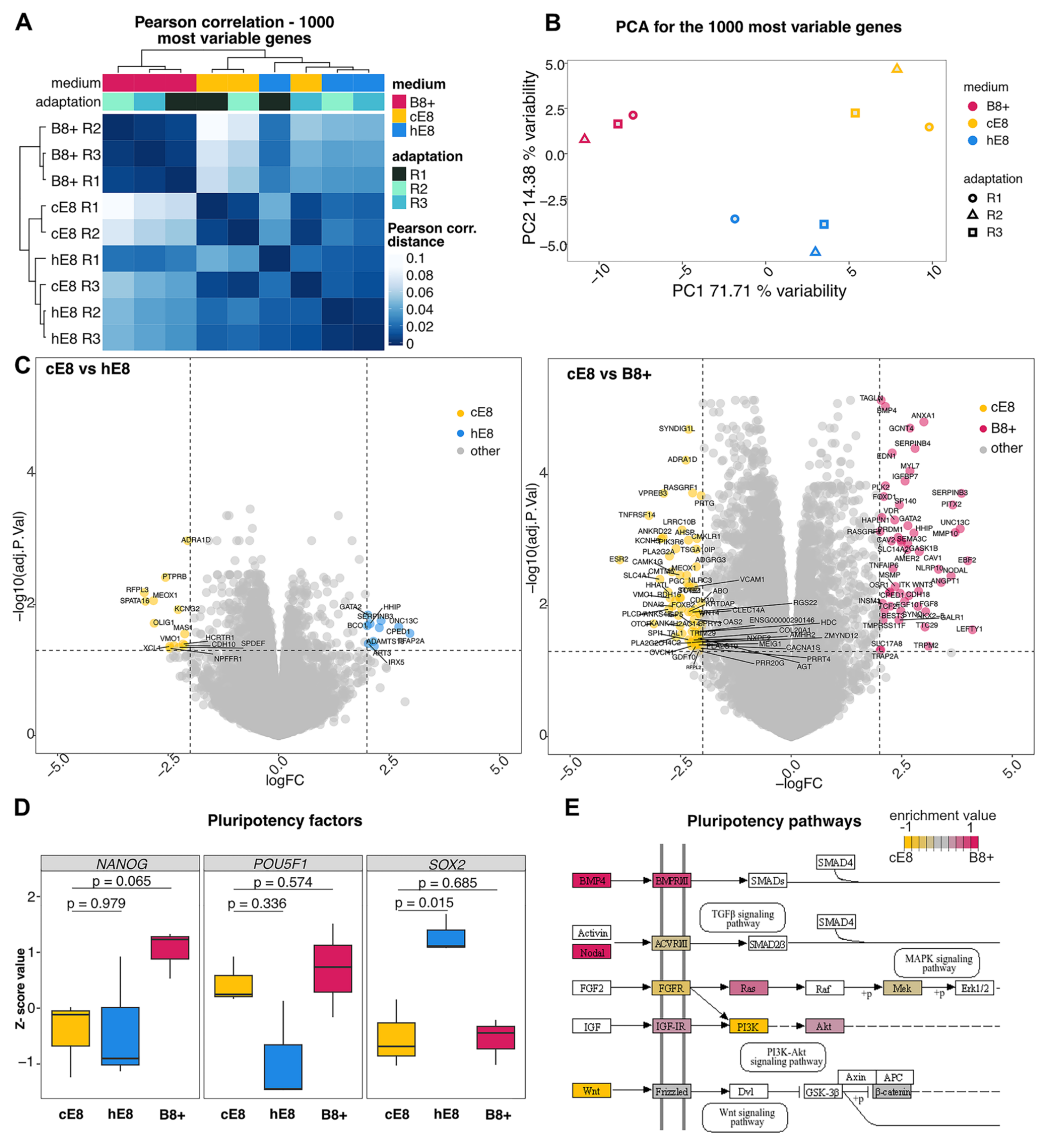
Effects of weekend-free media on gene expression. ( **A**– **B**) Pearson correlation ( **A**) and Principal Component Analysis (PCA; **B**) based on the expression of the 1000 most variable genes in bulk RNA-seq measurements of hiPSCs adapted to the indicated media. n =3 independent adaptations of the cells into the media. ( **C**) Volcano plots showing differentially expressed genes in cE8, hE8 or B8+ in comparison to cE8. Significantly different genes are highlighted in color. ( **D**) Changes in gene expression of key pluripotency genes, measured as a Z-scores of TPM normalised counts. P-values are calculated by linear model fitting and are adjusted for Benjamini-Hochberg false discovery rate. ( **E**) Schematic of genes involved in primed pluripotency signalling (according to KEGG pathways); those significantly upregulated in cE8 or B8+ are coloured in shades of yellow or red, respectively.

To verify the transcriptional state of the cells growing in different media, we first performed bulk RNA-sequencing (See Figure 3). Pearson correlations and principal component analysis (PCA) indicated that independent adaptations to B8+ and hE8 led to reproducible changes in gene expression compared to cE8 ( Figure 3A,B). As anticipated, cells grown in hE8 are more similar to those in cE8, while the gene expression profile of cells cultured in B8+ is more different from the other two conditions.

Specifically, differential gene expression analyses revealed 117 genes differentially expressed in B8+ *versus* cE8 (53 up- and 64 down-regulated), while only 24 genes were differentially expressed in hE8 versus cE8 (10 up- and 14 down-regulated, all filtered for adj. p-value < 0.05 and an absolute log _2_ fold change of 2; Figure 3C). In line with flow cytometry analyses, the expression of *NANOG* was highest in B8+ ( Figure 3D). Both weekend-free media showed similar levels of *POU5F1/OCT4*, and hE8 expressed a higher level of *SOX2* compared to cE8 ( Figure 3D). Analysis of pluripotency KEGG pathways revealed that autocrine Nodal and BMP4 were upregulated in B8+ compared to cE8, while WNT ligands were downregulated ( Figure 3E).

### Weekend-free media differentially alters hiPSC heterogeneity

**Figure 4.**
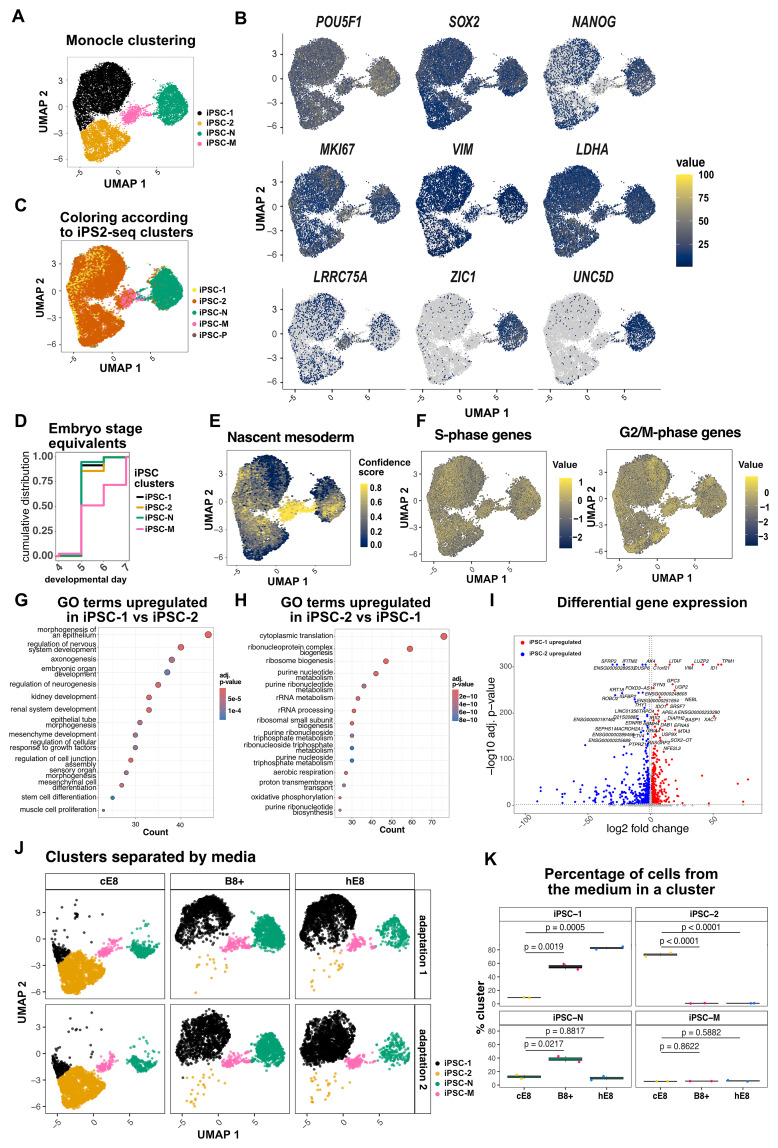
Heterogeneity in hiPSCs adapted to weekend-free media. ( **A**) Aggregated Monocle clustering of single cell RNA-seq data from two individual media adaptations of hiPSCs to cE8, hE8 and B8+. ( **B**) Expression levels of representative lineage markers. ( **C**) Cell labeling according to previously established subpopulations of hiPSCs ^14^. ( **D**) Cumulative frequency distribution of cells from each cluster based on the human embryo developmental day they correspond most closely to ^16^. ( **E**) Expression of transcriptional signature of nascent mesoderm across the clusters. ( **F**) Scores based on the expression of genes characteristic for S or G2/M phase of the cell cycle. ( **G**- **H**) Gene Ontology terms enriched in genes overexpressed in iPSC-1 ( **G**) versus iPSC-2 ( **H**) clusters. ( **I**) Volcano plot showing differentially expressed genes in iPSC-1 versus iPSC-2 clusters Significantly different genes are highlighted in color. ( **J**) Cluster assignment of hiPSCs adapted to weekend-free media. ( **K**) Proportion of cells from the particular media assigned to the cluster. Statistical analysis by one-way ANOVA followed by a Dunnet’s multiple comparison’s test.

Intrigued by the coarse differences observed in bulk RNA-seq, we next sought finer resolution by performing single-cell transcriptomic analyses to dissect population heterogeneity across media conditions (See Figure 4) Using 10x Genomics microfluidics, we profiled >2,000 cells per condition in two biological replicates. Aggregated analysis revealed an unexpected degree of diversity, with at least four clearly distinct subpopulations ( Figure 4A).

All subpopulations expressed similar levels of the core pluripotency factors *POU5F1/OCT4* and *SOX2*, but differed in the levels of *NANOG*, which was highest in a population that paradoxically co-expressed the neuroectoderm competence factor *ZIC1* ( Figure 4B). We recently showed that *ZIC1*+ hiPSC clones are biased towards neuroectodermal lineages, leading to their depletion during cardiac differentiation and altered specification in forebrain derivatives ^14^. While those observations were made in mTeSR Plus, an unrelated medium formulation, integrative analysis of the two datasets confirmed a strong similarity between the *ZIC1*+ populations ( Figure 4C). Accordingly, the top surface marker we had previously identified, *UNC5D*, was exclusively expressed in this population, which we tentatively labelled iPSC-neuro.

A second, smaller subpopulation corresponded to cells we had previously observed only in hiPSCs transiently primed for mesodermal fate by WNT signalling activation ( Figure 4C). Consistently, this cluster specifically expressed *LRRC75A* ( Figure 4B), one of the few markers we could assign to this transient state. To further examine this state, which had not been extensively characterized in our earlier work, we compared its transcriptional profile with that of the developing human gastrula ^16, 17^. Cells from this cluster appeared more developmentally advanced ( Figure 4D), and displayed a strong transcriptional signature characteristic of nascent mesoderm ( Figure 4E). Based on these features, we identified this cluster as iPSC-meso.

The two remaining major subpopulations, designated iPSC-1 and iPSC-2, had not been characterized in our previous studies. To exclude the possibility that these reflected different cell cycle states, we examined proliferation signatures: both clusters contained cells with high S and G2/M indices ( Figure 4F), and expression of proliferation markers such as *MKI67* was distributed across all four subpopulations ( Figure 4B), ruling out a simple cell cycle-based artifact. Gene ontology analyses of differentially expressed genes between iPSC-1 and iPSC-2 revealed a mild enrichment of morphogenesis-associated terms in iPSC-1 ( Figure 4G), while iPSC-2 was strongly enriched for biosynthetic processes ( Figure 4H). For example, iPSC-1 showed elevated expression of the BMP target *ID1* and the early mesenchymal marker *VIM*, whereas iPSC-2 expressed higher levels of the glycolytic enzyme *LDHA* ( Figure 4B,I).

Having established a tentative interpretation of subpopulation diversity, we next examined their relative abundance across the three media conditions ( Figure 4J,K). iPSC-1 represented the majority of cells in both hE8 and B8+, in contrast to cE8, where iPSC-2 was strongly predominant. These results indicate that weekend-free culture induces a subtle but consistent shift in hiPSC transcriptomes, likely reflecting changes in metabolic state and/or autocrine signalling associated with intermittent media starvation.

Despite these similarities between the two weekend-free media, we observed a striking difference with regard to the iPSC-neuro population. While hE8 slightly reduced its representation compared to cE8 to less than 10%, B8+ increased it to ~40% of the population ( Figure 4K). An orthogonal analysis with Milo ^38^ of differential abundance at the neighbourhood level confirmed these shifts across replicates (Extended Data 4 ^6^). Thus, while both weekend-free formulations affect the metabolic state of hiPSCs, B8+ specifically results in a neuroectoderm lineage bias.

### Weekend-free media support hiPSC genome editing, single cell cloning, and differentiation

**Figure 5.**
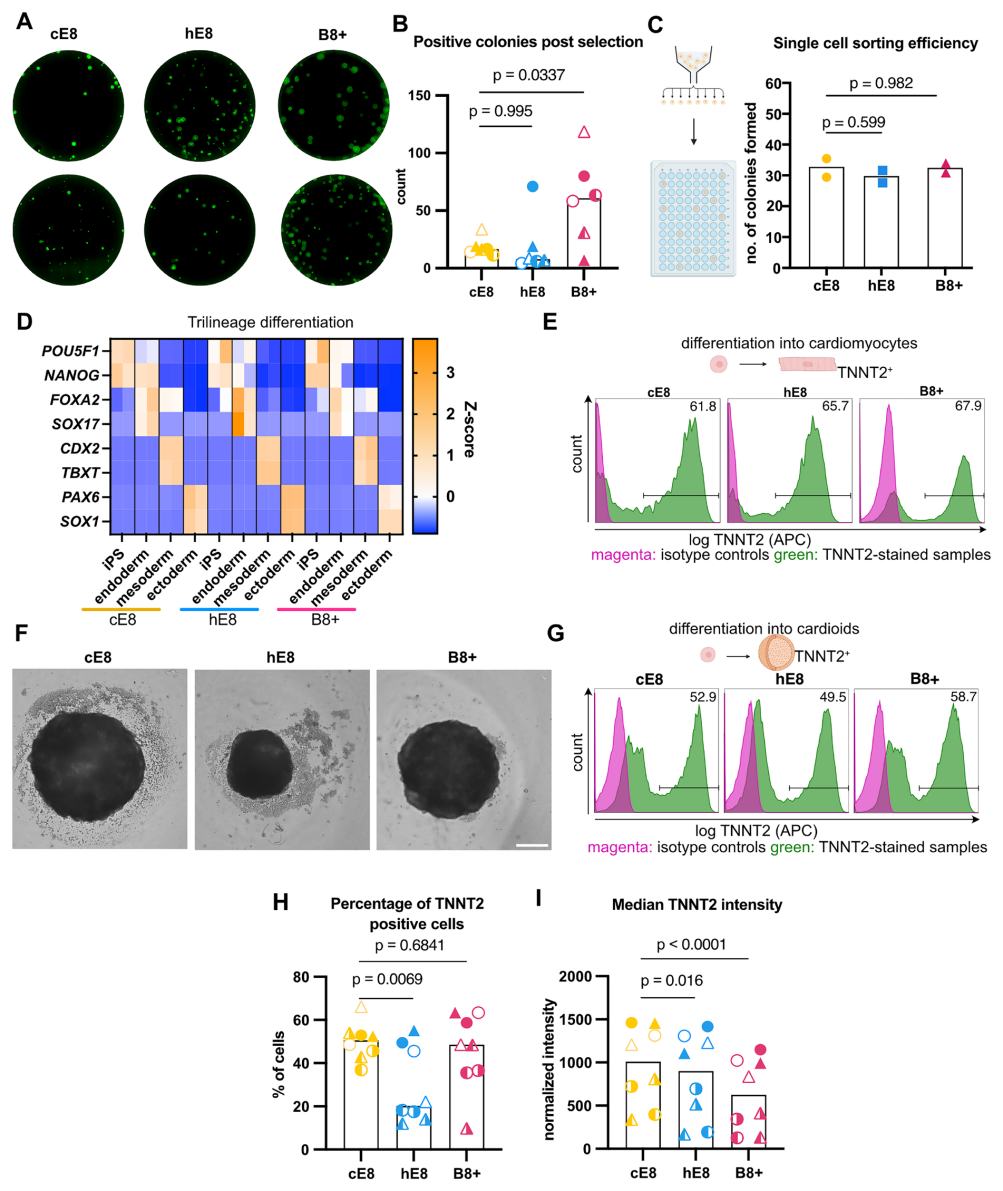
Applications of hiPSCs adapted to weekend-free media. ( **A**) Representative images of GFP-positive iPSC colonies after puromycin selection of *AAVS1* genome-edited cells. ( **B**) Quantification of the positive colonies. Circles and triangles represent 2 independent media adaptations, while the different types of symbol filling represents 3 repetitions of the experiment. Statistical analyses by mixed effects model followed by Dunnet’s multiple comparisons test. ( **C**) Quantification of hiPSC clonality after single cell sorting into 96-well plate. n = 2 media adaptations. ( **D**) Z-scores of gene expression levels in hiPSCs adapted to weekend-free media and the same cells differentiated into three germ layers, as measured by RT-qPCR. n = 2 media adaptations. ( **E**) Exemplary flow cytometry analyses of TNNT2 expression in hiPSCs successfully differentiated towards cardiomyocytes in monolayer ( **F**) Exemplary brightfield images of organoids derived from hiPSCs. Scale bar: 500 µm. ( **G**) Exemplary flow cytometry analyses of TNNT2 expression in hiPSCs successfully differentiated towards cardiomyocytes in organoids. ( **H**– **I**) Flow cytometry quantifications of the percentage of TNNT2-positive cells ( **H**) and the normalised median TNNT2 value ( **I**) in cardiac organoid differentiations. Median values were normalised through division by the median intensity value of the respective isotype control. Circles and triangles represent 2 independent media adaptations, while the different types of symbol filling represents 4 repetitions of the experiment. Statistical analyses by mixed effects model followed by Dunnet’s multiple comparisons test.

In light of these transcriptional differences, it was important to assess whether hiPSCs maintained in weekend-free media could not only be sustained long term, but also remain suitable for typical applications. First, we examined their ability to undergo genome editing. For this, we co-transfected hiPSCs with plasmids encoding zinc finger nuclease (ZFN) against the *AAVS1* genomic safe harbour and a targeting vector with homology regions to such locus and carrying both a constitutive EGFP and a gene trap-based puromycin resistance gene ^30^. We then selected with puromycin hiPSCs which had undergone site-specific integration via homology-directed repair and validated the result through fluorescence microscopy for EGFP. These experiments indicated that hiPSCs gave rise to a similar number of genome-edited colonies in hE8 and cE8, while an even larger number was obtained in B8+ ( Figure 5A,B).

Encouraged by these results, we also tested the survival and growth of individual hiPSCs following fluorescence activated single-cell sorting), a procedure commonly used to generate single cell clones. To this end, we single-sorted cells into 96-well plates and counted the number of colonies formed after a week of culture. All conditions resulted in ~30% clonality, ( Figure 5C), indicating that hiPSCs tolerate this stressful procedure equally well in weekend-free media compared to cE8.

Lastly, we tested whether weekend-free culture affected the differentiation capacity of hiPSCs. We first tested trilineage differentiation, and reverse transcription quantitative PCR (RT-qPCR) indicated a comparable induction of markers representative of all three germ layers ( Figure 5D), confirming preserved pluripotency. To further assess directed differentiation, we next induced cardiomyocyte specification. While the initial seeding density had to be adjusted for each medium, we could ultimately achieve a high differentiation rate for cells cultured in all conditions ( Figure 5E), indicating that all media can support the ability of hiPSCs to differentiate into mesodermal derivatives. We next investigated the potential of hiPSCs to differentiate into more complex, three-dimensional organoids, using cardiac organoids as an example. hiPSCs cultured in all media were able to form the organoids, although these were smaller in B8+ and, particularly, in hE8 ( Figure 5F). In an initial set of two biological replicates from independently adapted cultures, differentiation into TNNT2+ cardiomyocytes was comparable across the three conditions. However, a subsequent set of two replicates was less efficient in hE8 ( Figure 5G,H). Moreover, we consistently observed lower levels of expression of cardiac troponin in cells grown in B8+ and marginally lower for hE8 ( Figure 5I). Overall, these experiments indicate that hiPSCs can be grown in homemade weekend-free media and be used in a variety of applications, although some differentiation protocols may require re-optimization to ensure reproducible results.

### Weekend-free media performs well across laboratories, cell lines, and differentiation assays

**Figure 6.**
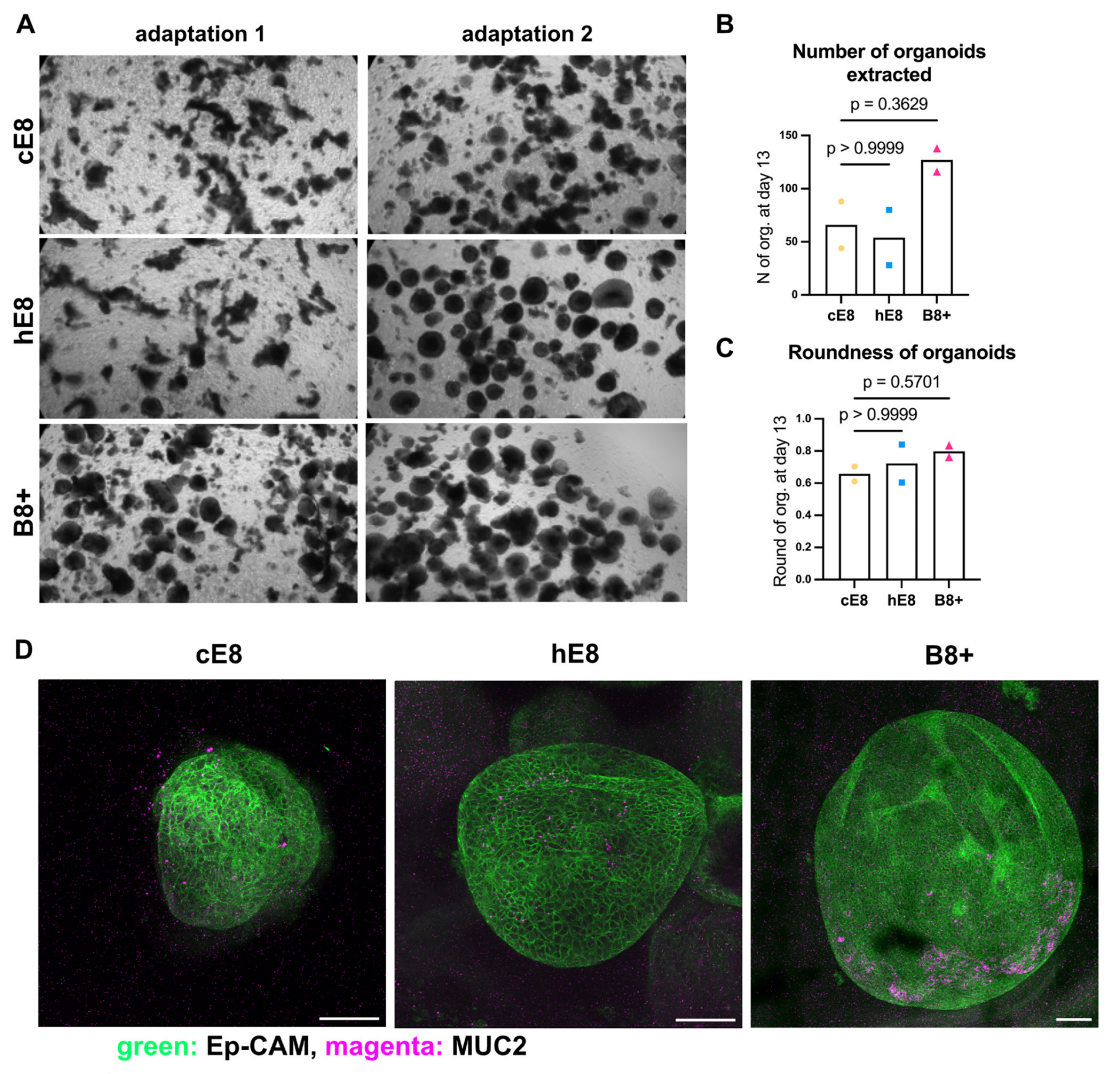
Differentiation of hiPSCs into colon organoids. ( **A**) Brightfield images of budding endodermal layers derived from hiPSCs adapted to different media. ( **B**- **C**) Quantifications of number ( **B**) and the roundness ( **C**) of organoids extracted from the monolayers at day 13 of differentiation. ( **D**) Images of whole-mount immunostainings of mature colon organoids. Scale bar: 100 µm.

To test whether the performance of weekend-free media extends beyond our own laboratory, we transferred WTC-11 hiPSCs adapted to these conditions to another group within our institute, where they were successfully maintained and then differentiated into colon organoids (See Figure 6). In all conditions cells initially expanded as monolayers formed budding organoids ( Figure 6 A–C) that, when cultured long-term, acquired characteristics of colon tissues such as MUC2 expression ( Figure 6D). Of note, cultures in B8+ generated a higher number of early organoids, which appeared rounder and expanded more extensively over time.

**Figure 7.**
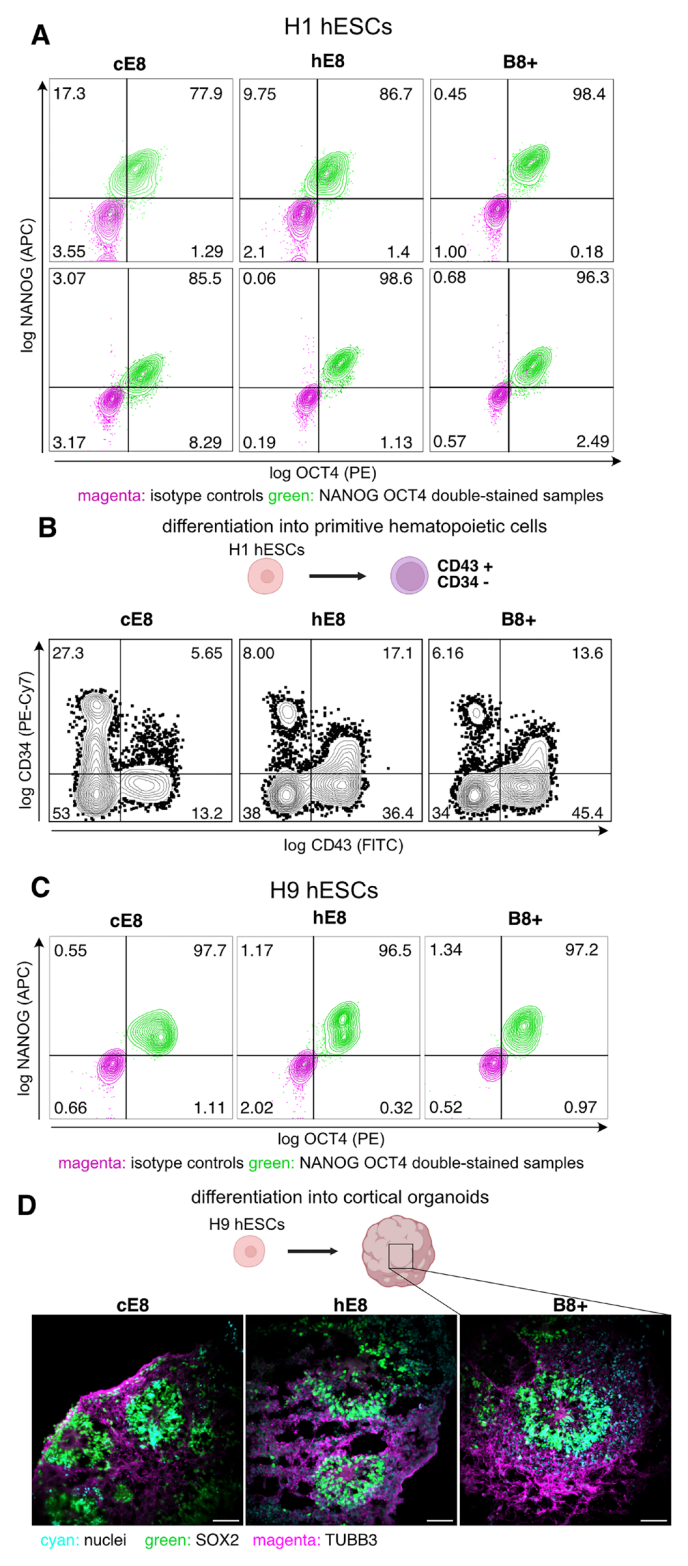
Adaptation of hESC lines to weekend-free media. ( **A**) Flow cytometry analyses of OCT4 and NANOG expression in H1 hESCs grown in different media. The results are shown for two independent media adaptations. ( **B**) Flow cytometry analysis of CD43 and CD34 expression of H1 hESCs differentiated towards primitive hematopoietic stem cells. ( **C**) Flow cytometry analysis of OCT4 and NANOG expression in H9 hESCs grown in different media. ( **D**) Immunostainings of sectioned human cortical organoids derived from H9 hESCs. Scale bar: 50 µm.

Because individual hPSC lines may vary in their requirements for growth factor concentrations, we next recruited two other collaborating groups to assess whether our weekend-free formulations could support additional commonly used cell lines (See Figure 7). We selected one male (H1) and one female (H9) hESC line to increase diversity ^31^. Adaptation of both lines to weekend-free did not impair pluripotency, as shown by the fraction of the NANOG and OCT4 double positive cells ( Figure 7A, C). Notably, H9 were maintained in hypoxic conditions and H1 in normoxia, due to different standard operating procedures, demonstrating compatibility of the weekend-free media with different oxygenation settings. As additional proof-of-principle, directed differentiation into derivatives of different germ layers was successful: H1 cells generated an even larger number of CD34 and CD43 double positive hematopoietic progenitor cells in both hE8 and B8+ compared to cE8 ( Figure 7B); H9 cells generated large cortical organoids with TUBB3 and SOX2 positive ventricles in all conditions ( Figure 7D).

Together, these results indicate that homemade weekend-free media are sufficiently robust to support different hPSC lines, diverse culture conditions, and complex differentiation assays across laboratories.

## Discussion

To unlock the full potential of hPSC-based research, their culture, genome editing, and differentiation need to be made more accessible by reducing the workload and financial burden on laboratories. Weekend-free media, such as B8, offer such possibility without compromising cell growth. Production of functional and endotoxin-free FGF2-G3 is complex for individual academic laboratories and requires a licence for the patent. Moreover, in our hands applying the direct formulation of B8 using commercially-available reagents resulted in loss of pluripotency. To overcome this issue, we developed the B8+ formulation, which relies on a 10-fold higher concentration of TGF-β3 but allows an 8-fold lower concentration of FGF2-G3, largely offsetting the increased cost.

TGF-β1 and TGF-β3 are generally considered equivalent in supporting hPSC growth, although TGF-β3 can be more potent in certain contexts such as cell migration ^4, 32^. In our B8+ formulation, pluripotency was maintained with TGF-β3 at 1 ng/mL. While this is higher than the 0.1 ng/mL reported in the original B8 recipe, it remains half the concentration of TGF-β1 used in cE8 (2 ng/mL), which has been reported as necessary for maintaining pluripotency ^4^. We therefore retained TGF-β3 in B8+, as it supports pluripotency at a lower concentration than TGF-β1, even if the cost advantage compared to cE8 is more modest than originally anticipated. Moreover, the original B8 formulation employed TGF-β3, reportedly easier to express in bacteria than TGF-β1 ^4^, although later work from the same group recommended sourcing TGF-β3 commercially because its low yield made in-house production not cost-effective ^5^. Notably, all growth factors used in this study were produced in E. coli, indicating that expert biomanufacturing can mitigate this limitation.

Our suboptimal results with B8 may be caused by differences in cell culture conditions. Most importantly, B8 was validated using hPSCs cultured in hypoxic conditions (5% O _2_, 5% CO _2_), while our experiments were performed in standard normoxia incubators (5% CO _2_). We speculate that lower doses of TGF-β3 are tolerated in hypoxia, which is more optimal for maintaining pluripotency ^33^. A convergence of hypoxia and TGF-β signaling in regulating the cell cycle was described for hematopoietic stem cells ^34^. A similar convergence could happen in hPSCs e.g. via NANOG expression, a known TGF-β signalling target which is also influenced by hypoxia ^35^. This phenomenon could allow lowering the concentration of TGF-β below the levels tolerated in normoxic conditions. However, most laboratories do not have access to an hypoxic incubator, and thus normoxic culture of iPSCs is widely used. While B8 is more efficient in cutting media costs, B8+ and its derivative hE8 may prove to be more universal formulas, as they maintained pluripotency in one hiPSC and one hESC line cultured in normoxia, as well as in one hESC line grown in hypoxia. In the future it would be interesting to rigorously compare these two oxygenation conditions in B8, B8+, and hE8, and investigate the mechanisms affected by these variables. Of note, we cannot rule out that WTC-11 hiPSCs are particularly sensitive to TGF-β levels, but we deem it unlikely since they are widely used and have been cultured in a variety of commercial media without any issue. We eagerly anticipate tests of B8 versus B8+ and hE8 in other hiPSC lines and by other laboratories in order to gauge the reproducibility of our findings.

The reduced requirements for FGF2-G3 in our weekend-free formulation could be partially due to the absence of the 6xHis tag, though a previous report indicated that this would not interfere with FGF2 activity ^36^. Alternatively, employing a rigorous manufacturing process along with stringent quality control procedures ensures high purity and homogeneity in the produced growth factor, resulting in a higher fraction of bioactive FGF2-G3. Lastly, we could reduce the concentration of FGF2-G3 by an additional 2-fold by employing a truncated version comprising the core structured region (145 aa) sufficient for full biological activity.

Preparing weekend-free media from commercially-sourced growth factors does not maximise reagent cost savings, but it improves culture reproducibility, within and across laboratories, and therefore saves labour costs and time. In our laboratory we also opted to use a liquid formulation of DMEM/F12 as the basis for B8+ and hE8, since the batches of powder media resulted in liquid media with varying pH and osmolarity that, despite our adjustment, led to inconsistent cell growth. This seems to be an issue also experienced by others ^37^. With these modifications, the cost of the medium is ~80€/L of B8+ and ~100€/L of hE8, compared to ~650€/L for cE8, assuming cytokines are purchased at the microgram scale described in our methods and priced at current list rates. Bulk purchases at the milligram scale can reduce costs even further, to roughly €20/L for B8+ and €30/L for hE8. While the upfront investment may be prohibitive for smaller laboratories, this strategy could yield substantial savings when implemented at the institutional level. In addition, weekend-free media use only 57% of the volume that the daily-change medium uses during a week of culture, which lowers the effective costs even more.

hiPSCs grown in weekend free media are in many aspects similar to those grown in daily-changed cE8. The cells maintain pluripotency, as shown by the high percentage of NANOG and OCT4 double-positive cells and by their ability to differentiate towards three germ layers. In addition, both weekend-free media supported the pluripotency of different hiPSC and hESC lines. The media were also compatible with more complex organoid differentiations representing all three germ layers: neuroectodermal (cortical organoids), mesodermal (cardioids) and endodermal (colon organoids), though not all protocols performed identically or reproducibly across conditions. Importantly, both media formulations supported long-term cell culture (20+ passages) without evidence of genomic instability. In general, hE8 was not substantially different from cE8 in the key assays we performed, except for subtle transcriptional differences revealed by sensitive single-cell RNA-seq, which we speculate may simply reflect the different cell feeding regimes and did not seem to result in any overt functional impairment. We concluded that it is a safe and simple alternative to commercial media that does not require long adaptation, and indeed our laboratory has now switched to using this formulation for routine experimentation.

On the other hand, cells grown in B8+ show clear changes in cell and colony morphology. This may be related to their apparent neuroectoderm lineage-bias revealed by molecular analyses. Despite this, B8+-cultured hiPSCs remain pluripotent for at least 20 passages (we did not test this further) and could be differentiated into derivatives of all germ layers, although some differentiation protocols would require adjustment to account for increased cell state heterogeneity. The neuroectodermal bias could underlie the reduced size of cardiac organoids, as we previously observed depletion of ZIC1+ cells in this assay. By contrast, the enhanced formation of rounded gut organoids was counterintuitive. From a developmental biology perspective it is interesting that B8+, which in principle stimulates the same signalling pathways as hE8, results in such a marked increase in ZIC1 positive hiPSCs. This may result from TGF-β-independent activities of TGF-β3 versus TGF-β1, or the slightly different levels of these and other molecules (i.e., ascorbic acid, transferrin, insulin, and NRG1).

One of the potential advantages for growing cells in B8+ is a higher efficiency of genome editing. We speculate that this is primarily linked to morphology changes, as less compact cells have better exposure to the transfection reagents. Indeed, we observed a higher efficiency of transient transfection, correlating with a higher number of genome-edited cells. B8+ can thus be an attractive alternative to commercial media for large-scale genome editing experiments. Nevertheless, we warrant caution because we have not determined whether cells genome edited in these conditions acquire an epigenetic signature that may complicate subsequent experiments ^14^.

Our study has several limitations that should be considered. First, validation in three hPSC lines cannot substitute for testing across the hundreds of lines maintained worldwide, and broader benchmarking — ideally through multi-center efforts or consortia — will be needed to establish general utility. Similarly, although we demonstrated differentiation into four lineages representing all three germ layers, additional validation in other widely studied cell types such as hepatocytes, kidney, or pancreas will be important. Most of our differentiation experiments were not powered to detect subtle differences, and should be viewed as proof of principle rather than definitive. Laboratories wishing to adopt hE8 or B8+ are encouraged to proceed with care, benchmarking pluripotency and differentiation efficiency against their existing protocols to ensure reproducibility. Finally, while CNV analyses did not reveal overt genomic instability, we cannot exclude the possibility that intermittent feeding influences mutational burden, which will need to be addressed in future studies, particularly for translational applications. Overall, the use of rigorously tested commercial media remains the safest approach for non-expert laboratories to establish robust protocols with fewer variables, before considering a switch to cost-effective homemade formulations. In doing so, we recommend adhering to the standards of the International Society for Stem Cell Research (ISSCR) for the use of human stem cells in basic research, which represent the current consensus on the level of rigor required for characterization and reporting ^38^.

Despite these caveats, our findings provide a practical path toward reducing the cost and workload of hPSC culture for expert laboratories, and we hope they will serve as a foundation for broader community efforts to democratize access to pluripotent stem cell technologies for basic and translational research. Looking further ahead, once clinical trial bottlenecks ease, the cost and accessibility of cell therapies will become increasingly important. In that context, flexible media prepared in-house from high-quality reagents may help lower barriers to access, provided their use is aligned to regulatory requirements and coupled with rigorous benchmarking against established standards. A clear understanding of the contribution of individual medium components, and the simplification of formulations where possible, can also facilitate technology transfer to contract development and manufacturing organizations (CDMOs) or academic good manufacturing practices (GMP) facilities. Unlike proprietary commercial formulations, open and well-characterized recipes provide transparency that can accelerate GMP process optimization. At the same time, raw materials for GMP production must be animal-origin–free, fully traceable, and manufactured under strict quality systems. It should be noted that there is no such thing as a formally ‘GMP’ raw material unless it is itself a therapeutic product or excipient; the guiding principle is to use the best available reagents and ensure their risk assessment within a quality management system. In this context, homemade growth factors are unlikely to meet the required standards and would need to be replaced by certified commercial reagents for clinical translation. Finally, weekend-free or “flexible feeding” media should be evaluated with caution, as reduced feeding schedules may influence cellular phenotypes ^46^, which our study confirms by showing the change in the metabolism-related transcriptional profile in weekend-free media growing cells Together, these considerations underscore that while homemade weekend-free formulations offer immediate value in basic research and may inform the design of transparent, simplified media for future technology transfer, their use will require careful benchmarking and regulatory oversight.

## Ethics and consent

Commercially available human embryonic stem cell lines (H1 and H9; WiCell; SLA #17-W0261) were used under existing institutional oversight and in compliance with applicable national regulations. These lines were derived abroad before 2007 and are therefore permissible for use in Italy under current legislation. All experiments were also compliant with the International Society for Stem Cell Research and Clinical Translation ( https://www.isscr.org/guidelines).

## Data Availability

Zenodo: Underlying and Extended Data for Refined and benchmarked homemade media for cost-effective, weekend-free human pluripotent stem cell culture. DOI: https://zenodo.org/doi/10.5281/zenodo.12684584 ^6^ This project contains the following data: Extended Data 1.pdf - protocol for preparation of the supplement for hE8 and B8+ media Extended Data 2.zip - Gene counts, supporting files, and output results of bulk analyses Extended Data 3.zip - Images of iPS cells adapted to cE8, hE8 and B8+ taken 24, 48 and 72 hours after passage. Contains raw .tiff files for each image and a .pdf with a compiled figure Extended Data 4.zip - Differential distribution analysis comparing monocle clusters composition between cells adapted to hE8 and cE8 or cells adapted to B8+ and cE8 Manuscript Data.zip – Raw data underlying the Figures 1, 2, 4, 5, 6 and 7. Bulk_RNA_seq_archive – archived source code used for generating results in Figure 3 Data are available under the terms of Creative Commons BY 4.0 licence. Uniprot: Data entry for human FGF2. Accession number P09038; https://www.uniprot.org/uniprotkb/P09038/entry ^40^ Uniprot: Data entry for human Activin A. Accession number P08476; https://www.uniprot.org/uniprotkb/P08476/entry ^41^ Uniprot: Data entry for human TGF-b1. Accession number P01137; https://www.uniprot.org/uniprotkb/P01137/entry ^42^ Uniprot: Data entry for human TGF-b3. Accession number P10600; https://www.uniprot.org/uniprotkb/P10600/entry ^43^ Uniprot: Data entry for human NRG1. Accession number Q02297; https://www.uniprot.org/uniprotkb/Q02297/entry ^44^ Biostudies: RNA-seq data generated in the course of this work (raw data underlying Figure 3). Accession number E-MTAB-14237; https://www.ebi.ac.uk/biostudies/arrayexpress/studies/E-MTAB-14237 ^45^ Biostudies: Single cell RNA-seq data generated in the course of this work (raw data underlying Figure 4). Accession number E-MTAB-15570; https://www.ebi.ac.uk/biostudies/arrayexpress/studies/E-MTAB-15570 ^46^

## Software availability

The docker image in the dockerhub repository that contains all the software for the analysis is available from: https://hub.docker.com/r/hedgelab/rstudio-hedgelab/tags, pulling the image with ‘media_bulk’ tag

The code used for analysis of data presented in Figure 2J, Figure 3 and Figure 4 are available from: https://github.com/alessandro-bertero/homemade_media_code_repository

Archive source code available from: https://zenodo.org/doi/10.5281/zenodo.12684584 ^6^

License: CC BY 4.0

GenomeStudio 2.0.5, the software used for CNV detection, is a proprietary software. It is available after creating a free account from: https://emea.support.illumina.com/array/array_software/genomestudio/downloads.html

The tool to analyse qPCR results is available online after creating a free account on Thermo Fisher cloud from: https://apps.thermofisher.com/apps/spa/#/dashboard

GraphPad Prism 10 ( https://www.graphpad.com), the software used for statistical analysis, is a proprietary software. Free and open-source alternatives are available, such as SOFA Statistics Open For All ( https://www.sofastatistics.com/home.php) or JASP ( https://jasp-stats.org).

FlowJo 10 ( https://www.flowjo.com/solutions/flowjo), the software used for the analysis of flow cytometry data, is a proprietary software. Free alternatives are available, such as Floerada online tool ( https://floreada.io) or FCSalyzer ( https://sourceforge.net/projects/fcsalyzer/).
