# Supplementary material for: Refined home-brew media for cost-effective, weekend-free hiPSC culture and genetic engineering
Source: Open Res Eur. 2024 Sep 2;4:192. [Version 1] doi: 10.12688/openreseurope.18245.1 (PMC12488189; doi:10.12688/openreseurope.18245.1)
Supplement: Peer review history

# Response

**Published:** 2025-09-08

**Authors:** Alessandro Bertero

Our revised manuscript addresses all critiques raised by the reviewers. In the sections below the respond **point by point to the reviewer’s comments**, citing specific **figures** and *text* for their convenience. The manuscript was additionally edited for clarity and flow, and all changes are indicated by tracked changes in the marked up submission.

 Reviewer 1:

 Review Commentary on Truszkowski et al.

 The study presented by Truszkowski et al. aims to develop a cost-effective, reproducible, and practical method for culturing human induced pluripotent stem cells (hiPSCs). The authors sought to address the limitations of existing media, such as Basal 8 (B8), by refining it to create a modified version called B8+, which uses readily available, animal-free growth factors. The goal was to evaluate B8+ against other media in applications such as iPSC culturing, genome editing, and cardiac differentiation. However, several areas require further attention before their findings can be considered definitive.

**We thank the reviewers for their evaluation and thoughtful critiques, which have been instrumental in refining our conclusions. During the course of this extensive revision—carried out with broader testing and additional contributions from collaborating laboratories—we generated new data that prompted a reappraisal of our findings. While B8+ displayed certain potential advantages (e.g., higher NANOG expression and more efficient genome editing), single-cell transcriptomic analyses revealed that these benefits are offset by strong lineage priming that may limit its general utility. By contrast, the modified in-house E8 formulation (hE8), which incorporates some of the improvements originally introduced in B8, performed comparably to commercial E8 across most assays and in different hPSC lines, while enabling weekend-free culture with fewer compromises (e.g., the need for fine-tune certain differentiation protocols).Reflecting this evidence, we now highlight hE8 as the more suitable option for broad adoption while advising caution regarding the use of B8+. To support this revised conclusion, we have added pluripotency and trilineage differentiation assays, extended media validation to additional hESC lines, performed genomic stability analysis after long-term culture, increased biological replication and re-analysed statistics with mixed models, and included new single-cell RNA-seq datasets.**

 General Observations

 The main concern with the manuscript is that all the experiments were performed using a single cell line (WTC11, passage 50-70). Although this line is a standard for such studies, the results cannot be broadly generalized without replication in other hiPSC or hESC lines. The authors do acknowledge this limitation, but it needs more emphasis. The reliance on one cell line limits the reproducibility and applicability of their findings. Therefore, the conclusions should be considered preliminary, as more data from other laboratories and cell lines are essential to solidify the claims made.

**Our goal was to characterize in depth various media formulations in a reference cell line, not to claim broad applicability across all hPSCs, which would require testing dozens of lines and was beyond the scope of our study. We now explicitly acknowledge this limitation in the Discussion as follows:**

*”Our study has several limitations that should be considered. First, validation in three hPSC lines cannot substitute for testing across the hundreds of lines maintained worldwide, and broader benchmarking — ideally through multi-center efforts or consortia — will be needed to establish general utility.”*

**Nevertheless, because this valid concern was also raised by other reviewers, we undertook additional experiments with other hPSC lines in collaboration with the Oliviero and Ditadi laboratories in Torino and Milano. Key experiments of hPSC expansion in the three media formulations were reproduced in two hESC lines, one female (H9) and one male (H1). In all the media, both  cell lines maintained pluripotency, as shown by NANOG and OCT4 flow cytometry ( Fig. 7A,C). These lines were cultured in different tissue culture setups under distinct SOPs—H9 in hypoxia and H1 in normoxia— further demonstrating the robustness of weekend-free culture. Moreover, both lines retained differentiation potential, generating hematopoietic progenitor cells (H1,** **Fig. 7B** **) and cortical organoids (H9,** **Fig. 7D** **).**

 Reproducibility and Reliability

 The authors emphasize that their goal was to develop a medium that is accessible to non-expert groups. However, using home-made media formulations could introduce additional variability, especially in settings where culturing expertise is limited. It is worth noting that custom formulations should only be adopted when the culturing process is well-validated and benchmarked against a consistent protocol to ensure reproducibility. Moreover, the introduction suggests that this medium could be suitable for clinical applications, but this may be misleading. Custom-made reagents are unlikely to meet the stringent GMP regulations required for cell therapies. Thus, it would be prudent to revise claims about the translational potential of B8+ for clinical use.

**The reviewers raised two critical points which were not sufficiently addressed in our original submission. With respect to adoption of weekend-free media by non-expert laboratories, we have added the following to the Discussion:**

*“Overall, the use of rigorously tested commercial media remains the safest approach for non-expert laboratories to establish robust protocols with fewer variables, before considering a switch to cost-effective homemade formulations. In doing so, we recommend adhering to the standards of the International Society for Stem Cell Research (ISSCR) for the use of human stem cells in basic research, which represent the current consensus on the level of rigor required for characterization and reporting.“*

**With respect to clinical applications, we have removed premature references to this aspect from the Abstract, Introduction, and Results, but we have retained a balanced discussion of the topic, which we believe is important and rarely addressed in the literature:**

*“Looking further ahead, once clinical trial bottlenecks ease, the cost and accessibility of cell therapies will become increasingly important. In that context, flexible media prepared in-house from high-quality reagents may help lower barriers to access, provided their use is aligned to regulatory requirements and coupled with rigorous benchmarking against established standards. A clear understanding of the contribution of individual medium components, and the simplification of formulations where possible, can also facilitate technology transfer to contract development and manufacturing organizations (CDMOs) or academic good manufacturing practices (GMP) facilities. Unlike proprietary commercial formulations, open and well-characterized recipes provide transparency that can accelerate GMP process optimization. At the same time, raw materials for GMP production must be animal-origin–free, fully traceable, and manufactured under strict quality systems. It should be noted that there is no such thing as a formally ‘GMP’ raw material unless it is itself a therapeutic product or excipient; the guiding principle is to use the best available reagents and ensure their risk assessment within a quality management system. In this context, homemade growth factors are unlikely to meet the required standards and would need to be replaced by certified commercial reagents for clinical translation.  Finally, weekend-free or “flexible feeding” media should be evaluated with caution, as reduced feeding schedules may influence cellular phenotypes, which our study confirms by showing the change in the metabolism-related transcriptional profile in weekend-free media growing cells. Together, these considerations underscore that while homemade weekend-free formulations offer immediate value in basic research and may inform the design of transparent, simplified media for future technology transfer, their use will require careful benchmarking and regulatory oversight.”*

 Media Performance and Differentiation Potential

 The study shows that B8+, while stimulating the same pathways as hE8, induces morphological changes and appears to prime cells for mesendoderm differentiation. The concern is that there is insufficient evidence supporting the use of TGF-β3 over TGF-β1. Given that TGF-β3 had to be increased from 0.1 ng/ml (as used in the original B8 formulation) to 1 ng/ml to maintain pluripotency, this cost-saving advantage is nullified. The authors should clarify that at this concentration, TGF-β3 does not offer significant benefits over TGF-β1, which is widely accepted for maintaining pluripotency in TeSR1 media Ref 1

 Furthermore, the findings suggest B8+ may skew cells towards mesendoderm lineages, particularly raising concerns for iPSC differentiation into neural lineages, which may require additional adjustments to overcome mesodermal priming. The authors note this issue in the final part of their discussion but should emphasize that B8+ cannot yet be recommended as a safe or universal alternative to commercial media without further validation. Additional studies examining differentiation into all three germ layers—mesoderm, endoderm, and ectoderm—are crucial to confirm the medium's effectiveness in maintaining pluripotency and its versatility in guiding differentiation.

**This was an insightful remark that prompted additional experiments and careful consideration of the key aspects involved in comparing TGF-β3 with TGF-β1 for pluripotency maintenance, namely cost efficiency and ease of production, and potential lineage biases. Taken together, these evaluations led us to refine our conclusions and adjust our recommendation regarding the most suitable medium for broad use.**

**From an economic perspective, TGF-β1 and TGF-β3 are similarly priced by most manufacturers. In our B8+ formulation, pluripotency was maintained with TGF-β3 at 1 ng/mL, which—although a 10-fold increase compared to the original B8 recipe—remains half the concentration of TGF-β1 required in cE8 (2 ng/mL). The increased use of TGF-β3 is largely offset by the 8-fold reduction in thermostable FGF2 (from 40 to 5 ng/mL), made possible by the use of a truncated, tag-free, rigorously biomanufactured FGF2-G3. We applied the same cost-saving adjustment to cE8, but in the absence of additional benchmarking we do not recommend reducing TGF-β1 further, consistent with the original B8 manuscript reporting that “[TGF-β1] could not be used at lower concentrations than previously suggested (2 ng/mL) when using our five-passage assay (Figure S3A)”. Ref 1**

**With respect to manufacturing, the same manuscript noted that “Comparing TGF-b1, TGF-b1m, TGF-b3, and TGF-b3m, we found that TGF-b3 offered the best combination of being able to be produced in E. coli and suitable for use at 0.1 ng mL1 (Figures 3I, S3C, and S3D) and was therefore selected for the final formula”. However, the same group’s updated STAR protocol recommended sourcing TGF-β3 commercially since: “we found that production of TGFB3 and NRG1 resulted in much lower yields than FGF2-G3 and, in the case of NRG1 require the 6xHis tag to be cleaved further reducing yield. For the small amounts of these two proteins to be used, we have not found it cost-effective to make them in house” Ref 2. While various companies produce both molecules, the manufacturer we partnered with to develop our weekend-free media, Qkine, is the only one we are aware of that produces high-quality TGF-β1 using E. coli. All other sources we are familiar with rely on eukaryotic systems such as engineered CHO cells. This aligns with the original B8 observation that TGF-β1 is more complex to produce bacterially, likely because its bioactivity depends on achieving a higher fraction of dimeric protein, which is difficult to obtain in non-expert laboratories.**

**Our updated discussion synthesized these two points as follows:**

*“To overcome this issue, we developed the B8+ formulation, which relies on a 10-fold higher concentration of TGF-β3 but allows an 8-fold lower concentration of FGF2-G3, largely offsetting the increased cost. TGF-β1 and TGF-β3 are generally considered equivalent in supporting hPSC growth, although TGF-β3 can be more potent in certain contexts such as cell migration. In our B8+ formulation, pluripotency was maintained with TGF-β3 at 1 ng/mL. While this is higher than the 0.1 ng/mL reported in the original B8 recipe, it remains half the concentration of TGF-β1 used in cE8 (2 ng/mL), which has been reported as necessary for maintaining pluripotency. We therefore retained TGF-β3 in B8+, as it supports pluripotency at a lower concentration than TGF-β1, even if the cost advantage compared to cE8 is more modest than originally anticipated. Moreover, the original B8 formulation employed TGF-β3, reportedly easier to express in bacteria than TGF-β1, although later work from the same group recommended sourcing TGF-β3 commercially because its low yield made in-house production not cost-effective. Notably, all growth factors used in this study were produced in E. coli, indicating that expert biomanufacturing can mitigate this limitation.”*

**With regard to potential lineage biases, we performed the experiments recommended by the reviewers and observed that hiPSCs adapted to cE8, hE8, and B8+ differentiated comparably in a trilineage assay ( Fig. 5D). In addition to expanding our analyses of mesoderm-derived cardiac differentiation (see below), we confirmed their ability to generate mature endodermal derivatives such as colon organoids ( Fig. 6). We also found that a hematopoietic differentiation protocol optimized for H1 hESCs maintained in cE8 could be readily applied to cells adapted to weekend-free media ( Fig. 7B). For neuroectoderm, a protocol previously optimized for H9 hESCs was successful in all conditions ( Fig. 7D). These proof-of-principle experiments demonstrate that, despite potential differences in lineage priming, established protocols for differentiation into all three germ layers can be implemented across conditions. Nonetheless, in the Discussion we explicitly acknowledge the limitation that:**

*“Similarly, although we demonstrated differentiation into four lineages representing all three germ layers, additional validation in other widely studied cell types such as hepatocytes, kidney, or pancreas will be important. Most of our differentiation experiments were not powered to detect subtle differences, and should be viewed as proof of principle rather than definitive.”*

**Given these results, that suggested limited if any differentiation bias in B8+, we decided to re-examine our previous RNA-seq analyses which had led us to the possibly premature conclusion of a clear lineage bias. While we stand by the rigour of those analyses, in hindsight they were of limited value for two reasons: (1) they did not account for potential cell heterogeneity; (2) the biological conclusions were based on Gene Ontology lists of developmentally-associated genes, which may not reflect those most relevant to human PSC biology. To mitigate both issues we performed a single cell experiment analysing two independent adaptations to each media, and integrated the resulting data with existing datasets of hiPSCs annotated based on their lineage biases and recent human early embryonic development. The results, described in** **Figure 5** **and the relative results text copied below for ease of the reviewers:**

*“Intrigued by the coarse differences observed in bulk RNA-seq, we next sought finer resolution by performing single-cell transcriptomic analyses to dissect population heterogeneity across media conditions. Using 10x Genomics microfluidics, we profiled >2,000 cells per condition in two biological replicates. Aggregated analysis revealed an unexpected degree of diversity, with at least four clearly distinct subpopulations (Fig. 4A).*

*All subpopulations expressed similar levels of the core pluripotency factors POU5F1/OCT4 and SOX2, but differed in the levels of NANOG, which was highest in a population that paradoxically co-expressed the neuroectoderm competence factor ZIC1 (Fig. 4B). We recently showed that ZIC1+ hiPSC clones are biased towards neuroectodermal lineages, leading to their depletion during cardiac differentiation and altered specification in forebrain derivatives12. While those observations were made in mTeSR Plus, an unrelated medium formulation, integrative analysis of the two datasets confirmed a strong similarity between the ZIC1+ populations (Fig. 4C). Accordingly, the top surface marker we had previously identified, UNC5D, was exclusively expressed in this population, which we tentatively labelled iPSC-neuro.*

*A second, smaller subpopulation corresponded to cells we had previously observed only in hiPSCs transiently primed for mesodermal fate by WNT signalling activation (Fig. 4C). Consistently, this cluster specifically expressed LRRC75A (Fig. 4B), one of the few markers we could assign to this transient state. To further examine this state, which had not been extensively characterized in our earlier work, we compared its transcriptional profile with that of the developing human gastrula15,16. Cells from this cluster appeared more developmentally advanced (Fig. 4D), and displayed a strong transcriptional signature characteristic of nascent mesoderm (Fig. 4E). Based on these features, we identified this cluster as iPSC-meso.*

*The two remaining major subpopulations, designated iPSC-1 and iPSC-2, had not been characterized in our previous studies. To exclude the possibility that these reflected different cell cycle states, we examined proliferation signatures: both clusters contained cells with high S and G2/M indices (Fig. 4F), and expression of proliferation markers such as MKI67 was distributed across all four subpopulations (Fig. 4B), ruling out a simple cell cycle-based artifact. Gene ontology analyses of differentially expressed genes between iPSC-1 and iPSC-2 revealed a mild enrichment of morphogenesis-associated terms in iPSC-1 (Fig. 4G), while iPSC-2 was strongly enriched for biosynthetic processes (Fig. 4H). For example, iPSC-1 showed elevated expression of the BMP target ID1 and the early mesenchymal marker VIM, whereas iPSC-2 expressed higher levels of the glycolytic enzyme LDHA (Fig. 4B,I).*

*Having established a tentative interpretation of subpopulation diversity, we next examined their relative abundance across the three media conditions (Fig. 4J,K). iPSC-1 represented the majority of cells in both hE8 and B8+, in contrast to cE8, where iPSC-2 was strongly predominant. These results indicate that weekend-free culture induces a subtle but consistent shift in hiPSC transcriptomes, likely reflecting changes in metabolic state and/or autocrine signalling associated with intermittent media starvation.*

*Despite these similarities between the two weekend-free media, we observed a striking difference with regard to the iPSC-neuro population. While hE8 slightly reduced its representation compared to cE8 to less than 10%, B8+ increased it to ~40% of the population (Fig. 4K). An orthogonal analysis with Milo17 of differential abundance at the neighbourhood level confirmed these shifts across replicates (Extended Data 46). Thus, while both weekend-free formulations affect the metabolic state of hiPSCs, B8+ specifically results in a neuroectoderm lineage bias.”*

**These results prompted us to revise our earlier conclusions based on bulk RNA-seq, and the corresponding GO and GSEA analyses were removed from the manuscript to avoid potential confusion.**

**Altogether, weighing the modest cost advantage and relative ease of production of TGF-β3 against the reduced lineage priming observed with TGF-β1, our ultimate recommendation favors hE8 for most applications. This formulation ensures smoother transition of cells adapted from cE8 with minimal compromises, limited primarily to fine-tuning of specific differentiation protocols.**

 Statistical Considerations

 Another significant limitation lies in the statistical robustness of the results. For instance, in Figure 4G and 4F, the authors present data on TNNT2 expression in cardiac organoids. However, with experiments performed only in duplicate (n=2), it is premature to draw solid conclusions about TNNT2 expression or any other findings from this limited dataset. Larger sample sizes are necessary to provide more confidence in these results and support statistically significant conclusions.

**This is a valid concern, but we wish to clarify that in our initial submission several experiments where only two data points were shown actually represented averages of two to three independently adapted cultures, examined across two independent experimental rounds. This design was intended to capture both intra- and inter-experimental variability. In the revised manuscript, we now present each data point explicitly and apply a mixed-effects model that accounts for both levels of biological replication as separate potential sources of variance (e.g.** **Fig. 2H–I** **).**

**With regard to the specific experiment highlighted by the reviewer, we increased the number of independent experiments to n = 4, analyzing two independent media adaptations in each, and re-analyzed the results with the same mixed model ( Fig. 5H–I). These results confirmed that B8+ supported differentiation into a comparable percentage of TNNT2⁺ cells in cardiac organoids, albeit with consistently lower expression of this marker. By contrast, cells in hE8—although previously differentiating efficiently in this assay and performing similarly to cE8 in all other functional tests, including those performed with the same batch of hE8 prepared for the revision experiments—failed to differentiate at high efficiency in the new replicates. While we were unable to identify the variable underlying this discrepancy, we report these data to highlight an important point: as anticipated by the reviewers, one of the risks of homemade media is that even minor differences in preparation can increase variability in experimental outcomes, which may not be apparent when assessing only pluripotency markers.**

**Lastly, although not specifically requested by the reviewers, we repeated the genome editing assay, as this was one of the experiments that had shown preliminary differences between B8+ and the other conditions. The new results confirmed that B8+ yields a higher number of genome-edited colonies ( Fig. 5B). We did not systematically repeat other assays that had shown no trend toward differences across media conditions, as we considered additional repetitions unlikely to provide further insight.**

 Weekend-Free Culture Considerations

 While the authors claim that B8+ enables a weekend-free workflow, the study only evaluates the medium's stability over a 48-hour interval. A weekend-free culture system generally implies a 72-hour window. Therefore, the authors should re-evaluate the medium over longer intervals to validate the feasibility of this claim.

**We wish to clarify that our weekend-free protocol involves passaging cells on Thursday afternoon, feeding them on Friday after ~24 hours to remove thiazovivin, and then passaging again on Monday morning. In practice, this corresponds to a 60–64 hour interval, even for researchers following a standard 9 am-5 pm workday schedule.**

**The reviewers’ point remains valid, however, and accordingly we evaluated the stability and bioactivity of both full-length and truncated FGF2-G3, as well as TGF-β1, over a 7-day period as a stress test. These assays showed only minor changes in performance of FGF2-G3 after 7 days ( Fig. 1A,B). Together with our previous and new experiments demonstrating robust adaptation and long-term expansion of hPSC lines—including hiPSCs maintained through at least 20 passages—these results support that both weekend-free media provide a suitable signaling environment for sustained hPSC culture** **.**

 Conclusion

 Overall, the study provides a promising alternative medium for hiPSC culture with B8+, but several limitations prevent it from being widely recommended at this time. The reliance on a single cell line, insufficient sample sizes, and potential issues with mesendoderm priming are key factors that need addressing. Additionally, the cost-benefit of using TGF-β3 versus TGF-β1 should be reconsidered, given that the necessary increase in TGF-β3 concentration negates its cost-effectiveness. The findings should be framed as preliminary, and further testing in multiple cell lines and under various conditions is crucial for validating the reproducibility and broader application of B8+.

**We thank the reviewers for their fair and constructive assessment of our original manuscript. While this project began as an internal effort to optimize protocols that we could not reproduce in our own hands, the depth of benchmarking ultimately performed convinced us it would be valuable to share these results with the community, both to provide systematic comparisons and to alert others to potential pitfalls.**

**The reviewer’s feedback prompted us to expand and strengthen the study, as detailed above and in the responses to the other reviewers (see in particular the new genomic stability results,** **Fig. 2J** **). We hope that, with these revisions, the manuscript now serves as a balanced resource for the community, which until now has largely relied on either commercial media or informally shared homebrew formulations—such as our own initial use of Essential 8—that have not been extensively benchmarked.**

**With regard to cost-effectiveness, we have also updated the Discussion to describe further savings achievable through bulk purchasing:**

**

*“With these modifications, the cost of the medium is ~80€/L of B8+ and ~100€/L of hE8, compared to ~650€/L for cE8, assuming cytokines are purchased at the microgram scale described in our methods and priced at current list rates. Bulk purchases at the milligram scale can reduce costs even further, to roughly €20/L for B8+ and €30/L for hE8. While the upfront investment may be prohibitive for smaller laboratories, this strategy could yield substantial savings when implemented at the institutional level. In addition, weekend-free media use only 57% of the volume that the daily-change medium uses during a week of culture, which lowers the effective costs even more.”*

***References*** *:*

*1.         Kuo, H.-H. et al. Negligible-Cost and Weekend-Free Chemically Defined Human iPSC Culture. Stem Cell Reports 14, 256–270 (2020).*

*2. Lyra-Leite, D. M., Fonoudi, H., Gharib, M. & Burridge, P. W. An updated protocol for the cost-effective and weekend-free culture of human induced pluripotent stem cells. STAR Protocols 2, 100213 (2021).*
